# New records and range extensions of several species of native bees (Hymenoptera: Apoidea) from Mississippi

**DOI:** 10.3897/BDJ.6.e25230

**Published:** 2018-05-17

**Authors:** Katherine Parys, Terry Griswold, Harold W Ikerd, Michael Christopher Orr

**Affiliations:** 1 USDA ARS, Stoneville, MS, United States of America; 2 USDA ARS, Logan, UT, United States of America; 3 Key Laboratory of Zoological Systematics and Evolution, Chinese Academy of Sciences, Beijing, China

**Keywords:** Biodiversity, Apidae, Andrenidae, Halictidae

## Abstract

**Background:**

The native bee fauna of Mississippi, USA has been historically poorly sampled, but is of particular relevance to determine range limits for species that occur in the southern United States. Currently published literature includes 184 species of bees that occur within the state of Mississippi. Additions to the list of native bees known for Mississippi are reported with notes on range, ecology and resources for identification.

**New information:**

The geographic ranges of seven additional species are extended into the state of Mississippi: Andrena (Melandrena) obscuripennis Smith, 1853, *Anthemurgus passiflorae* Robertson, 1902, *Dieunomia bolliana* (Cockerell 1910), Diadasia (Diadasia) enavata (Cresson 1872), *Peponapis crassidentata* (Cockerell 1949), *Triepeolus subnitens* Cockerell and Timberlake, 1929 and *Brachynomada nimia* (Snelling and Rozen 1987). These records raise the total number of published species known from the state to 191. *Anthemurgus* and *Brachynomada* are also genera new to Mississippi.

## Introduction

The native bee fauna of Mississippi is poorly known and sampled, but is of particular relevance to determining range limits of many species (Smith et al. 2012). Mississippi is composed of four distinct ecoregions: the Southeastern Plains, the Mississippi Alluvial Plain, the Mississippi Valley Loess Plains and the Southern Coastal Plain (Chapman et al. 2004).

The majority of the recorded bee species currently known from Mississippi are from Mitchell, who summarised state level distributions across the eastern United States and recorded 122 species from Mississippi (Mitchell 1960, Mitchell 1962). The majority of the records included therein are those from collections made by Michener in the 1940s near Hattiesburg, MS, in the South-eastern Plains (Michener 1947). Smith et al. (2012) listed 53 more records from the Black Belt Prairies, also part of the South-eastern Plains, while Rightmyer (2008) listed an additional five species in a revisionary study of the cleptoparasite *Triepeolus* Robertson. An additional series of papers (MacGown and Scheifer 1992, Cane et al. 1996, Colla et al. 2011, Parys et al. 2015) each added singular records, bringing the published total number of species reported from the state of Mississippi to 184.

Of the four ecoregions that occur within the state, the Mississippi Alluvial Plain is of particular interest as it is almost completely un-sampled for native bee fauna with the exception of Parys et al. (2015) and is part of the Mississippi Alluvial Valley (MAV) which also includes portions of Arkansas, Louisiana and Missouri. This region of Mississippi is colloquially referred to as the "Delta" of the state. The MAV is the largest floodplain in the United States, comprising over 10 million hectares of historically bottomland hardwood forest that was seasonally flooded (Frederickson 2005). Today, the majority of the floodplain has been controlled with a system of levees constructed during the twentieth century, allowing the majority of the landscape to be converted to commercial agriculture (Faulkner et al. 2011). Landscapes fragmented by agriculture generally have less biodiversity than those left as natural habitats, though mass flowering crops can influence the densities of generalist pollinators (Westphal et al. 2003, Potts et al. 2010). Baseline data on the presence and distribution of native bee species of these previously unsampled areas across the MAV can inform decision-making by land managers and potentially be used to assess risks from agricultural practices. This study aims to examine the biodiversity and community structure of pollinators utilising both natural habitats and commercially managed agricultural fields.

## Materials and methods

Collections of bees from a variety of habitats across the Mississippi Delta were made between 2015 and 2017. Locations sampled included commercial agricultural operations, research farms operated by the United State Department of Agriculture's Agricultural Research Service and local universities and two national wildlife refuges. Commercial farms in the Mississippi Delta typically plant a combination of cotton (*Gossypium hirsutum* L.), corn (*Zea mays* L.) and soybeans (*Glycine max* (L.) Moench.). Many of the commercial farms also plant smaller fields of sunflowers (*Helianthus annuus* L.), sorghum (*Sorghum bicolor* L.), rice (*Oryza sativa* L.) and sweetpotato (*Ipomoea batatas* (L.) Lam. Collection methods at all of the locations included multiple methods from the following: modified pan traps (blue, yellow and white "bee bowls"), blue and yellow vane traps, malaise traps, netting, sweeping and examining bycatch from other collection methods.

Identifications were completed by the authors using a variety of primary literature (e.g. Cockerell 1910, Mitchell 1960, Mitchell 1962, Snelling and Rozen 1987, Michener 2007, Rightmyer 2008, Ayala and Griswold 2012, Bouseman and LaBerge 1978, Sipes 2001). Full occurence data for the seven bee species new to Mississippi is provided below.

## Checklists

### New records of bees from Mississippi

#### Andrenidae

#### Andrena (Melandrena) obscuripennis

Smith, 1853

##### Materials

- **Type status:** Other material. **Occurrence:** catalogNumber: SIMRU1529; recordedBy: Nathan Little; individualCount: 1; sex: female; lifeStage: adult; preparations: pin; occurrenceID: urn:USDA-ARS:SIMRU:SIMRU1529; **Taxon:** scientificName: Andrena (Melandrena) obscuripennis Smith, 1853; kingdom: Animalia; phylum: Arthropoda; class: Insecta; order: Hymenoptera; family: Andrenidae; genus: Andrena; subgenus: Melandrena; specificEpithet: obscuripennis; scientificNameAuthorship: Smith, 1853; **Location:** country: United States; stateProvince: Mississippi; county: Coahoma; locality: Dublin, Pheromone trap for lepidoptera; decimalLatitude: 34.051509; decimalLongitude: -90.502339; geodeticDatum: WGS1984; **Identification:** identifiedBy: H. Ikerd; dateIdentified: 2017; **Event:** samplingProtocol: Pheromone Bycatch; eventDate: 2015-5-28; **Record Level:** type: PhysicalObject; language: en; rights: https://creativecommons.org/publicdomain/zero/1.0/; accessRights: http://vertnet.org/resources/norms.html; institutionCode: USDA-ARS; collectionCode: SIMRU; basisOfRecord: PreservedSpecimen
- **Type status:** Other material. **Occurrence:** catalogNumber: SIMRU13237; recordedBy: Katherine A. Parys; individualCount: 1; sex: female; lifeStage: adult; preparations: pin; occurrenceID: urn:USDA-ARS:SIMRU:SIMRU13237; **Taxon:** scientificName: Andrena (Melandrena) obscuripennis Smith, 1853; kingdom: Animalia; phylum: Arthropoda; class: Insecta; order: Hymenoptera; family: Andrenidae; genus: Andrena; subgenus: Melandrena; specificEpithet: obscuripennis; scientificNameAuthorship: Smith, 1853; **Location:** country: United States; stateProvince: Mississippi; county: Bolivar; locality: Alcorn Research Farm, Mound Bayou; decimalLatitude: 33.871265; decimalLongitude: -90.699184; geodeticDatum: WGS1984; **Identification:** identifiedBy: H. Ikerd; dateIdentified: 2017; **Event:** samplingProtocol: Vane Trap (Blue); eventDate: 2017-5-10; **Record Level:** type: PhysicalObject; language: en; rights: https://creativecommons.org/publicdomain/zero/1.0/; accessRights: http://vertnet.org/resources/norms.html; institutionCode: USDA-ARS; collectionCode: SIMRU; basisOfRecord: PreservedSpecimen
- **Type status:** Other material. **Occurrence:** catalogNumber: SIMRU15308; recordedBy: Nathan Schiff; individualCount: 1; sex: female; lifeStage: adult; preparations: pin; occurrenceID: urn:USDA-ARS:SIMRU:SIMRU15308; **Taxon:** scientificName: Andrena (Melandrena) obscuripennis Smith, 1853; kingdom: Animalia; phylum: Arthropoda; class: Insecta; order: Hymenoptera; family: Andrenidae; genus: Andrena; subgenus: Melandrena; specificEpithet: obscuripennis; scientificNameAuthorship: Smith, 1853; **Location:** country: United States; stateProvince: Mississippi; county: Bolivar; locality: Alcorn Research Farm, Mound Bayou; decimalLatitude: 33.871265; decimalLongitude: -90.699184; geodeticDatum: WGS1984; **Identification:** identifiedBy: H. Ikerd; dateIdentified: 2017; **Event:** samplingProtocol: netting; eventDate: 2017-5-16; **Record Level:** type: PhysicalObject; language: en; rights: https://creativecommons.org/publicdomain/zero/1.0/; accessRights: http://vertnet.org/resources/norms.html; institutionCode: USDA-ARS; collectionCode: SIMRU; basisOfRecord: PreservedSpecimen

##### Notes

The currently published distribution of this species is recognised as Florida, Georgia, North Carolina and Ontario [Canada] (Bouseman and LaBerge 1978, Pascarella and Hall 2013, Woodcock et al. 2014). Mitchell (1960) originally included New Jersey and Louisiana as part of the distribution, but those records could not be validated by Bouseman and LaBerge (1978). Additional records from coastal areas of South Carolina, Virginia and Maryland are available online (Ascher and Pickering 2018). Little is known about the biology of this species, though in a study conducted in Georgia on the flight heights of bees, one individual was collected within 0.5 m of the ground while none were collected in the canopy (Ulyshen et al. 2010).

Of the three specimens reported here, all were female (Fig. 1). Two of the specimens were collected in Bolivar County at the Alcorn State University Research Farm located in Mound Bayou, MS. One of the specimens was collected in a blue vane trap while the other was netted from a fallow field. The third specimen was retrieved as bycatch from a baited Hartstack pheromone trap located in Coahoma County near Dublin, MS.

#### Anthemurgus passiflorae

Robertson, 1902

##### Materials

- **Type status:** Other material. **Occurrence:** catalogNumber: SIMRU15309; recordedBy: K. A. Parys; individualCount: 1; sex: female; lifeStage: adult; preparations: pin; occurrenceID: urn:YUTO:PCYU:SIMRU15309; **Taxon:** scientificName: Anthemurgus passiflorae Robertson, 1902; kingdom: Animalia; phylum: Arthropoda; class: Insecta; order: Hymenoptera; family: Andrenidae; genus: Anthemurgus; specificEpithet: passiflorae; scientificNameAuthorship: Robertson, 1902; **Location:** country: United States; stateProvince: Mississippi; county: Bolivar; locality: University Street, Cleveland; decimalLatitude: 33.73521; decimalLongitude: -90.73244; geodeticDatum: WGS1984; **Identification:** identifiedBy: K. A. Parys; dateIdentified: 2017; **Event:** samplingProtocol: netting; eventDate: 2017-6-18; **Record Level:** type: PhysicalObject; language: en; rights: https://creativecommons.org/publicdomain/zero/1.0/; accessRights: http://vertnet.org/resources/norms.html; institutionCode: YUTO; collectionCode: PCYU; basisOfRecord: PreservedSpecimen
- **Type status:** Other material. **Occurrence:** catalogNumber: SIMRU15310; recordedBy: K. A. Parys; individualCount: 1; sex: male; lifeStage: adult; preparations: pin; occurrenceID: urn:USDA-ARS:SIMRU:SIMRU15310; **Taxon:** scientificName: Anthemurgus passiflorae Robertson, 1902; kingdom: Animalia; phylum: Arthropoda; class: Insecta; order: Hymenoptera; family: Andrenidae; genus: Anthemurgus; specificEpithet: passiflorae; scientificNameAuthorship: Robertson, 1902; **Location:** country: United States; stateProvince: Mississippi; county: Bolivar; locality: University Street, Cleveland; decimalLatitude: 33.73521; decimalLongitude: -90.73244; geodeticDatum: WGS1984; **Identification:** identifiedBy: K. A. Parys; dateIdentified: 2017; **Event:** samplingProtocol: netting; eventDate: 2017-6-18; **Record Level:** type: PhysicalObject; language: en; rights: https://creativecommons.org/publicdomain/zero/1.0/; accessRights: http://vertnet.org/resources/norms.html; institutionCode: USDA-ARS; collectionCode: SIMRU; basisOfRecord: PreservedSpecimen
- **Type status:** Other material. **Occurrence:** catalogNumber: SIMRU15311; recordedBy: K. A. Parys; individualCount: 1; sex: male; lifeStage: adult; preparations: pin; occurrenceID: urn:USDA-ARS:SIMRU:SIMRU15311; **Taxon:** scientificName: Anthemurgus passiflorae Robertson, 1902; kingdom: Animalia; phylum: Arthropoda; class: Insecta; order: Hymenoptera; family: Andrenidae; genus: Anthemurgus; specificEpithet: passiflorae; scientificNameAuthorship: Robertson, 1902; **Location:** country: United States; stateProvince: Mississippi; county: Bolivar; locality: University Street, Cleveland; decimalLatitude: 33.73521; decimalLongitude: -90.73244; geodeticDatum: WGS1984; **Identification:** identifiedBy: K. A. Parys; dateIdentified: 2017; **Event:** samplingProtocol: netting; eventDate: 2017-6-18; **Record Level:** type: PhysicalObject; language: en; rights: https://creativecommons.org/publicdomain/zero/1.0/; accessRights: http://vertnet.org/resources/norms.html; institutionCode: USDA-ARS; collectionCode: SIMRU; basisOfRecord: PreservedSpecimen
- **Type status:** Other material. **Occurrence:** catalogNumber: SIMRU15312; recordedBy: K. A. Parys; individualCount: 1; sex: female; lifeStage: adult; preparations: pin; occurrenceID: urn:YUTO:PCYU:SIMRU15312; **Taxon:** scientificName: Anthemurgus passiflorae Robertson, 1902; kingdom: Animalia; phylum: Arthropoda; class: Insecta; order: Hymenoptera; family: Andrenidae; genus: Anthemurgus; specificEpithet: passiflorae; scientificNameAuthorship: Robertson, 1902; **Location:** country: United States; stateProvince: Mississippi; county: Bolivar; locality: University Street, Cleveland; decimalLatitude: 33.73521; decimalLongitude: -90.73244; geodeticDatum: WGS1984; **Identification:** identifiedBy: K. A. Parys; dateIdentified: 2017; **Event:** samplingProtocol: netting; eventDate: 2017-6-18; **Record Level:** type: PhysicalObject; language: en; rights: https://creativecommons.org/publicdomain/zero/1.0/; accessRights: http://vertnet.org/resources/norms.html; institutionCode: YUTO; collectionCode: PCYU; basisOfRecord: PreservedSpecimen
- **Type status:** Other material. **Occurrence:** catalogNumber: SIMRU15313; recordedBy: K. A. Parys; individualCount: 1; sex: female; lifeStage: adult; preparations: pin; occurrenceID: urn:YUTO:PCYU:SIMRU15313; **Taxon:** scientificName: Anthemurgus passiflorae Robertson, 1902; kingdom: Animalia; phylum: Arthropoda; class: Insecta; order: Hymenoptera; family: Andrenidae; genus: Anthemurgus; specificEpithet: passiflorae; scientificNameAuthorship: Robertson, 1902; **Location:** country: United States; stateProvince: Mississippi; county: Bolivar; locality: University Street, Cleveland; decimalLatitude: 33.73521; decimalLongitude: -90.73244; geodeticDatum: WGS1984; **Identification:** identifiedBy: K. A. Parys; dateIdentified: 2017; **Event:** samplingProtocol: netting; eventDate: 2017-6-18; **Record Level:** type: PhysicalObject; language: en; rights: https://creativecommons.org/publicdomain/zero/1.0/; accessRights: http://vertnet.org/resources/norms.html; institutionCode: YUTO; collectionCode: PCYU; basisOfRecord: PreservedSpecimen
- **Type status:** Other material. **Occurrence:** catalogNumber: SIMRU15314; recordedBy: K. A. Parys; individualCount: 1; sex: male; lifeStage: adult; preparations: pin; occurrenceID: urn:YUTO:PCYU:SIMRU15314; **Taxon:** scientificName: Anthemurgus passiflorae Robertson, 1902; kingdom: Animalia; phylum: Arthropoda; class: Insecta; order: Hymenoptera; family: Andrenidae; genus: Anthemurgus; specificEpithet: passiflorae; scientificNameAuthorship: Robertson, 1902; **Location:** country: United States; stateProvince: Mississippi; county: Bolivar; locality: University Street, Cleveland; decimalLatitude: 33.73521; decimalLongitude: -90.73244; geodeticDatum: WGS1984; **Identification:** identifiedBy: K. A. Parys; dateIdentified: 2017; **Event:** samplingProtocol: netting; eventDate: 2017-6-18; **Record Level:** type: PhysicalObject; language: en; rights: https://creativecommons.org/publicdomain/zero/1.0/; accessRights: http://vertnet.org/resources/norms.html; institutionCode: YUTO; collectionCode: PCYU; basisOfRecord: PreservedSpecimen
- **Type status:** Other material. **Occurrence:** catalogNumber: SIMRU15315; recordedBy: K. A. Parys; individualCount: 1; sex: female; lifeStage: adult; preparations: pin; occurrenceID: urn:YUTO:PCYU:SIMRU15315; **Taxon:** scientificName: Anthemurgus passiflorae Robertson, 1902; kingdom: Animalia; phylum: Arthropoda; class: Insecta; order: Hymenoptera; family: Andrenidae; genus: Anthemurgus; specificEpithet: passiflorae; scientificNameAuthorship: Robertson, 1902; **Location:** country: United States; stateProvince: Mississippi; county: Bolivar; locality: University Street, Cleveland; decimalLatitude: 33.73521; decimalLongitude: -90.73244; geodeticDatum: WGS1984; **Identification:** identifiedBy: K. A. Parys; dateIdentified: 2017; **Event:** samplingProtocol: netting; eventDate: 2017-6-17; **Record Level:** type: PhysicalObject; language: en; rights: https://creativecommons.org/publicdomain/zero/1.0/; accessRights: http://vertnet.org/resources/norms.html; institutionCode: YUTO; collectionCode: PCYU; basisOfRecord: PreservedSpecimen
- **Type status:** Other material. **Occurrence:** catalogNumber: SIMRU15316; recordedBy: K. A. Parys; individualCount: 1; sex: male; lifeStage: adult; preparations: pin; occurrenceID: urn:YUTO:PCYU:SIMRU15316; **Taxon:** scientificName: Anthemurgus passiflorae Robertson, 1902; kingdom: Animalia; phylum: Arthropoda; class: Insecta; order: Hymenoptera; family: Andrenidae; genus: Anthemurgus; specificEpithet: passiflorae; scientificNameAuthorship: Robertson, 1902; **Location:** country: United States; stateProvince: Mississippi; county: Bolivar; locality: University Street, Cleveland; decimalLatitude: 33.73521; decimalLongitude: -90.73244; geodeticDatum: WGS1984; **Identification:** identifiedBy: K. A. Parys; dateIdentified: 2017; **Event:** samplingProtocol: netting; eventDate: 2017-6-17; **Record Level:** type: PhysicalObject; language: en; rights: https://creativecommons.org/publicdomain/zero/1.0/; accessRights: http://vertnet.org/resources/norms.html; institutionCode: YUTO; collectionCode: PCYU; basisOfRecord: PreservedSpecimen
- **Type status:** Other material. **Occurrence:** catalogNumber: SIMRU15317; recordedBy: K. A. Parys; individualCount: 1; sex: female; lifeStage: adult; preparations: pin; occurrenceID: urn:USDA-ARS:SIMRU:SIMRU15317; **Taxon:** scientificName: Anthemurgus passiflorae Robertson, 1902; kingdom: Animalia; phylum: Arthropoda; class: Insecta; order: Hymenoptera; family: Andrenidae; genus: Anthemurgus; specificEpithet: passiflorae; scientificNameAuthorship: Robertson, 1902; **Location:** country: United States; stateProvince: Mississippi; county: Bolivar; locality: University Street, Cleveland; decimalLatitude: 33.73521; decimalLongitude: -90.73244; geodeticDatum: WGS1984; **Identification:** identifiedBy: K. A. Parys; dateIdentified: 2017; **Event:** samplingProtocol: netting; eventDate: 2017-6-17; **Record Level:** type: PhysicalObject; language: en; rights: https://creativecommons.org/publicdomain/zero/1.0/; accessRights: http://vertnet.org/resources/norms.html; institutionCode: USDA-ARS; collectionCode: SIMRU; basisOfRecord: PreservedSpecimen
- **Type status:** Other material. **Occurrence:** catalogNumber: SIMRU17497; recordedBy: K. A. Parys; individualCount: 1; sex: female; lifeStage: adult; preparations: pin; occurrenceID: urn:USDA-ARS:SIMRU:SIMRU17497; **Taxon:** scientificName: Anthemurgus passiflorae Robertson, 1902; kingdom: Animalia; phylum: Arthropoda; class: Insecta; order: Hymenoptera; family: Andrenidae; genus: Anthemurgus; specificEpithet: passiflorae; scientificNameAuthorship: Robertson, 1902; **Location:** country: United States; stateProvince: Mississippi; county: Bolivar; locality: University Street, Cleveland; decimalLatitude: 33.73521; decimalLongitude: -90.73244; geodeticDatum: WGS1984; **Identification:** identifiedBy: K. A. Parys; dateIdentified: 2017; **Event:** samplingProtocol: netting; eventDate: 2017-7-9; **Record Level:** type: PhysicalObject; language: en; rights: https://creativecommons.org/publicdomain/zero/1.0/; accessRights: http://vertnet.org/resources/norms.html; institutionCode: USDA-ARS; collectionCode: SIMRU; basisOfRecord: PreservedSpecimen
- **Type status:** Other material. **Occurrence:** catalogNumber: SIMRU17498; recordedBy: K. A. Parys; individualCount: 1; sex: female; lifeStage: adult; preparations: pin; occurrenceID: urn:USDA-ARS:SIMRU:SIMRU17498; **Taxon:** scientificName: Anthemurgus passiflorae Robertson, 1902; kingdom: Animalia; phylum: Arthropoda; class: Insecta; order: Hymenoptera; family: Andrenidae; genus: Anthemurgus; specificEpithet: passiflorae; scientificNameAuthorship: Robertson, 1902; **Location:** country: United States; stateProvince: Mississippi; county: Bolivar; locality: University Street, Cleveland; decimalLatitude: 33.73521; decimalLongitude: -90.73244; geodeticDatum: WGS1984; **Identification:** identifiedBy: K. A. Parys; dateIdentified: 2017; **Event:** samplingProtocol: netting; eventDate: 2017-7-9; **Record Level:** type: PhysicalObject; language: en; rights: https://creativecommons.org/publicdomain/zero/1.0/; accessRights: http://vertnet.org/resources/norms.html; institutionCode: USDA-ARS; collectionCode: SIMRU; basisOfRecord: PreservedSpecimen
- **Type status:** Other material. **Occurrence:** catalogNumber: SIMRU17499; recordedBy: K. A. Parys; individualCount: 1; sex: male; lifeStage: adult; preparations: pin; occurrenceID: urn:USDA-ARS:SIMRU:SIMRU17499; **Taxon:** scientificName: Anthemurgus passiflorae Robertson, 1902; kingdom: Animalia; phylum: Arthropoda; class: Insecta; order: Hymenoptera; family: Andrenidae; genus: Anthemurgus; specificEpithet: passiflorae; scientificNameAuthorship: Robertson, 1902; **Location:** country: United States; stateProvince: Mississippi; county: Bolivar; locality: University Street, Cleveland; decimalLatitude: 33.73521; decimalLongitude: -90.73244; geodeticDatum: WGS1984; **Identification:** identifiedBy: K. A. Parys; dateIdentified: 2017; **Event:** samplingProtocol: netting; eventDate: 2017-7-9; **Record Level:** type: PhysicalObject; language: en; rights: https://creativecommons.org/publicdomain/zero/1.0/; accessRights: http://vertnet.org/resources/norms.html; institutionCode: USDA-ARS; collectionCode: SIMRU; basisOfRecord: PreservedSpecimen
- **Type status:** Other material. **Occurrence:** catalogNumber: SIMRU17500; recordedBy: K. A. Parys; individualCount: 1; sex: female; lifeStage: adult; preparations: pin; occurrenceID: urn:USDA-ARS:SIMRU:SIMRU17500; **Taxon:** scientificName: Anthemurgus passiflorae Robertson, 1902; kingdom: Animalia; phylum: Arthropoda; class: Insecta; order: Hymenoptera; family: Andrenidae; genus: Anthemurgus; specificEpithet: passiflorae; scientificNameAuthorship: Robertson, 1902; **Location:** country: United States; stateProvince: Mississippi; county: Bolivar; locality: University Street, Cleveland; decimalLatitude: 33.73521; decimalLongitude: -90.73244; geodeticDatum: WGS1984; **Identification:** identifiedBy: K. A. Parys; dateIdentified: 2017; **Event:** samplingProtocol: netting; eventDate: 2017-7-9; **Record Level:** type: PhysicalObject; language: en; rights: https://creativecommons.org/publicdomain/zero/1.0/; accessRights: http://vertnet.org/resources/norms.html; institutionCode: USDA-ARS; collectionCode: SIMRU; basisOfRecord: PreservedSpecimen
- **Type status:** Other material. **Occurrence:** catalogNumber: SIMRU17501; recordedBy: K. A. Parys; individualCount: 1; sex: female; lifeStage: adult; preparations: pin; occurrenceID: urn:USDA-ARS:SIMRU:SIMRU17501; **Taxon:** scientificName: Anthemurgus passiflorae Robertson, 1902; kingdom: Animalia; phylum: Arthropoda; class: Insecta; order: Hymenoptera; family: Andrenidae; genus: Anthemurgus; specificEpithet: passiflorae; scientificNameAuthorship: Robertson, 1902; **Location:** country: United States; stateProvince: Mississippi; county: Bolivar; locality: University Street, Cleveland; decimalLatitude: 33.73521; decimalLongitude: -90.73244; geodeticDatum: WGS1984; **Identification:** identifiedBy: K. A. Parys; dateIdentified: 2017; **Event:** samplingProtocol: netting; eventDate: 2017-7-9; **Record Level:** type: PhysicalObject; language: en; rights: https://creativecommons.org/publicdomain/zero/1.0/; accessRights: http://vertnet.org/resources/norms.html; institutionCode: USDA-ARS; collectionCode: SIMRU; basisOfRecord: PreservedSpecimen
- **Type status:** Other material. **Occurrence:** catalogNumber: SIMRU17502; recordedBy: K. A. Parys; individualCount: 1; sex: female; lifeStage: adult; preparations: pin; occurrenceID: urn:USDA-ARS:SIMRU:SIMRU17502; **Taxon:** scientificName: Anthemurgus passiflorae Robertson, 1902; kingdom: Animalia; phylum: Arthropoda; class: Insecta; order: Hymenoptera; family: Andrenidae; genus: Anthemurgus; specificEpithet: passiflorae; scientificNameAuthorship: Robertson, 1902; **Location:** country: United States; stateProvince: Mississippi; county: Bolivar; locality: University Street, Cleveland; decimalLatitude: 33.73521; decimalLongitude: -90.73244; geodeticDatum: WGS1984; **Identification:** identifiedBy: K. A. Parys; dateIdentified: 2017; **Event:** samplingProtocol: netting; eventDate: 2017-7-9; **Record Level:** type: PhysicalObject; language: en; rights: https://creativecommons.org/publicdomain/zero/1.0/; accessRights: http://vertnet.org/resources/norms.html; institutionCode: USDA-ARS; collectionCode: SIMRU; basisOfRecord: PreservedSpecimen
- **Type status:** Other material. **Occurrence:** catalogNumber: SIMRU17503; recordedBy: K. A. Parys; individualCount: 1; sex: male; lifeStage: adult; preparations: pin; occurrenceID: urn:USDA-ARS:SIMRU:SIMRU17503; **Taxon:** scientificName: Anthemurgus passiflorae Robertson, 1902; kingdom: Animalia; phylum: Arthropoda; class: Insecta; order: Hymenoptera; family: Andrenidae; genus: Anthemurgus; specificEpithet: passiflorae; scientificNameAuthorship: Robertson, 1902; **Location:** country: United States; stateProvince: Mississippi; county: Bolivar; locality: University Street, Cleveland; decimalLatitude: 33.73521; decimalLongitude: -90.73244; geodeticDatum: WGS1984; **Identification:** identifiedBy: K. A. Parys; dateIdentified: 2017; **Event:** samplingProtocol: netting; eventDate: 2017-7-9; **Record Level:** type: PhysicalObject; language: en; rights: https://creativecommons.org/publicdomain/zero/1.0/; accessRights: http://vertnet.org/resources/norms.html; institutionCode: USDA-ARS; collectionCode: SIMRU; basisOfRecord: PreservedSpecimen
- **Type status:** Other material. **Occurrence:** catalogNumber: SIMRU17504; recordedBy: K. A. Parys; individualCount: 1; sex: male; lifeStage: adult; preparations: pin; occurrenceID: urn:USDA-ARS:SIMRU:SIMRU17504; **Taxon:** scientificName: Anthemurgus passiflorae Robertson, 1902; kingdom: Animalia; phylum: Arthropoda; class: Insecta; order: Hymenoptera; family: Andrenidae; genus: Anthemurgus; specificEpithet: passiflorae; scientificNameAuthorship: Robertson, 1902; **Location:** country: United States; stateProvince: Mississippi; county: Bolivar; locality: University Street, Cleveland; decimalLatitude: 33.73521; decimalLongitude: -90.73244; geodeticDatum: WGS1984; **Identification:** identifiedBy: K. A. Parys; dateIdentified: 2017; **Event:** samplingProtocol: netting; eventDate: 2017-7-9; **Record Level:** type: PhysicalObject; language: en; rights: https://creativecommons.org/publicdomain/zero/1.0/; accessRights: http://vertnet.org/resources/norms.html; institutionCode: USDA-ARS; collectionCode: SIMRU; basisOfRecord: PreservedSpecimen
- **Type status:** Other material. **Occurrence:** catalogNumber: SIMRU17933; recordedBy: K. A. Parys; individualCount: 1; sex: male; lifeStage: adult; preparations: pin; occurrenceID: urn:USDA-ARS:SIMRU:SIMRU17933; **Taxon:** scientificName: Anthemurgus passiflorae Robertson, 1902; kingdom: Animalia; phylum: Arthropoda; class: Insecta; order: Hymenoptera; family: Andrenidae; genus: Anthemurgus; specificEpithet: passiflorae; scientificNameAuthorship: Robertson, 1902; **Location:** country: United States; stateProvince: Mississippi; county: Bolivar; locality: University Street, Cleveland; decimalLatitude: 33.73521; decimalLongitude: -90.73244; geodeticDatum: WGS1984; **Identification:** identifiedBy: T. Griswold; dateIdentified: 2017; **Event:** samplingProtocol: netting; eventDate: 2017-7-4; **Record Level:** type: PhysicalObject; language: en; rights: https://creativecommons.org/publicdomain/zero/1.0/; accessRights: http://vertnet.org/resources/norms.html; institutionCode: USDA-ARS; collectionCode: SIMRU; basisOfRecord: PreservedSpecimen
- **Type status:** Other material. **Occurrence:** catalogNumber: SIMRU17934; recordedBy: K. A. Parys; individualCount: 1; sex: male; lifeStage: adult; preparations: pin; occurrenceID: urn:USDA-ARS:SIMRU:SIMRU17934; **Taxon:** scientificName: Anthemurgus passiflorae Robertson, 1902; kingdom: Animalia; phylum: Arthropoda; class: Insecta; order: Hymenoptera; family: Andrenidae; genus: Anthemurgus; specificEpithet: passiflorae; scientificNameAuthorship: Robertson, 1902; **Location:** country: United States; stateProvince: Mississippi; county: Bolivar; locality: University Street, Cleveland; decimalLatitude: 33.73521; decimalLongitude: -90.73244; geodeticDatum: WGS1984; **Identification:** identifiedBy: T. Griswold; dateIdentified: 2017; **Event:** samplingProtocol: netting; eventDate: 2017-7-4; **Record Level:** type: PhysicalObject; language: en; rights: https://creativecommons.org/publicdomain/zero/1.0/; accessRights: http://vertnet.org/resources/norms.html; institutionCode: USDA-ARS; collectionCode: SIMRU; basisOfRecord: PreservedSpecimen
- **Type status:** Other material. **Occurrence:** catalogNumber: SIMRU17935; recordedBy: K. A. Parys; individualCount: 1; sex: male; lifeStage: adult; preparations: pin; occurrenceID: urn:USDA-ARS:SIMRU:SIMRU17935; **Taxon:** scientificName: Anthemurgus passiflorae Robertson, 1902; kingdom: Animalia; phylum: Arthropoda; class: Insecta; order: Hymenoptera; family: Andrenidae; genus: Anthemurgus; specificEpithet: passiflorae; scientificNameAuthorship: Robertson, 1902; **Location:** country: United States; stateProvince: Mississippi; county: Bolivar; locality: University Street, Cleveland; decimalLatitude: 33.73521; decimalLongitude: -90.73244; geodeticDatum: WGS1984; **Identification:** identifiedBy: T. Griswold; dateIdentified: 2017; **Event:** samplingProtocol: netting; eventDate: 2017-7-4; **Record Level:** type: PhysicalObject; language: en; rights: https://creativecommons.org/publicdomain/zero/1.0/; accessRights: http://vertnet.org/resources/norms.html; institutionCode: USDA-ARS; collectionCode: SIMRU; basisOfRecord: PreservedSpecimen
- **Type status:** Other material. **Occurrence:** catalogNumber: SIMRU20858; recordedBy: K. A. Parys; individualCount: 1; sex: male; lifeStage: adult; preparations: pin; occurrenceID: urn:USDA-ARS:SIMRU:SIMRU20858; **Taxon:** scientificName: Anthemurgus passiflorae Robertson, 1902; kingdom: Animalia; phylum: Arthropoda; class: Insecta; order: Hymenoptera; family: Andrenidae; genus: Anthemurgus; specificEpithet: passiflorae; scientificNameAuthorship: Robertson, 1902; **Location:** country: United States; stateProvince: Mississippi; county: Bolivar; locality: University Street, Cleveland; decimalLatitude: 33.73521; decimalLongitude: -90.73244; geodeticDatum: WGS1984; **Identification:** identifiedBy: T. Griswold; dateIdentified: 2017; **Event:** samplingProtocol: netting; eventDate: 2017-7-31; **Record Level:** type: PhysicalObject; language: en; rights: https://creativecommons.org/publicdomain/zero/1.0/; accessRights: http://vertnet.org/resources/norms.html; institutionCode: USDA-ARS; collectionCode: SIMRU; basisOfRecord: PreservedSpecimen
- **Type status:** Other material. **Occurrence:** catalogNumber: SIMRU20859; recordedBy: K. A. Parys; individualCount: 1; sex: male; lifeStage: adult; preparations: pin; occurrenceID: urn:USDA-ARS:SIMRU:SIMRU20859; **Taxon:** scientificName: Anthemurgus passiflorae Robertson, 1902; kingdom: Animalia; phylum: Arthropoda; class: Insecta; order: Hymenoptera; family: Andrenidae; genus: Anthemurgus; specificEpithet: passiflorae; scientificNameAuthorship: Robertson, 1902; **Location:** country: United States; stateProvince: Mississippi; county: Bolivar; locality: University Street, Cleveland; decimalLatitude: 33.73521; decimalLongitude: -90.73244; geodeticDatum: WGS1984; **Identification:** identifiedBy: T. Griswold; dateIdentified: 2017; **Event:** samplingProtocol: netting; eventDate: 2017-7-31; **Record Level:** type: PhysicalObject; language: en; rights: https://creativecommons.org/publicdomain/zero/1.0/; accessRights: http://vertnet.org/resources/norms.html; institutionCode: USDA-ARS; collectionCode: SIMRU; basisOfRecord: PreservedSpecimen
- **Type status:** Other material. **Occurrence:** catalogNumber: SIMRU20860; recordedBy: K. A. Parys; individualCount: 1; sex: male; lifeStage: adult; preparations: pin; occurrenceID: urn:USDA-ARS:SIMRU:SIMRU20860; **Taxon:** scientificName: Anthemurgus passiflorae Robertson, 1902; kingdom: Animalia; phylum: Arthropoda; class: Insecta; order: Hymenoptera; family: Andrenidae; genus: Anthemurgus; specificEpithet: passiflorae; scientificNameAuthorship: Robertson, 1902; **Location:** country: United States; stateProvince: Mississippi; county: Bolivar; locality: University Street, Cleveland; decimalLatitude: 33.73521; decimalLongitude: -90.73244; geodeticDatum: WGS1984; **Identification:** identifiedBy: T. Griswold; dateIdentified: 2017; **Event:** samplingProtocol: netting; eventDate: 2017-7-31; **Record Level:** type: PhysicalObject; language: en; rights: https://creativecommons.org/publicdomain/zero/1.0/; accessRights: http://vertnet.org/resources/norms.html; institutionCode: USDA-ARS; collectionCode: SIMRU; basisOfRecord: PreservedSpecimen

##### Notes

**Figure 2.**
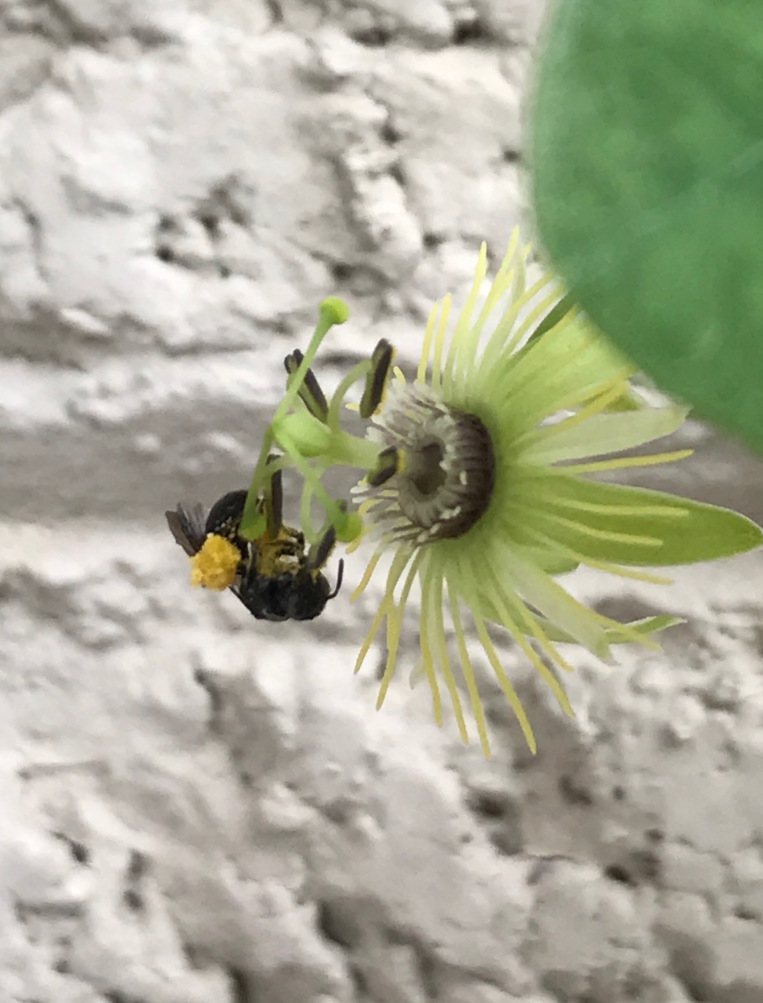
Female *A. passiflorae* observed on *P. lutea* in Bolivar County, Mississippi

This species is monotypic and oligolectic on a native passionflower, *Passiflora lutea* L. (Fig. 2). Neff and Rozen (1995) described foraging behaviour and larval characteristics. This species' known range is from central Texas, Kansas, Illinois and east to North Carolina in the United States (Michener et al. 1994, Neff and Rozen 1995, GBIF 2017b); this is the first report of this species in Mississippi. All 23 specimens of *A. passiflorae* reported here were net collected from *P. lutea* growing at a single location in Cleveland, MS, located in Bolivar County (Fig. 3).

#### Halictidae

#### Dieunomia (Dieunomia) bolliana

(Cockerell, 1910)

##### Materials

- **Type status:** Other material. **Occurrence:** catalogNumber: SIMRU5326; recordedBy: K. A. Parys; individualCount: 1; sex: female; lifeStage: adult; preparations: pin; occurrenceID: urn:USDA-ARS:SIMRU:SIMRU5326; **Taxon:** scientificName: Dieunomia (Dieunomia) bolliana (Cockerell, 1910); kingdom: Animalia; phylum: Arthropoda; class: Insecta; order: Hymenoptera; family: Halictidae; genus: Dieunomia; subgenus: Dieunomia; specificEpithet: bolliana; scientificNameAuthorship: (Cockerell, 1910); **Location:** country: United States; stateProvince: Mississippi; county: Sunflower; locality: Indianola; decimalLatitude: 33.451759; decimalLongitude: -90.688321; geodeticDatum: WGS1984; **Identification:** identifiedBy: K. A. Parys; dateIdentified: 2017; **Event:** samplingProtocol: netting; eventDate: 2016-6-13; **Record Level:** type: PhysicalObject; language: en; rights: https://creativecommons.org/publicdomain/zero/1.0/; accessRights: http://vertnet.org/resources/norms.html; institutionCode: USDA-ARS; collectionCode: SIMRU; basisOfRecord: PreservedSpecimen
- **Type status:** Other material. **Occurrence:** catalogNumber: SIMRU5338; recordedBy: K. A. Parys; individualCount: 1; sex: female; lifeStage: adult; preparations: pin; occurrenceID: urn:USDA-ARS:SIMRU:SIMRU5338; **Taxon:** scientificName: Dieunomia (Dieunomia) bolliana (Cockerell, 1910); kingdom: Animalia; phylum: Arthropoda; class: Insecta; order: Hymenoptera; family: Halictidae; genus: Dieunomia; subgenus: Dieunomia; specificEpithet: bolliana; scientificNameAuthorship: (Cockerell, 1910); **Location:** country: United States; stateProvince: Mississippi; county: Sunflower; locality: Ruleville; decimalLatitude: 33.792135; decimalLongitude: -90.646879; geodeticDatum: WGS1984; **Identification:** identifiedBy: K. A. Parys; dateIdentified: 2017; **Event:** samplingProtocol: sweeping; eventDate: 2016-6-10; **Record Level:** type: PhysicalObject; language: en; rights: https://creativecommons.org/publicdomain/zero/1.0/; accessRights: http://vertnet.org/resources/norms.html; institutionCode: USDA-ARS; collectionCode: SIMRU; basisOfRecord: PreservedSpecimen
- **Type status:** Other material. **Occurrence:** catalogNumber: SIMRU12621; recordedBy: K. A. Parys; individualCount: 1; sex: male; lifeStage: adult; preparations: pin; occurrenceID: urn:USDA-ARS:SIMRU:SIMRU12621; **Taxon:** scientificName: Dieunomia (Dieunomia) bolliana (Cockerell, 1910); kingdom: Animalia; phylum: Arthropoda; class: Insecta; order: Hymenoptera; family: Halictidae; genus: Dieunomia; subgenus: Dieunomia; specificEpithet: bolliana; scientificNameAuthorship: (Cockerell, 1910); **Location:** country: United States; stateProvince: Mississippi; county: Sunflower; locality: Indianola; decimalLatitude: 33.46764; decimalLongitude: -90.63178; geodeticDatum: WGS1984; **Identification:** identifiedBy: M. C. Orr; dateIdentified: 2017; **Event:** samplingProtocol: sweeping; eventDate: 2016-6-28; **Record Level:** type: PhysicalObject; language: en; rights: https://creativecommons.org/publicdomain/zero/1.0/; accessRights: http://vertnet.org/resources/norms.html; institutionCode: USDA-ARS; collectionCode: SIMRU; basisOfRecord: PreservedSpecimen
- **Type status:** Other material. **Occurrence:** catalogNumber: SIMRU14138; recordedBy: K. A. Parys; individualCount: 1; sex: male; lifeStage: adult; preparations: pin; occurrenceID: urn:USDA-ARS:BBSL:SIMRU14138; **Taxon:** scientificName: Dieunomia (Dieunomia) bolliana (Cockerell, 1910); kingdom: Animalia; phylum: Arthropoda; class: Insecta; order: Hymenoptera; family: Halictidae; genus: Dieunomia; subgenus: Dieunomia; specificEpithet: bolliana; scientificNameAuthorship: (Cockerell, 1910); **Location:** country: United States; stateProvince: Mississippi; county: Bolivar; locality: Old Hwy 61; decimalLatitude: 33.821; decimalLongitude: -90.7252; geodeticDatum: WGS1984; **Identification:** identifiedBy: M. C. Orr; dateIdentified: 2017; **Event:** samplingProtocol: netting; eventDate: 2017-6-9; **Record Level:** type: PhysicalObject; language: en; rights: https://creativecommons.org/publicdomain/zero/1.0/; accessRights: http://vertnet.org/resources/norms.html; institutionCode: USDA-ARS; collectionCode: BBSL; basisOfRecord: PreservedSpecimen
- **Type status:** Other material. **Occurrence:** catalogNumber: SIMRU14141; recordedBy: K. A. Parys; individualCount: 1; sex: male; lifeStage: adult; preparations: pin; occurrenceID: urn:USDA-ARS:SIMRU:SIMRU14141; **Taxon:** scientificName: Dieunomia (Dieunomia) bolliana (Cockerell, 1910); kingdom: Animalia; phylum: Arthropoda; class: Insecta; order: Hymenoptera; family: Halictidae; genus: Dieunomia; subgenus: Dieunomia; specificEpithet: bolliana; scientificNameAuthorship: (Cockerell, 1910); **Location:** country: United States; stateProvince: Mississippi; county: Bolivar; locality: Old Hwy 61; decimalLatitude: 33.821; decimalLongitude: -90.7252; geodeticDatum: WGS1984; **Identification:** identifiedBy: M. C. Orr; dateIdentified: 2017; **Event:** samplingProtocol: netting; eventDate: 2017-6-9; **Record Level:** type: PhysicalObject; language: en; rights: https://creativecommons.org/publicdomain/zero/1.0/; accessRights: http://vertnet.org/resources/norms.html; institutionCode: USDA-ARS; collectionCode: SIMRU; basisOfRecord: PreservedSpecimen
- **Type status:** Other material. **Occurrence:** catalogNumber: SIMRU14147; recordedBy: K. A. Parys; individualCount: 1; sex: female; lifeStage: adult; preparations: pin; occurrenceID: urn:USDA-ARS:BBSL:SIMRU14147; **Taxon:** scientificName: Dieunomia (Dieunomia) bolliana (Cockerell, 1910); kingdom: Animalia; phylum: Arthropoda; class: Insecta; order: Hymenoptera; family: Halictidae; genus: Dieunomia; subgenus: Dieunomia; specificEpithet: bolliana; scientificNameAuthorship: (Cockerell, 1910); **Location:** country: United States; stateProvince: Mississippi; county: Bolivar; locality: Old Hwy 61; decimalLatitude: 33.821; decimalLongitude: -90.7252; geodeticDatum: WGS1984; **Identification:** identifiedBy: M. C. Orr; dateIdentified: 2017; **Event:** samplingProtocol: netting; eventDate: 2017-6-9; **Record Level:** type: PhysicalObject; language: en; rights: https://creativecommons.org/publicdomain/zero/1.0/; accessRights: http://vertnet.org/resources/norms.html; institutionCode: USDA-ARS; collectionCode: BBSL; basisOfRecord: PreservedSpecimen
- **Type status:** Other material. **Occurrence:** catalogNumber: SIMRU18906; recordedBy: K. A. Parys; individualCount: 1; sex: male; lifeStage: adult; preparations: pin; occurrenceID: urn:USDA-ARS:SIMRU:SIMRU18906; **Taxon:** scientificName: Dieunomia (Dieunomia) bolliana (Cockerell, 1910); kingdom: Animalia; phylum: Arthropoda; class: Insecta; order: Hymenoptera; family: Halictidae; genus: Dieunomia; subgenus: Dieunomia; specificEpithet: bolliana; scientificNameAuthorship: (Cockerell, 1910); **Location:** country: United States; stateProvince: Mississippi; county: Bolivar; locality: Alcorn Research Farm, Mound Bayou; decimalLatitude: 33.871265; decimalLongitude: -90.699184; geodeticDatum: WGS1984; **Identification:** identifiedBy: M. C. Orr; dateIdentified: 2017; **Event:** samplingProtocol: Vane Trap (Blue); eventDate: 2017-7-25; **Record Level:** type: PhysicalObject; language: en; rights: https://creativecommons.org/publicdomain/zero/1.0/; accessRights: http://vertnet.org/resources/norms.html; institutionCode: USDA-ARS; collectionCode: SIMRU; basisOfRecord: PreservedSpecimen
- **Type status:** Other material. **Occurrence:** catalogNumber: SIMRU19113; recordedBy: K. A. Parys; individualCount: 1; sex: male; lifeStage: adult; preparations: pin; occurrenceID: urn:USDA-ARS:SIMRU:SIMRU19113; **Taxon:** scientificName: Dieunomia (Dieunomia) bolliana (Cockerell, 1910); kingdom: Animalia; phylum: Arthropoda; class: Insecta; order: Hymenoptera; family: Halictidae; genus: Dieunomia; subgenus: Dieunomia; specificEpithet: bolliana; scientificNameAuthorship: (Cockerell, 1910); **Location:** country: United States; stateProvince: Mississippi; county: Washington; locality: SIMRU grp 4/5 soybeans, 1pm; decimalLatitude: 33.34533; decimalLongitude: -90.91978; geodeticDatum: WGS1984; **Identification:** identifiedBy: M. C. Orr; dateIdentified: 2017; **Event:** samplingProtocol: Bee Bowl (Blue); eventDate: 2017-6-21; **Record Level:** type: PhysicalObject; language: en; rights: https://creativecommons.org/publicdomain/zero/1.0/; accessRights: http://vertnet.org/resources/norms.html; institutionCode: USDA-ARS; collectionCode: SIMRU; basisOfRecord: PreservedSpecimen
- **Type status:** Other material. **Occurrence:** catalogNumber: SIMRU19116; recordedBy: K. A. Parys; individualCount: 1; sex: male; lifeStage: adult; preparations: pin; occurrenceID: urn:USDA-ARS:SIMRU:SIMRU19116; **Taxon:** scientificName: Dieunomia (Dieunomia) bolliana (Cockerell, 1910); kingdom: Animalia; phylum: Arthropoda; class: Insecta; order: Hymenoptera; family: Halictidae; genus: Dieunomia; subgenus: Dieunomia; specificEpithet: bolliana; scientificNameAuthorship: (Cockerell, 1910); **Location:** country: United States; stateProvince: Mississippi; county: Washington; locality: SIMRU grp 4/5 soybeans, 1pm; decimalLatitude: 33.34533; decimalLongitude: -90.91978; geodeticDatum: WGS1984; **Identification:** identifiedBy: M. C. Orr; dateIdentified: 2017; **Event:** samplingProtocol: Bee Bowl (Blue); eventDate: 2017-6-21; **Record Level:** type: PhysicalObject; language: en; rights: https://creativecommons.org/publicdomain/zero/1.0/; accessRights: http://vertnet.org/resources/norms.html; institutionCode: USDA-ARS; collectionCode: SIMRU; basisOfRecord: PreservedSpecimen
- **Type status:** Other material. **Occurrence:** catalogNumber: SIMRU22173; recordedBy: K. A. Parys; individualCount: 1; sex: male; lifeStage: adult; preparations: pin; occurrenceID: urn:USDA-ARS:SIMRU:SIMRU22173; **Taxon:** scientificName: Dieunomia (Dieunomia) bolliana (Cockerell, 1910); kingdom: Animalia; phylum: Arthropoda; class: Insecta; order: Hymenoptera; family: Halictidae; genus: Dieunomia; subgenus: Dieunomia; specificEpithet: bolliana; scientificNameAuthorship: (Cockerell, 1910); **Location:** country: United States; stateProvince: Mississippi; county: Sunflower; locality: Indianola; decimalLatitude: 33.46764; decimalLongitude: -90.63178; geodeticDatum: WGS1984; **Identification:** identifiedBy: M. C. Orr; dateIdentified: 2017; **Event:** samplingProtocol: sweeping; eventDate: 2016-6-28; **Record Level:** type: PhysicalObject; language: en; rights: https://creativecommons.org/publicdomain/zero/1.0/; accessRights: http://vertnet.org/resources/norms.html; institutionCode: USDA-ARS; collectionCode: SIMRU; basisOfRecord: PreservedSpecimen

##### Notes

Originally described from Texas (Cockerell 1910), this species is smaller than *Dieunomia heteropoda* (Say), the more commonly encountered species in northern Mississippi. Two subspecies are currently recognised, the second of which is D. (D.) bolliana helenii (Cockerell 1936). We choose not to use a subspecific classification here, given their questionable status. Notably, Cockerell himself admits that a specimen collected alongside the type of his subspecies "approaches the typical form in having the mesothorax and sides of thorax black" (Cockerell 1936). Similar variation in other collections of this species and other *Dieunomia* has been observed by MCO. Ultimately, it seems unlikely that this subspecific epithet will survive subsequent taxonomic treatment.

The currently known distribution is reported only for the south-western United States of Texas and New Mexico and ranges south into México (Hurd et al. 1980). Cockerell (1910) reported collections made from *Dracopis amplexicaulis* (Vahl) Cass (listed as *Rudbeckia amplexicaulis*) and *Helianthus* sp. Additional observations of *D. bolliana* individuals collecting pollen and nectar were reported from *Helenium microcephalum* DC. and *Polypteris texana* (DC) A. Gray (Cockerell 1936). Hurd et al. (1980) regard this species as a oligolege of composites, secondarily associated with *Helianthus*.

Of the ten specimens of *D. bolliana* reported here from Mississippi (Fig. 4), nine were collected in the month of June and three of those were collected by sweeping roadside patches of *Coreopsis* sp. for other insects. Two specimens were collected in soybeans on the USDA ARS' research farm outside Leland, MS. The remaining four June specimens were also collected from roadsides by sweeping, but no host plant was recorded. The last specimen was collected in a blue vane trap during July at the Alcorn State University Research Farm located in Mound Bayou, MS.

#### Apidae

#### Brachynomadini

#### Brachynomada (Melanomada) nimia

(Snelling & Rozen, 1987)

##### Materials

- **Type status:** Other material. **Occurrence:** catalogNumber: SIMRU10814; recordedBy: K. A. Parys; individualCount: 1; sex: male; lifeStage: adult; preparations: pin; occurrenceID: urn:USDA-ARS:BBSL:SIMRU10814; **Taxon:** scientificName: Brachynomada (Melanomada) nimia (Snelling and Rozen, 1987); kingdom: Animalia; phylum: Arthropoda; class: Insecta; order: Hymenoptera; family: Apidae; genus: Brachynomada; subgenus: Melanomada; specificEpithet: nimia; scientificNameAuthorship: (Snelling and Rozen, 1987); **Location:** country: United States; stateProvince: Mississippi; county: Tallahatchie; locality: Tallahatchie National Wildlife Refuge; decimalLatitude: 33.757902; decimalLongitude: -90.149618; geodeticDatum: WGS1984; **Identification:** identifiedBy: T. Griswold; dateIdentified: 2017; **Event:** samplingProtocol: malaise; eventDate: 2016-9-23; **Record Level:** type: PhysicalObject; language: en; rights: https://creativecommons.org/publicdomain/zero/1.0/; accessRights: http://vertnet.org/resources/norms.html; institutionCode: USDA-ARS; collectionCode: BBSL; basisOfRecord: PreservedSpecimen
- **Type status:** Other material. **Occurrence:** catalogNumber: SIMRU10882; recordedBy: K. A. Parys; individualCount: 1; sex: male; lifeStage: adult; preparations: pin; occurrenceID: urn:USDA-ARS:SIMRU:SIMRU10882; **Taxon:** scientificName: Brachynomada (Melanomada) nimia (Snelling and Rozen, 1987); kingdom: Animalia; phylum: Arthropoda; class: Insecta; order: Hymenoptera; family: Apidae; genus: Brachynomada; subgenus: Melanomada; specificEpithet: nimia; scientificNameAuthorship: (Snelling and Rozen, 1987); **Location:** country: United States; stateProvince: Mississippi; county: Tallahatchie; locality: Tallahatchie National Wildlife Refuge; decimalLatitude: 33.760588; decimalLongitude: -90.150779; geodeticDatum: WGS1984; **Identification:** identifiedBy: T. Griswold; dateIdentified: 2017; **Event:** samplingProtocol: malaise; eventDate: 2016-9-28; **Record Level:** type: PhysicalObject; language: en; rights: https://creativecommons.org/publicdomain/zero/1.0/; accessRights: http://vertnet.org/resources/norms.html; institutionCode: USDA-ARS; collectionCode: SIMRU; basisOfRecord: PreservedSpecimen
- **Type status:** Other material. **Occurrence:** catalogNumber: SIMRU10883; recordedBy: K. A. Parys; individualCount: 1; sex: male; lifeStage: adult; preparations: pin; occurrenceID: urn:USDA-ARS:BBSL:SIMRU10883; **Taxon:** scientificName: Brachynomada (Melanomada) nimia (Snelling and Rozen, 1987); kingdom: Animalia; phylum: Arthropoda; class: Insecta; order: Hymenoptera; family: Apidae; genus: Brachynomada; subgenus: Melanomada; specificEpithet: nimia; scientificNameAuthorship: (Snelling and Rozen, 1987); **Location:** country: United States; stateProvince: Mississippi; county: Tallahatchie; locality: Tallahatchie National Wildlife Refuge; decimalLatitude: 33.760588; decimalLongitude: -90.150779; geodeticDatum: WGS1984; **Identification:** identifiedBy: T. Griswold; dateIdentified: 2017; **Event:** samplingProtocol: malaise; eventDate: 2016-9-28; **Record Level:** type: PhysicalObject; language: en; rights: https://creativecommons.org/publicdomain/zero/1.0/; accessRights: http://vertnet.org/resources/norms.html; institutionCode: USDA-ARS; collectionCode: BBSL; basisOfRecord: PreservedSpecimen
- **Type status:** Other material. **Occurrence:** catalogNumber: SIMRU10909; recordedBy: K. A. Parys; individualCount: 1; sex: male; lifeStage: adult; preparations: pin; occurrenceID: urn:USDA-ARS:BBSL:SIMRU10909; **Taxon:** scientificName: Brachynomada (Melanomada) nimia (Snelling and Rozen, 1987); kingdom: Animalia; phylum: Arthropoda; class: Insecta; order: Hymenoptera; family: Apidae; genus: Brachynomada; subgenus: Melanomada; specificEpithet: nimia; scientificNameAuthorship: (Snelling and Rozen, 1987); **Location:** country: United States; stateProvince: Mississippi; county: Tallahatchie; locality: Tallahatchie National Wildlife Refuge; decimalLatitude: 33.760588; decimalLongitude: -90.150779; geodeticDatum: WGS1984; **Identification:** identifiedBy: T. Griswold; dateIdentified: 2017; **Event:** samplingProtocol: malaise; eventDate: 2016-9-28; **Record Level:** type: PhysicalObject; language: en; rights: https://creativecommons.org/publicdomain/zero/1.0/; accessRights: http://vertnet.org/resources/norms.html; institutionCode: USDA-ARS; collectionCode: BBSL; basisOfRecord: PreservedSpecimen
- **Type status:** Other material. **Occurrence:** catalogNumber: SIMRU12176; recordedBy: K. A. Parys; individualCount: 1; sex: male; lifeStage: adult; preparations: pin; occurrenceID: urn:USDA-ARS:BBSL:SIMRU12176; **Taxon:** scientificName: Brachynomada (Melanomada) nimia (Snelling and Rozen, 1987); kingdom: Animalia; phylum: Arthropoda; class: Insecta; order: Hymenoptera; family: Apidae; genus: Brachynomada; subgenus: Melanomada; specificEpithet: nimia; scientificNameAuthorship: (Snelling and Rozen, 1987); **Location:** country: United States; stateProvince: Mississippi; county: Tallahatchie; locality: Tallahatchie National Wildlife Refuge; decimalLatitude: 33.760588; decimalLongitude: -90.150779; geodeticDatum: WGS1984; **Identification:** identifiedBy: T. Griswold; dateIdentified: 2017; **Event:** samplingProtocol: malaise; eventDate: 2016-11-7; **Record Level:** type: PhysicalObject; language: en; rights: https://creativecommons.org/publicdomain/zero/1.0/; accessRights: http://vertnet.org/resources/norms.html; institutionCode: USDA-ARS; collectionCode: BBSL; basisOfRecord: PreservedSpecimen
- **Type status:** Other material. **Occurrence:** catalogNumber: SIMRU12177; recordedBy: K. A. Parys; individualCount: 1; sex: female; lifeStage: adult; preparations: pin; occurrenceID: urn:USDA-ARS:BBSL:SIMRU12177; **Taxon:** scientificName: Brachynomada (Melanomada) nimia (Snelling and Rozen, 1987); kingdom: Animalia; phylum: Arthropoda; class: Insecta; order: Hymenoptera; family: Apidae; genus: Brachynomada; subgenus: Melanomada; specificEpithet: nimia; scientificNameAuthorship: (Snelling and Rozen, 1987); **Location:** country: United States; stateProvince: Mississippi; county: Tallahatchie; locality: Tallahatchie National Wildlife Refuge; decimalLatitude: 33.760588; decimalLongitude: -90.150779; geodeticDatum: WGS1984; **Identification:** identifiedBy: T. Griswold; dateIdentified: 2017; **Event:** samplingProtocol: malaise; eventDate: 2016-11-7; **Record Level:** type: PhysicalObject; language: en; rights: https://creativecommons.org/publicdomain/zero/1.0/; accessRights: http://vertnet.org/resources/norms.html; institutionCode: USDA-ARS; collectionCode: BBSL; basisOfRecord: PreservedSpecimen
- **Type status:** Other material. **Occurrence:** catalogNumber: SIMRU12178; recordedBy: K. A. Parys; individualCount: 1; sex: female; lifeStage: adult; preparations: pin; occurrenceID: urn:USDA-ARS:BBSL:SIMRU12178; **Taxon:** scientificName: Brachynomada (Melanomada) nimia (Snelling and Rozen, 1987); kingdom: Animalia; phylum: Arthropoda; class: Insecta; order: Hymenoptera; family: Apidae; genus: Brachynomada; subgenus: Melanomada; specificEpithet: nimia; scientificNameAuthorship: (Snelling and Rozen, 1987); **Location:** country: United States; stateProvince: Mississippi; county: Tallahatchie; locality: Tallahatchie National Wildlife Refuge; decimalLatitude: 33.760588; decimalLongitude: -90.150779; geodeticDatum: WGS1984; **Identification:** identifiedBy: T. Griswold; dateIdentified: 2017; **Event:** samplingProtocol: malaise; eventDate: 2016-11-7; **Record Level:** type: PhysicalObject; language: en; rights: https://creativecommons.org/publicdomain/zero/1.0/; accessRights: http://vertnet.org/resources/norms.html; institutionCode: USDA-ARS; collectionCode: BBSL; basisOfRecord: PreservedSpecimen
- **Type status:** Other material. **Occurrence:** catalogNumber: SIMRU12179; recordedBy: K. A. Parys; individualCount: 1; sex: female; lifeStage: adult; preparations: pin; occurrenceID: urn:USDA-ARS:SIMRU:SIMRU12179; **Taxon:** scientificName: Brachynomada (Melanomada) nimia (Snelling and Rozen, 1987); kingdom: Animalia; phylum: Arthropoda; class: Insecta; order: Hymenoptera; family: Apidae; genus: Brachynomada; subgenus: Melanomada; specificEpithet: nimia; scientificNameAuthorship: (Snelling and Rozen, 1987); **Location:** country: United States; stateProvince: Mississippi; county: Tallahatchie; locality: Tallahatchie National Wildlife Refuge; decimalLatitude: 33.760588; decimalLongitude: -90.150779; geodeticDatum: WGS1984; **Identification:** identifiedBy: T. Griswold; dateIdentified: 2017; **Event:** samplingProtocol: malaise; eventDate: 2016-11-7; **Record Level:** type: PhysicalObject; language: en; rights: https://creativecommons.org/publicdomain/zero/1.0/; accessRights: http://vertnet.org/resources/norms.html; institutionCode: USDA-ARS; collectionCode: SIMRU; basisOfRecord: PreservedSpecimen

##### Notes

Little is known about the distribution or specific biology of this species. The original type material was collected and described from Chase and Woodson Counties in Kansas, with additional records recently from Illinois (Snelling and Rozen 1987, Ascher and Pickering 2017). Images of the collection labels from Woodson County specimens indicate that they were collected from *Amphiachyris dracunculoides* D.C. Nutt (*GBIF 2017a*). All known members of this group are cleptoparasitic nest associates of *Exomalopsis* spp. (Rozen 1984).

Eight specimens were collected by malaise trap during the fall (autumn) of 2016 in Tallahatchie National Wildlife Refuge, located near Phillip, Mississippi in Tallahatchie County (Fig. 5). The malaise traps were located not far from each other on the shore of an oxbow lake filled with emergent vegetation.

#### Epeolini

#### Triepeolus subnitens

Cockerell & Timberlake, 1929

##### Materials

- **Type status:** Other material. **Occurrence:** catalogNumber: SIMRU0791; recordedBy: K. A. Parys; individualCount: 1; sex: female; lifeStage: adult; preparations: pin; occurrenceID: urn:USDA-ARS:BBSL:SIMRU0791; **Taxon:** scientificName: Triepeolus subnitens Cockerell and Timberlake, 1929; kingdom: Animalia; phylum: Arthropoda; class: Insecta; order: Hymenoptera; family: Apidae; genus: Triepeolus; specificEpithet: subnitens; scientificNameAuthorship: Cockerell and Timberlake, 1929; **Location:** country: United States; stateProvince: Mississippi; county: Sunflower; locality: Heathman Plantation, Holly Ridge; decimalLatitude: 33.462079; decimalLongitude: -90.707222; geodeticDatum: WGS1984; **Identification:** identifiedBy: T. Griswold; dateIdentified: 2017; **Event:** samplingProtocol: malaise; eventDate: 2015-7-21; **Record Level:** type: PhysicalObject; language: en; rights: https://creativecommons.org/publicdomain/zero/1.0/; accessRights: http://vertnet.org/resources/norms.html; institutionCode: USDA-ARS; collectionCode: BBSL; basisOfRecord: PreservedSpecimen
- **Type status:** Other material. **Occurrence:** catalogNumber: SIMRU6666; recordedBy: K. A. Parys; individualCount: 1; sex: female; lifeStage: adult; preparations: pin; occurrenceID: urn:USDA-ARS:SIMRU:SIMRU6666; **Taxon:** scientificName: Triepeolus subnitens Cockerell and Timberlake, 1929; kingdom: Animalia; phylum: Arthropoda; class: Insecta; order: Hymenoptera; family: Apidae; genus: Triepeolus; specificEpithet: subnitens; scientificNameAuthorship: Cockerell and Timberlake, 1929; **Location:** country: United States; stateProvince: Mississippi; county: Sunflower; locality: Heathman Plantation, Holly Ridge; decimalLatitude: 33.462079; decimalLongitude: -90.707222; geodeticDatum: WGS1984; **Identification:** identifiedBy: T. Griswold; dateIdentified: 2017; **Event:** samplingProtocol: Bee Bowl (Blue); eventDate: 2016-6-30; **Record Level:** type: PhysicalObject; language: en; rights: https://creativecommons.org/publicdomain/zero/1.0/; accessRights: http://vertnet.org/resources/norms.html; institutionCode: USDA-ARS; collectionCode: SIMRU; basisOfRecord: PreservedSpecimen
- **Type status:** Other material. **Occurrence:** catalogNumber: SIMRU9876; recordedBy: K. A. Parys; individualCount: 1; sex: female; lifeStage: adult; preparations: pin; occurrenceID: urn:USDA-ARS:SIMRU:SIMRU9876; **Taxon:** scientificName: Triepeolus subnitens Cockerell and Timberlake, 1929; kingdom: Animalia; phylum: Arthropoda; class: Insecta; order: Hymenoptera; family: Apidae; genus: Triepeolus; specificEpithet: subnitens; scientificNameAuthorship: Cockerell and Timberlake, 1929; **Location:** country: United States; stateProvince: Mississippi; county: Sharkey; locality: Rolling Fork; decimalLatitude: 32.91696; decimalLongitude: -90.92081; geodeticDatum: WGS1984; **Identification:** identifiedBy: T. Griswold; dateIdentified: 2017; **Event:** samplingProtocol: Vane Trap (Blue); eventDate: 2016-8-10; **Record Level:** type: PhysicalObject; language: en; rights: https://creativecommons.org/publicdomain/zero/1.0/; accessRights: http://vertnet.org/resources/norms.html; institutionCode: USDA-ARS; collectionCode: SIMRU; basisOfRecord: PreservedSpecimen

##### Notes

The recent revision of *Triepeolus* lists the current distribution of this species as including Arizona, California, Kansas, Nevada, New Mexico, Oklahoma, Texas (east to Smith County) and Utah and south into México (Rightmyer 2008). Specimens have been taken from 4 May through 12 October and observed on a variety of host plants (Rightmyer 2008). This group is also cleptoparasitic, with *T. subnitens* observed entering a burrow of Svastra (Epimelissodes) obliqua (Say) (Hurd et al. 1980).

The three specimens reported from Mississippi were all females (Fig. 6) and collected during 2015 and 2016 in two counties (Sharkey and Sunflower). They were taken from both *Helianthus* sp. and from a blue vane trap located within a soybean field.

#### Apinae

#### Emphorini

#### Diadasia (Diadasia) enavata

(Cresson, 1872)

##### Materials

- **Type status:** Other material. **Occurrence:** catalogNumber: SIMRU0026; recordedBy: K. A. Parys; individualCount: 1; sex: female; lifeStage: adult; preparations: pin; occurrenceID: urn:USDA-ARS:SIMRU:SIMRU0026; **Taxon:** scientificName: Diadasia (Diadasia) enavata (Cresson, 1872); kingdom: Animalia; phylum: Arthropoda; class: Insecta; order: Hymenoptera; family: Apidae; genus: Diadasia; subgenus: Diadasia; specificEpithet: enavata; scientificNameAuthorship: (Cresson, 1872); **Location:** country: United States; stateProvince: Mississippi; county: Sunflower; locality: Heathman Plantation, Holly Ridge; decimalLatitude: 33.436181; decimalLongitude: -90.708557; geodeticDatum: WGS1984; **Identification:** identifiedBy: T. Griswold; dateIdentified: 2017; **Event:** samplingProtocol: Bee Bowl (Blue); eventDate: 2015-6-16; **Record Level:** type: PhysicalObject; language: en; rights: https://creativecommons.org/publicdomain/zero/1.0/; accessRights: http://vertnet.org/resources/norms.html; institutionCode: USDA-ARS; collectionCode: SIMRU; basisOfRecord: PreservedSpecimen
- **Type status:** Other material. **Occurrence:** catalogNumber: SIMRU0480; recordedBy: K. A. Parys; individualCount: 1; sex: female; lifeStage: adult; preparations: pin; occurrenceID: urn:USDA-ARS:SIMRU:SIMRU0480; **Taxon:** scientificName: Diadasia (Diadasia) enavata (Cresson, 1872); kingdom: Animalia; phylum: Arthropoda; class: Insecta; order: Hymenoptera; family: Apidae; genus: Diadasia; subgenus: Diadasia; specificEpithet: enavata; scientificNameAuthorship: (Cresson, 1872); **Location:** country: United States; stateProvince: Mississippi; county: Sunflower; locality: Heathman Plantation, Holly Ridge; decimalLatitude: 33.436181; decimalLongitude: -90.708557; geodeticDatum: WGS1984; **Identification:** identifiedBy: T. Griswold; dateIdentified: 2017; **Event:** samplingProtocol: Bee Bowl (Blue); eventDate: 2015-7-2; **Record Level:** type: PhysicalObject; language: en; rights: https://creativecommons.org/publicdomain/zero/1.0/; accessRights: http://vertnet.org/resources/norms.html; institutionCode: USDA-ARS; collectionCode: SIMRU; basisOfRecord: PreservedSpecimen
- **Type status:** Other material. **Occurrence:** catalogNumber: SIMRU0681; recordedBy: K. A. Parys; individualCount: 1; sex: female; lifeStage: adult; preparations: pin; occurrenceID: urn:USDA-ARS:SIMRU:SIMRU0681; **Taxon:** scientificName: Diadasia (Diadasia) enavata (Cresson, 1872); kingdom: Animalia; phylum: Arthropoda; class: Insecta; order: Hymenoptera; family: Apidae; genus: Diadasia; subgenus: Diadasia; specificEpithet: enavata; scientificNameAuthorship: (Cresson, 1872); **Location:** country: United States; stateProvince: Mississippi; county: Sunflower; locality: Heathman Plantation, Holly Ridge; decimalLatitude: 33.462079; decimalLongitude: -90.707222; geodeticDatum: WGS1984; **Identification:** identifiedBy: T. Griswold; dateIdentified: 2017; **Event:** samplingProtocol: Bee Bowl (Blue); eventDate: 2015-7-21; **Record Level:** type: PhysicalObject; language: en; rights: https://creativecommons.org/publicdomain/zero/1.0/; accessRights: http://vertnet.org/resources/norms.html; institutionCode: USDA-ARS; collectionCode: SIMRU; basisOfRecord: PreservedSpecimen
- **Type status:** Other material. **Occurrence:** catalogNumber: SIMRU0691; recordedBy: K. A. Parys; individualCount: 1; sex: male; lifeStage: adult; preparations: pin; occurrenceID: urn:USDA-ARS:SIMRU:SIMRU0691; **Taxon:** scientificName: Diadasia (Diadasia) enavata (Cresson, 1872); kingdom: Animalia; phylum: Arthropoda; class: Insecta; order: Hymenoptera; family: Apidae; genus: Diadasia; subgenus: Diadasia; specificEpithet: enavata; scientificNameAuthorship: (Cresson, 1872); **Location:** country: United States; stateProvince: Mississippi; county: Sunflower; locality: Heathman Plantation, Holly Ridge; decimalLatitude: 33.462079; decimalLongitude: -90.707222; geodeticDatum: WGS1984; **Identification:** identifiedBy: T. Griswold; dateIdentified: 2017; **Event:** samplingProtocol: Bee Bowl (Yellow); eventDate: 2015-7-21; **Record Level:** type: PhysicalObject; language: en; rights: https://creativecommons.org/publicdomain/zero/1.0/; accessRights: http://vertnet.org/resources/norms.html; institutionCode: USDA-ARS; collectionCode: SIMRU; basisOfRecord: PreservedSpecimen
- **Type status:** Other material. **Occurrence:** catalogNumber: SIMRU0692; recordedBy: K. A. Parys; individualCount: 1; sex: male; lifeStage: adult; preparations: pin; occurrenceID: urn:USDA-ARS:SIMRU:SIMRU0692; **Taxon:** scientificName: Diadasia (Diadasia) enavata (Cresson, 1872); kingdom: Animalia; phylum: Arthropoda; class: Insecta; order: Hymenoptera; family: Apidae; genus: Diadasia; subgenus: Diadasia; specificEpithet: enavata; scientificNameAuthorship: (Cresson, 1872); **Location:** country: United States; stateProvince: Mississippi; county: Sunflower; locality: Heathman Plantation, Holly Ridge; decimalLatitude: 33.462079; decimalLongitude: -90.707222; geodeticDatum: WGS1984; **Identification:** identifiedBy: T. Griswold; dateIdentified: 2017; **Event:** samplingProtocol: Bee Bowl (Yellow); eventDate: 2015-7-21; **Record Level:** type: PhysicalObject; language: en; rights: https://creativecommons.org/publicdomain/zero/1.0/; accessRights: http://vertnet.org/resources/norms.html; institutionCode: USDA-ARS; collectionCode: SIMRU; basisOfRecord: PreservedSpecimen
- **Type status:** Other material. **Occurrence:** catalogNumber: SIMRU0693; recordedBy: K. A. Parys; individualCount: 1; sex: male; lifeStage: adult; preparations: pin; occurrenceID: urn:USDA-ARS:SIMRU:SIMRU0693; **Taxon:** scientificName: Diadasia (Diadasia) enavata (Cresson, 1872); kingdom: Animalia; phylum: Arthropoda; class: Insecta; order: Hymenoptera; family: Apidae; genus: Diadasia; subgenus: Diadasia; specificEpithet: enavata; scientificNameAuthorship: (Cresson, 1872); **Location:** country: United States; stateProvince: Mississippi; county: Sunflower; locality: Heathman Plantation, Holly Ridge; decimalLatitude: 33.462079; decimalLongitude: -90.707222; geodeticDatum: WGS1984; **Identification:** identifiedBy: T. Griswold; dateIdentified: 2017; **Event:** samplingProtocol: Bee Bowl (Yellow); eventDate: 2015-7-21; **Record Level:** type: PhysicalObject; language: en; rights: https://creativecommons.org/publicdomain/zero/1.0/; accessRights: http://vertnet.org/resources/norms.html; institutionCode: USDA-ARS; collectionCode: SIMRU; basisOfRecord: PreservedSpecimen
- **Type status:** Other material. **Occurrence:** catalogNumber: SIMRU0694; recordedBy: K. A. Parys; individualCount: 1; sex: female; lifeStage: adult; preparations: pin; occurrenceID: urn:USDA-ARS:SIMRU:SIMRU0694; **Taxon:** scientificName: Diadasia (Diadasia) enavata (Cresson, 1872); kingdom: Animalia; phylum: Arthropoda; class: Insecta; order: Hymenoptera; family: Apidae; genus: Diadasia; subgenus: Diadasia; specificEpithet: enavata; scientificNameAuthorship: (Cresson, 1872); **Location:** country: United States; stateProvince: Mississippi; county: Sunflower; locality: Heathman Plantation, Holly Ridge; decimalLatitude: 33.462079; decimalLongitude: -90.707222; geodeticDatum: WGS1984; **Identification:** identifiedBy: T. Griswold; dateIdentified: 2017; **Event:** samplingProtocol: Bee Bowl (Yellow); eventDate: 2015-7-21; **Record Level:** type: PhysicalObject; language: en; rights: https://creativecommons.org/publicdomain/zero/1.0/; accessRights: http://vertnet.org/resources/norms.html; institutionCode: USDA-ARS; collectionCode: SIMRU; basisOfRecord: PreservedSpecimen
- **Type status:** Other material. **Occurrence:** catalogNumber: SIMRU0750; recordedBy: K. A. Parys; individualCount: 1; sex: male; lifeStage: adult; preparations: pin; occurrenceID: urn:USDA-ARS:SIMRU:SIMRU0750; **Taxon:** scientificName: Diadasia (Diadasia) enavata (Cresson, 1872); kingdom: Animalia; phylum: Arthropoda; class: Insecta; order: Hymenoptera; family: Apidae; genus: Diadasia; subgenus: Diadasia; specificEpithet: enavata; scientificNameAuthorship: (Cresson, 1872); **Location:** country: United States; stateProvince: Mississippi; county: Sunflower; locality: Heathman Plantation, Holly Ridge; decimalLatitude: 33.462079; decimalLongitude: -90.707222; geodeticDatum: WGS1984; **Identification:** identifiedBy: T. Griswold; dateIdentified: 2017; **Event:** samplingProtocol: Bee Bowl (Blue); eventDate: 2015-7-21; **Record Level:** type: PhysicalObject; language: en; rights: https://creativecommons.org/publicdomain/zero/1.0/; accessRights: http://vertnet.org/resources/norms.html; institutionCode: USDA-ARS; collectionCode: SIMRU; basisOfRecord: PreservedSpecimen
- **Type status:** Other material. **Occurrence:** catalogNumber: SIMRU0756; recordedBy: K. A. Parys; individualCount: 1; sex: female; lifeStage: adult; preparations: pin; occurrenceID: urn:USDA-ARS:SIMRU:SIMRU0756; **Taxon:** scientificName: Diadasia (Diadasia) enavata (Cresson, 1872); kingdom: Animalia; phylum: Arthropoda; class: Insecta; order: Hymenoptera; family: Apidae; genus: Diadasia; subgenus: Diadasia; specificEpithet: enavata; scientificNameAuthorship: (Cresson, 1872); **Location:** country: United States; stateProvince: Mississippi; county: Sunflower; locality: Heathman Plantation, Holly Ridge; decimalLatitude: 33.462079; decimalLongitude: -90.707222; geodeticDatum: WGS1984; **Identification:** identifiedBy: T. Griswold; dateIdentified: 2017; **Event:** samplingProtocol: Bee Bowl (Blue); eventDate: 2015-7-21; **Record Level:** type: PhysicalObject; language: en; rights: https://creativecommons.org/publicdomain/zero/1.0/; accessRights: http://vertnet.org/resources/norms.html; institutionCode: USDA-ARS; collectionCode: SIMRU; basisOfRecord: PreservedSpecimen
- **Type status:** Other material. **Occurrence:** catalogNumber: SIMRU1253; recordedBy: K. A. Parys; individualCount: 1; sex: male; lifeStage: adult; preparations: pin; occurrenceID: urn:USDA-ARS:SIMRU:SIMRU1253; **Taxon:** scientificName: Diadasia (Diadasia) enavata (Cresson, 1872); kingdom: Animalia; phylum: Arthropoda; class: Insecta; order: Hymenoptera; family: Apidae; genus: Diadasia; subgenus: Diadasia; specificEpithet: enavata; scientificNameAuthorship: (Cresson, 1872); **Location:** country: United States; stateProvince: Mississippi; county: Sharkey; locality: Cary; decimalLatitude: 32.785538; decimalLongitude: -90.964959; geodeticDatum: WGS1984; **Identification:** identifiedBy: T. Griswold; dateIdentified: 2017; **Event:** samplingProtocol: Bee Bowl (Blue); eventDate: 2015-7-28; **Record Level:** type: PhysicalObject; language: en; rights: https://creativecommons.org/publicdomain/zero/1.0/; accessRights: http://vertnet.org/resources/norms.html; institutionCode: USDA-ARS; collectionCode: SIMRU; basisOfRecord: PreservedSpecimen
- **Type status:** Other material. **Occurrence:** catalogNumber: SIMRU1288; recordedBy: K. A. Parys; individualCount: 1; sex: female; lifeStage: adult; preparations: pin; occurrenceID: urn:USDA-ARS:SIMRU:SIMRU1288; **Taxon:** scientificName: Diadasia (Diadasia) enavata (Cresson, 1872); kingdom: Animalia; phylum: Arthropoda; class: Insecta; order: Hymenoptera; family: Apidae; genus: Diadasia; subgenus: Diadasia; specificEpithet: enavata; scientificNameAuthorship: (Cresson, 1872); **Location:** country: United States; stateProvince: Mississippi; county: Sunflower; locality: Heathman Plantation, Holly Ridge; decimalLatitude: 33.462079; decimalLongitude: -90.707222; geodeticDatum: WGS1984; **Identification:** identifiedBy: T. Griswold; dateIdentified: 2017; **Event:** samplingProtocol: malaise; eventDate: 2015-7-21; **Record Level:** type: PhysicalObject; language: en; rights: https://creativecommons.org/publicdomain/zero/1.0/; accessRights: http://vertnet.org/resources/norms.html; institutionCode: USDA-ARS; collectionCode: SIMRU; basisOfRecord: PreservedSpecimen
- **Type status:** Other material. **Occurrence:** catalogNumber: SIMRU1716; recordedBy: K. A. Parys; individualCount: 1; sex: male; lifeStage: adult; preparations: pin; occurrenceID: urn:USDA-ARS:SIMRU:SIMRU1716; **Taxon:** scientificName: Diadasia (Diadasia) enavata (Cresson, 1872); kingdom: Animalia; phylum: Arthropoda; class: Insecta; order: Hymenoptera; family: Apidae; genus: Diadasia; subgenus: Diadasia; specificEpithet: enavata; scientificNameAuthorship: (Cresson, 1872); **Location:** country: United States; stateProvince: Mississippi; county: Sunflower; locality: Heathman Plantation, Holly Ridge; decimalLatitude: 33.462079; decimalLongitude: -90.707222; geodeticDatum: WGS1984; **Identification:** identifiedBy: T. Griswold; dateIdentified: 2017; **Event:** samplingProtocol: Bee Bowl (Yellow); eventDate: 2015-8-5; **Record Level:** type: PhysicalObject; language: en; rights: https://creativecommons.org/publicdomain/zero/1.0/; accessRights: http://vertnet.org/resources/norms.html; institutionCode: USDA-ARS; collectionCode: SIMRU; basisOfRecord: PreservedSpecimen
- **Type status:** Other material. **Occurrence:** catalogNumber: SIMRU2044; recordedBy: K. A. Parys; individualCount: 1; sex: male; lifeStage: adult; preparations: pin; occurrenceID: urn:USDA-ARS:SIMRU:SIMRU2044; **Taxon:** scientificName: Diadasia (Diadasia) enavata (Cresson, 1872); kingdom: Animalia; phylum: Arthropoda; class: Insecta; order: Hymenoptera; family: Apidae; genus: Diadasia; subgenus: Diadasia; specificEpithet: enavata; scientificNameAuthorship: (Cresson, 1872); **Location:** country: United States; stateProvince: Mississippi; county: Sunflower; locality: Heathman Plantation, Holly Ridge; decimalLatitude: 33.462079; decimalLongitude: -90.707222; geodeticDatum: WGS1984; **Identification:** identifiedBy: T. Griswold; dateIdentified: 2017; **Event:** samplingProtocol: Bee Bowl (Yellow); eventDate: 2015-8-5; **Record Level:** type: PhysicalObject; language: en; rights: https://creativecommons.org/publicdomain/zero/1.0/; accessRights: http://vertnet.org/resources/norms.html; institutionCode: USDA-ARS; collectionCode: SIMRU; basisOfRecord: PreservedSpecimen
- **Type status:** Other material. **Occurrence:** catalogNumber: SIMRU2095; recordedBy: K. A. Parys; individualCount: 1; sex: male; lifeStage: adult; preparations: pin; occurrenceID: urn:USDA-ARS:SIMRU:SIMRU2095; **Taxon:** scientificName: Diadasia (Diadasia) enavata (Cresson, 1872); kingdom: Animalia; phylum: Arthropoda; class: Insecta; order: Hymenoptera; family: Apidae; genus: Diadasia; subgenus: Diadasia; specificEpithet: enavata; scientificNameAuthorship: (Cresson, 1872); **Location:** country: United States; stateProvince: Mississippi; county: Sunflower; locality: Heathman Plantation, Holly Ridge; decimalLatitude: 33.462079; decimalLongitude: -90.707222; geodeticDatum: WGS1984; **Identification:** identifiedBy: T. Griswold; dateIdentified: 2017; **Event:** samplingProtocol: Bee Bowl (Blue); eventDate: 2015-8-5; **Record Level:** type: PhysicalObject; language: en; rights: https://creativecommons.org/publicdomain/zero/1.0/; accessRights: http://vertnet.org/resources/norms.html; institutionCode: USDA-ARS; collectionCode: SIMRU; basisOfRecord: PreservedSpecimen
- **Type status:** Other material. **Occurrence:** catalogNumber: SIMRU2096; recordedBy: K. A. Parys; individualCount: 1; sex: female; lifeStage: adult; preparations: pin; occurrenceID: urn:USDA-ARS:SIMRU:SIMRU2096; **Taxon:** scientificName: Diadasia (Diadasia) enavata (Cresson, 1872); kingdom: Animalia; phylum: Arthropoda; class: Insecta; order: Hymenoptera; family: Apidae; genus: Diadasia; subgenus: Diadasia; specificEpithet: enavata; scientificNameAuthorship: (Cresson, 1872); **Location:** country: United States; stateProvince: Mississippi; county: Sunflower; locality: Heathman Plantation, Holly Ridge; decimalLatitude: 33.462079; decimalLongitude: -90.707222; geodeticDatum: WGS1984; **Identification:** identifiedBy: T. Griswold; dateIdentified: 2017; **Event:** samplingProtocol: Bee Bowl (Blue); eventDate: 2015-8-5; **Record Level:** type: PhysicalObject; language: en; rights: https://creativecommons.org/publicdomain/zero/1.0/; accessRights: http://vertnet.org/resources/norms.html; institutionCode: USDA-ARS; collectionCode: SIMRU; basisOfRecord: PreservedSpecimen
- **Type status:** Other material. **Occurrence:** catalogNumber: SIMRU2097; recordedBy: K. A. Parys; individualCount: 1; sex: male; lifeStage: adult; preparations: pin; occurrenceID: urn:USDA-ARS:SIMRU:SIMRU2097; **Taxon:** scientificName: Diadasia (Diadasia) enavata (Cresson, 1872); kingdom: Animalia; phylum: Arthropoda; class: Insecta; order: Hymenoptera; family: Apidae; genus: Diadasia; subgenus: Diadasia; specificEpithet: enavata; scientificNameAuthorship: (Cresson, 1872); **Location:** country: United States; stateProvince: Mississippi; county: Sharkey; locality: Cary; decimalLatitude: 32.785538; decimalLongitude: -90.964959; geodeticDatum: WGS1984; **Identification:** identifiedBy: T. Griswold; dateIdentified: 2017; **Event:** samplingProtocol: Bee Bowl (Blue); eventDate: 2015-8-5; **Record Level:** type: PhysicalObject; language: en; rights: https://creativecommons.org/publicdomain/zero/1.0/; accessRights: http://vertnet.org/resources/norms.html; institutionCode: USDA-ARS; collectionCode: SIMRU; basisOfRecord: PreservedSpecimen
- **Type status:** Other material. **Occurrence:** catalogNumber: SIMRU2183; recordedBy: K. A. Parys; individualCount: 1; sex: male; lifeStage: adult; preparations: pin; occurrenceID: urn:USDA-ARS:BBSL:SIMRU2183; **Taxon:** scientificName: Diadasia (Diadasia) enavata (Cresson, 1872); kingdom: Animalia; phylum: Arthropoda; class: Insecta; order: Hymenoptera; family: Apidae; genus: Diadasia; subgenus: Diadasia; specificEpithet: enavata; scientificNameAuthorship: (Cresson, 1872); **Location:** country: United States; stateProvince: Mississippi; county: Sunflower; locality: Heathman Plantation, Holly Ridge; decimalLatitude: 33.462079; decimalLongitude: -90.707222; geodeticDatum: WGS1984; **Identification:** identifiedBy: T. Griswold; dateIdentified: 2017; **Event:** samplingProtocol: Bee Bowl (White); eventDate: 2015-8-11; **Record Level:** type: PhysicalObject; language: en; rights: https://creativecommons.org/publicdomain/zero/1.0/; accessRights: http://vertnet.org/resources/norms.html; institutionCode: USDA-ARS; collectionCode: BBSL; basisOfRecord: PreservedSpecimen
- **Type status:** Other material. **Occurrence:** catalogNumber: SIMRU2236; recordedBy: K. A. Parys; individualCount: 1; sex: male; lifeStage: adult; preparations: pin; occurrenceID: urn:USDA-ARS:SIMRU:SIMRU2236; **Taxon:** scientificName: Diadasia (Diadasia) enavata (Cresson, 1872); kingdom: Animalia; phylum: Arthropoda; class: Insecta; order: Hymenoptera; family: Apidae; genus: Diadasia; subgenus: Diadasia; specificEpithet: enavata; scientificNameAuthorship: (Cresson, 1872); **Location:** country: United States; stateProvince: Mississippi; county: Sunflower; locality: Heathman Plantation, Holly Ridge; decimalLatitude: 33.462079; decimalLongitude: -90.707222; geodeticDatum: WGS1984; **Identification:** identifiedBy: T. Griswold; dateIdentified: 2017; **Event:** samplingProtocol: Bee Bowl (Yellow); eventDate: 2015-8-11; **Record Level:** type: PhysicalObject; language: en; rights: https://creativecommons.org/publicdomain/zero/1.0/; accessRights: http://vertnet.org/resources/norms.html; institutionCode: USDA-ARS; collectionCode: SIMRU; basisOfRecord: PreservedSpecimen
- **Type status:** Other material. **Occurrence:** catalogNumber: SIMRU2260; recordedBy: K. A. Parys; individualCount: 1; sex: male; lifeStage: adult; preparations: pin; occurrenceID: urn:USDA-ARS:BBSL:SIMRU2260; **Taxon:** scientificName: Diadasia (Diadasia) enavata (Cresson, 1872); kingdom: Animalia; phylum: Arthropoda; class: Insecta; order: Hymenoptera; family: Apidae; genus: Diadasia; subgenus: Diadasia; specificEpithet: enavata; scientificNameAuthorship: (Cresson, 1872); **Location:** country: United States; stateProvince: Mississippi; county: Bolivar; locality: Old Hwy 61; decimalLatitude: 33.821; decimalLongitude: -90.7252; geodeticDatum: WGS1984; **Identification:** identifiedBy: T. Griswold; dateIdentified: 2017; **Event:** samplingProtocol: Bee Bowl (Blue); eventDate: 2015-8-11; **Record Level:** type: PhysicalObject; language: en; rights: https://creativecommons.org/publicdomain/zero/1.0/; accessRights: http://vertnet.org/resources/norms.html; institutionCode: USDA-ARS; collectionCode: BBSL; basisOfRecord: PreservedSpecimen
- **Type status:** Other material. **Occurrence:** catalogNumber: SIMRU2261; recordedBy: K. A. Parys; individualCount: 1; sex: male; lifeStage: adult; preparations: pin; occurrenceID: urn:USDA-ARS:BBSL:SIMRU2261; **Taxon:** scientificName: Diadasia (Diadasia) enavata (Cresson, 1872); kingdom: Animalia; phylum: Arthropoda; class: Insecta; order: Hymenoptera; family: Apidae; genus: Diadasia; subgenus: Diadasia; specificEpithet: enavata; scientificNameAuthorship: (Cresson, 1872); **Location:** country: United States; stateProvince: Mississippi; county: Sunflower; locality: Heathman Plantation, Holly Ridge; decimalLatitude: 33.462079; decimalLongitude: -90.707222; geodeticDatum: WGS1984; **Identification:** identifiedBy: T. Griswold; dateIdentified: 2017; **Event:** samplingProtocol: Bee Bowl (Blue); eventDate: 2015-8-11; **Record Level:** type: PhysicalObject; language: en; rights: https://creativecommons.org/publicdomain/zero/1.0/; accessRights: http://vertnet.org/resources/norms.html; institutionCode: USDA-ARS; collectionCode: BBSL; basisOfRecord: PreservedSpecimen
- **Type status:** Other material. **Occurrence:** catalogNumber: SIMRU2262; recordedBy: K. A. Parys; individualCount: 1; sex: male; lifeStage: adult; preparations: pin; occurrenceID: urn:USDA-ARS:SIMRU:SIMRU2262; **Taxon:** scientificName: Diadasia (Diadasia) enavata (Cresson, 1872); kingdom: Animalia; phylum: Arthropoda; class: Insecta; order: Hymenoptera; family: Apidae; genus: Diadasia; subgenus: Diadasia; specificEpithet: enavata; scientificNameAuthorship: (Cresson, 1872); **Location:** country: United States; stateProvince: Mississippi; county: Bolivar; locality: Alcorn Research Farm, Mound Bayou; decimalLatitude: 33.871265; decimalLongitude: -90.699184; geodeticDatum: WGS1984; **Identification:** identifiedBy: T. Griswold; dateIdentified: 2017; **Event:** samplingProtocol: Bee Bowl (Blue); eventDate: 2015-8-11; **Record Level:** type: PhysicalObject; language: en; rights: https://creativecommons.org/publicdomain/zero/1.0/; accessRights: http://vertnet.org/resources/norms.html; institutionCode: USDA-ARS; collectionCode: SIMRU; basisOfRecord: PreservedSpecimen
- **Type status:** Other material. **Occurrence:** catalogNumber: SIMRU2263; recordedBy: K. A. Parys; individualCount: 1; sex: male; lifeStage: adult; preparations: pin; occurrenceID: urn:USDA-ARS:SIMRU:SIMRU2263; **Taxon:** scientificName: Diadasia (Diadasia) enavata (Cresson, 1872); kingdom: Animalia; phylum: Arthropoda; class: Insecta; order: Hymenoptera; family: Apidae; genus: Diadasia; subgenus: Diadasia; specificEpithet: enavata; scientificNameAuthorship: (Cresson, 1872); **Location:** country: United States; stateProvince: Mississippi; county: Sunflower; locality: Heathman Plantation, Holly Ridge; decimalLatitude: 22.469196; decimalLongitude: -90.707709; geodeticDatum: WGS1984; **Identification:** identifiedBy: T. Griswold; dateIdentified: 2017; **Event:** samplingProtocol: Bee Bowl (Blue); eventDate: 2015-8-11; **Record Level:** type: PhysicalObject; language: en; rights: https://creativecommons.org/publicdomain/zero/1.0/; accessRights: http://vertnet.org/resources/norms.html; institutionCode: USDA-ARS; collectionCode: SIMRU; basisOfRecord: PreservedSpecimen
- **Type status:** Other material. **Occurrence:** catalogNumber: SIMRU2265; recordedBy: K. A. Parys; individualCount: 1; sex: male; lifeStage: adult; preparations: pin; occurrenceID: urn:USDA-ARS:SIMRU:SIMRU2265; **Taxon:** scientificName: Diadasia (Diadasia) enavata (Cresson, 1872); kingdom: Animalia; phylum: Arthropoda; class: Insecta; order: Hymenoptera; family: Apidae; genus: Diadasia; subgenus: Diadasia; specificEpithet: enavata; scientificNameAuthorship: (Cresson, 1872); **Location:** country: United States; stateProvince: Mississippi; county: Sunflower; locality: Heathman Plantation, Holly Ridge; decimalLatitude: 22.469196; decimalLongitude: -90.707709; geodeticDatum: WGS1984; **Identification:** identifiedBy: T. Griswold; dateIdentified: 2017; **Event:** samplingProtocol: Bee Bowl (Blue); eventDate: 2015-8-11; **Record Level:** type: PhysicalObject; language: en; rights: https://creativecommons.org/publicdomain/zero/1.0/; accessRights: http://vertnet.org/resources/norms.html; institutionCode: USDA-ARS; collectionCode: SIMRU; basisOfRecord: PreservedSpecimen
- **Type status:** Other material. **Occurrence:** catalogNumber: SIMRU2266; recordedBy: K. A. Parys; individualCount: 1; sex: male; lifeStage: adult; preparations: pin; occurrenceID: urn:USDA-ARS:SIMRU:SIMRU2266; **Taxon:** scientificName: Diadasia (Diadasia) enavata (Cresson, 1872); kingdom: Animalia; phylum: Arthropoda; class: Insecta; order: Hymenoptera; family: Apidae; genus: Diadasia; subgenus: Diadasia; specificEpithet: enavata; scientificNameAuthorship: (Cresson, 1872); **Location:** country: United States; stateProvince: Mississippi; county: Sunflower; locality: Heathman Plantation, Holly Ridge; decimalLatitude: 22.469196; decimalLongitude: -90.707709; geodeticDatum: WGS1984; **Identification:** identifiedBy: T. Griswold; dateIdentified: 2017; **Event:** samplingProtocol: Bee Bowl (Blue); eventDate: 2015-8-11; **Record Level:** type: PhysicalObject; language: en; rights: https://creativecommons.org/publicdomain/zero/1.0/; accessRights: http://vertnet.org/resources/norms.html; institutionCode: USDA-ARS; collectionCode: SIMRU; basisOfRecord: PreservedSpecimen
- **Type status:** Other material. **Occurrence:** catalogNumber: SIMRU2405; recordedBy: K. A. Parys; individualCount: 1; sex: female; lifeStage: adult; preparations: pin; occurrenceID: urn:USDA-ARS:SIMRU:SIMRU2405; **Taxon:** scientificName: Diadasia (Diadasia) enavata (Cresson, 1872); kingdom: Animalia; phylum: Arthropoda; class: Insecta; order: Hymenoptera; family: Apidae; genus: Diadasia; subgenus: Diadasia; specificEpithet: enavata; scientificNameAuthorship: (Cresson, 1872); **Location:** country: United States; stateProvince: Mississippi; county: Sunflower; locality: Heathman Plantation, Holly Ridge; decimalLatitude: 33.462079; decimalLongitude: -90.707222; geodeticDatum: WGS1984; **Identification:** identifiedBy: T. Griswold; dateIdentified: 2017; **Event:** samplingProtocol: Malaise; eventDate: 2015-8-20; **Record Level:** type: PhysicalObject; language: en; rights: https://creativecommons.org/publicdomain/zero/1.0/; accessRights: http://vertnet.org/resources/norms.html; institutionCode: USDA-ARS; collectionCode: SIMRU; basisOfRecord: PreservedSpecimen
- **Type status:** Other material. **Occurrence:** catalogNumber: SIMRU2406; recordedBy: K. A. Parys; individualCount: 1; sex: male; lifeStage: adult; preparations: pin; occurrenceID: urn:USDA-ARS:SIMRU:SIMRU2406; **Taxon:** scientificName: Diadasia (Diadasia) enavata (Cresson, 1872); kingdom: Animalia; phylum: Arthropoda; class: Insecta; order: Hymenoptera; family: Apidae; genus: Diadasia; subgenus: Diadasia; specificEpithet: enavata; scientificNameAuthorship: (Cresson, 1872); **Location:** country: United States; stateProvince: Mississippi; county: Sunflower; locality: Heathman Plantation, Holly Ridge; decimalLatitude: 33.462079; decimalLongitude: -90.707222; geodeticDatum: WGS1984; **Identification:** identifiedBy: T. Griswold; dateIdentified: 2017; **Event:** samplingProtocol: Malaise; eventDate: 2015-8-20; **Record Level:** type: PhysicalObject; language: en; rights: https://creativecommons.org/publicdomain/zero/1.0/; accessRights: http://vertnet.org/resources/norms.html; institutionCode: USDA-ARS; collectionCode: SIMRU; basisOfRecord: PreservedSpecimen
- **Type status:** Other material. **Occurrence:** catalogNumber: SIMRU2408; recordedBy: K. A. Parys; individualCount: 1; sex: male; lifeStage: adult; preparations: pin; occurrenceID: urn:USDA-ARS:SIMRU:SIMRU2408; **Taxon:** scientificName: Diadasia (Diadasia) enavata (Cresson, 1872); kingdom: Animalia; phylum: Arthropoda; class: Insecta; order: Hymenoptera; family: Apidae; genus: Diadasia; subgenus: Diadasia; specificEpithet: enavata; scientificNameAuthorship: (Cresson, 1872); **Location:** country: United States; stateProvince: Mississippi; county: Bolivar; locality: Alcorn Research Farm, Mound Bayou; decimalLatitude: 33.871265; decimalLongitude: -90.699184; geodeticDatum: WGS1984; **Identification:** identifiedBy: T. Griswold; dateIdentified: 2017; **Event:** samplingProtocol: Malaise; eventDate: 2015-8-20; **Record Level:** type: PhysicalObject; language: en; rights: https://creativecommons.org/publicdomain/zero/1.0/; accessRights: http://vertnet.org/resources/norms.html; institutionCode: USDA-ARS; collectionCode: SIMRU; basisOfRecord: PreservedSpecimen
- **Type status:** Other material. **Occurrence:** catalogNumber: SIMRU5162; recordedBy: K. A. Parys; individualCount: 1; sex: male; lifeStage: adult; preparations: pin; occurrenceID: urn:USDA-ARS:SIMRU:SIMRU5162; **Taxon:** scientificName: Diadasia (Diadasia) enavata (Cresson, 1872); kingdom: Animalia; phylum: Arthropoda; class: Insecta; order: Hymenoptera; family: Apidae; genus: Diadasia; subgenus: Diadasia; specificEpithet: enavata; scientificNameAuthorship: (Cresson, 1872); **Location:** country: United States; stateProvince: Mississippi; county: Bolivar; locality: Alcorn Research Farm, Mound Bayou; decimalLatitude: 33.871265; decimalLongitude: -90.699184; geodeticDatum: WGS1984; **Identification:** identifiedBy: K. A. Parys; dateIdentified: 2017; **Event:** samplingProtocol: Vane Trap (Blue); eventDate: 2016-6-7; **Record Level:** type: PhysicalObject; language: en; rights: https://creativecommons.org/publicdomain/zero/1.0/; accessRights: http://vertnet.org/resources/norms.html; institutionCode: USDA-ARS; collectionCode: SIMRU; basisOfRecord: PreservedSpecimen
- **Type status:** Other material. **Occurrence:** catalogNumber: SIMRU5163; recordedBy: K. A. Parys; individualCount: 1; sex: male; lifeStage: adult; preparations: pin; occurrenceID: urn:USDA-ARS:SIMRU:SIMRU5163; **Taxon:** scientificName: Diadasia (Diadasia) enavata (Cresson, 1872); kingdom: Animalia; phylum: Arthropoda; class: Insecta; order: Hymenoptera; family: Apidae; genus: Diadasia; subgenus: Diadasia; specificEpithet: enavata; scientificNameAuthorship: (Cresson, 1872); **Location:** country: United States; stateProvince: Mississippi; county: Bolivar; locality: Alcorn Research Farm, Mound Bayou; decimalLatitude: 33.871265; decimalLongitude: -90.699184; geodeticDatum: WGS1984; **Identification:** identifiedBy: K. A. Parys; dateIdentified: 2017; **Event:** samplingProtocol: Vane Trap (Blue); eventDate: 2016-6-7; **Record Level:** type: PhysicalObject; language: en; rights: https://creativecommons.org/publicdomain/zero/1.0/; accessRights: http://vertnet.org/resources/norms.html; institutionCode: USDA-ARS; collectionCode: SIMRU; basisOfRecord: PreservedSpecimen
- **Type status:** Other material. **Occurrence:** catalogNumber: SIMRU5165; recordedBy: K. A. Parys; individualCount: 1; sex: male; lifeStage: adult; preparations: pin; occurrenceID: urn:USDA-ARS:SIMRU:SIMRU5165; **Taxon:** scientificName: Diadasia (Diadasia) enavata (Cresson, 1872); kingdom: Animalia; phylum: Arthropoda; class: Insecta; order: Hymenoptera; family: Apidae; genus: Diadasia; subgenus: Diadasia; specificEpithet: enavata; scientificNameAuthorship: (Cresson, 1872); **Location:** country: United States; stateProvince: Mississippi; county: Sunflower; locality: Heathman Plantation, Holly Ridge; decimalLatitude: 33.462079; decimalLongitude: -90.707222; geodeticDatum: WGS1984; **Identification:** identifiedBy: K. A. Parys; dateIdentified: 2017; **Event:** samplingProtocol: Vane Trap (Blue); eventDate: 2016-6-7; **Record Level:** type: PhysicalObject; language: en; rights: https://creativecommons.org/publicdomain/zero/1.0/; accessRights: http://vertnet.org/resources/norms.html; institutionCode: USDA-ARS; collectionCode: SIMRU; basisOfRecord: PreservedSpecimen
- **Type status:** Other material. **Occurrence:** catalogNumber: SIMRU5880; recordedBy: K. A. Parys; individualCount: 1; sex: female; lifeStage: adult; preparations: pin; occurrenceID: urn:USDA-ARS:SIMRU:SIMRU5880; **Taxon:** scientificName: Diadasia (Diadasia) enavata (Cresson, 1872); kingdom: Animalia; phylum: Arthropoda; class: Insecta; order: Hymenoptera; family: Apidae; genus: Diadasia; subgenus: Diadasia; specificEpithet: enavata; scientificNameAuthorship: (Cresson, 1872); **Location:** country: United States; stateProvince: Mississippi; county: Bolivar; locality: Alcorn Research Farm, Mound Bayou; decimalLatitude: 33.871265; decimalLongitude: -90.699184; geodeticDatum: WGS1984; **Identification:** identifiedBy: K. A. Parys; dateIdentified: 2017; **Event:** samplingProtocol: Vane Trap (Blue); eventDate: 2016-7-1; **Record Level:** type: PhysicalObject; language: en; rights: https://creativecommons.org/publicdomain/zero/1.0/; accessRights: http://vertnet.org/resources/norms.html; institutionCode: USDA-ARS; collectionCode: SIMRU; basisOfRecord: PreservedSpecimen
- **Type status:** Other material. **Occurrence:** catalogNumber: SIMRU5951; recordedBy: K. A. Parys; individualCount: 1; sex: male; lifeStage: adult; preparations: pin; occurrenceID: urn:USDA-ARS:SIMRU:SIMRU5951; **Taxon:** scientificName: Diadasia (Diadasia) enavata (Cresson, 1872); kingdom: Animalia; phylum: Arthropoda; class: Insecta; order: Hymenoptera; family: Apidae; genus: Diadasia; subgenus: Diadasia; specificEpithet: enavata; scientificNameAuthorship: (Cresson, 1872); **Location:** country: United States; stateProvince: Mississippi; county: Bolivar; locality: Alcorn Research Farm, Mound Bayou; decimalLatitude: 33.871265; decimalLongitude: -90.699184; geodeticDatum: WGS1984; **Identification:** identifiedBy: K. A. Parys; dateIdentified: 2017; **Event:** samplingProtocol: Vane Trap (Blue); eventDate: 2016-7-1; **Record Level:** type: PhysicalObject; language: en; rights: https://creativecommons.org/publicdomain/zero/1.0/; accessRights: http://vertnet.org/resources/norms.html; institutionCode: USDA-ARS; collectionCode: SIMRU; basisOfRecord: PreservedSpecimen
- **Type status:** Other material. **Occurrence:** catalogNumber: SIMRU5962; recordedBy: K. A. Parys; individualCount: 1; sex: male; lifeStage: adult; preparations: pin; occurrenceID: urn:USDA-ARS:SIMRU:SIMRU5962; **Taxon:** scientificName: Diadasia (Diadasia) enavata (Cresson, 1872); kingdom: Animalia; phylum: Arthropoda; class: Insecta; order: Hymenoptera; family: Apidae; genus: Diadasia; subgenus: Diadasia; specificEpithet: enavata; scientificNameAuthorship: (Cresson, 1872); **Location:** country: United States; stateProvince: Mississippi; county: Bolivar; locality: Alcorn Research Farm, Mound Bayou; decimalLatitude: 33.871265; decimalLongitude: -90.699184; geodeticDatum: WGS1984; **Identification:** identifiedBy: K. A. Parys; dateIdentified: 2017; **Event:** samplingProtocol: Vane Trap (Blue); eventDate: 2016-7-1; **Record Level:** type: PhysicalObject; language: en; rights: https://creativecommons.org/publicdomain/zero/1.0/; accessRights: http://vertnet.org/resources/norms.html; institutionCode: USDA-ARS; collectionCode: SIMRU; basisOfRecord: PreservedSpecimen
- **Type status:** Other material. **Occurrence:** catalogNumber: SIMRU5974; recordedBy: K. A. Parys; individualCount: 1; sex: female; lifeStage: adult; preparations: pin; occurrenceID: urn:USDA-ARS:SIMRU:SIMRU5974; **Taxon:** scientificName: Diadasia (Diadasia) enavata (Cresson, 1872); kingdom: Animalia; phylum: Arthropoda; class: Insecta; order: Hymenoptera; family: Apidae; genus: Diadasia; subgenus: Diadasia; specificEpithet: enavata; scientificNameAuthorship: (Cresson, 1872); **Location:** country: United States; stateProvince: Mississippi; county: Bolivar; locality: Alcorn Research Farm, Mound Bayou; decimalLatitude: 33.871265; decimalLongitude: -90.699184; geodeticDatum: WGS1984; **Identification:** identifiedBy: K. A. Parys; dateIdentified: 2017; **Event:** samplingProtocol: Vane Trap (Blue); eventDate: 2016-7-1; **Record Level:** type: PhysicalObject; language: en; rights: https://creativecommons.org/publicdomain/zero/1.0/; accessRights: http://vertnet.org/resources/norms.html; institutionCode: USDA-ARS; collectionCode: SIMRU; basisOfRecord: PreservedSpecimen
- **Type status:** Other material. **Occurrence:** catalogNumber: SIMRU6801; recordedBy: K. A. Parys; individualCount: 1; sex: male; lifeStage: adult; preparations: pin; occurrenceID: urn:USDA-ARS:SIMRU:SIMRU6801; **Taxon:** scientificName: Diadasia (Diadasia) enavata (Cresson, 1872); kingdom: Animalia; phylum: Arthropoda; class: Insecta; order: Hymenoptera; family: Apidae; genus: Diadasia; subgenus: Diadasia; specificEpithet: enavata; scientificNameAuthorship: (Cresson, 1872); **Location:** country: United States; stateProvince: Mississippi; county: Washington; locality: Prewitt Tree Farm, Leland; decimalLatitude: 33.346275; decimalLongitude: -90.914549; geodeticDatum: WGS1984; **Identification:** identifiedBy: K. A. Parys; dateIdentified: 2017; **Event:** samplingProtocol: Vane Trap (Blue); eventDate: 2016-6-21; **Record Level:** type: PhysicalObject; language: en; rights: https://creativecommons.org/publicdomain/zero/1.0/; accessRights: http://vertnet.org/resources/norms.html; institutionCode: USDA-ARS; collectionCode: SIMRU; basisOfRecord: PreservedSpecimen
- **Type status:** Other material. **Occurrence:** catalogNumber: SIMRU6808; recordedBy: K. A. Parys; individualCount: 1; sex: male; lifeStage: adult; preparations: pin; occurrenceID: urn:USDA-ARS:SIMRU:SIMRU6808; **Taxon:** scientificName: Diadasia (Diadasia) enavata (Cresson, 1872); kingdom: Animalia; phylum: Arthropoda; class: Insecta; order: Hymenoptera; family: Apidae; genus: Diadasia; subgenus: Diadasia; specificEpithet: enavata; scientificNameAuthorship: (Cresson, 1872); **Location:** country: United States; stateProvince: Mississippi; county: Sunflower; locality: Heathman Plantation, Holly Ridge; decimalLatitude: 33.462079; decimalLongitude: -90.707222; geodeticDatum: WGS1984; **Identification:** identifiedBy: K. A. Parys; dateIdentified: 2017; **Event:** samplingProtocol: Vane Trap (Blue); eventDate: 2016-6-21; **Record Level:** type: PhysicalObject; language: en; rights: https://creativecommons.org/publicdomain/zero/1.0/; accessRights: http://vertnet.org/resources/norms.html; institutionCode: USDA-ARS; collectionCode: SIMRU; basisOfRecord: PreservedSpecimen
- **Type status:** Other material. **Occurrence:** catalogNumber: SIMRU6836; recordedBy: K. A. Parys; individualCount: 1; sex: female; lifeStage: adult; preparations: pin; occurrenceID: urn:USDA-ARS:SIMRU:SIMRU6836; **Taxon:** scientificName: Diadasia (Diadasia) enavata (Cresson, 1872); kingdom: Animalia; phylum: Arthropoda; class: Insecta; order: Hymenoptera; family: Apidae; genus: Diadasia; subgenus: Diadasia; specificEpithet: enavata; scientificNameAuthorship: (Cresson, 1872); **Location:** country: United States; stateProvince: Mississippi; county: Sunflower; locality: Heathman Plantation, Holly Ridge; decimalLatitude: 33.462079; decimalLongitude: -90.707222; geodeticDatum: WGS1984; **Identification:** identifiedBy: K. A. Parys; dateIdentified: 2017; **Event:** samplingProtocol: Vane Trap (Blue); eventDate: 2016-6-21; **Record Level:** type: PhysicalObject; language: en; rights: https://creativecommons.org/publicdomain/zero/1.0/; accessRights: http://vertnet.org/resources/norms.html; institutionCode: USDA-ARS; collectionCode: SIMRU; basisOfRecord: PreservedSpecimen
- **Type status:** Other material. **Occurrence:** catalogNumber: SIMRU7858; recordedBy: K. A. Parys; individualCount: 1; sex: male; lifeStage: adult; preparations: pin; occurrenceID: urn:USDA-ARS:SIMRU:SIMRU7858; **Taxon:** scientificName: Diadasia (Diadasia) enavata (Cresson, 1872); kingdom: Animalia; phylum: Arthropoda; class: Insecta; order: Hymenoptera; family: Apidae; genus: Diadasia; subgenus: Diadasia; specificEpithet: enavata; scientificNameAuthorship: (Cresson, 1872); **Location:** country: United States; stateProvince: Mississippi; county: Sunflower; locality: Heathman Plantation, Holly Ridge; decimalLatitude: 33.462079; decimalLongitude: -90.707222; geodeticDatum: WGS1984; **Identification:** identifiedBy: T. Griswold; dateIdentified: 2017; **Event:** samplingProtocol: Bee Bowl (Blue); eventDate: 2016-7-19; **Record Level:** type: PhysicalObject; language: en; rights: https://creativecommons.org/publicdomain/zero/1.0/; accessRights: http://vertnet.org/resources/norms.html; institutionCode: USDA-ARS; collectionCode: SIMRU; basisOfRecord: PreservedSpecimen
- **Type status:** Other material. **Occurrence:** catalogNumber: SIMRU7859; recordedBy: K. A. Parys; individualCount: 1; sex: female; lifeStage: adult; preparations: pin; occurrenceID: urn:USDA-ARS:BBSL:SIMRU7859; **Taxon:** scientificName: Diadasia (Diadasia) enavata (Cresson, 1872); kingdom: Animalia; phylum: Arthropoda; class: Insecta; order: Hymenoptera; family: Apidae; genus: Diadasia; subgenus: Diadasia; specificEpithet: enavata; scientificNameAuthorship: (Cresson, 1872); **Location:** country: United States; stateProvince: Mississippi; county: Bolivar; locality: Alcorn Research Farm, Mound Bayou; decimalLatitude: 33.871265; decimalLongitude: -90.699184; geodeticDatum: WGS1984; **Identification:** identifiedBy: T. Griswold; dateIdentified: 2017; **Event:** samplingProtocol: Bee Bowl (Blue); eventDate: 2016-7-19; **Record Level:** type: PhysicalObject; language: en; rights: https://creativecommons.org/publicdomain/zero/1.0/; accessRights: http://vertnet.org/resources/norms.html; institutionCode: USDA-ARS; collectionCode: BBSL; basisOfRecord: PreservedSpecimen
- **Type status:** Other material. **Occurrence:** catalogNumber: SIMRU8956; recordedBy: K. A. Parys; individualCount: 1; sex: female; lifeStage: adult; preparations: pin; occurrenceID: urn:USDA-ARS:SIMRU:SIMRU8956; **Taxon:** scientificName: Diadasia (Diadasia) enavata (Cresson, 1872); kingdom: Animalia; phylum: Arthropoda; class: Insecta; order: Hymenoptera; family: Apidae; genus: Diadasia; subgenus: Diadasia; specificEpithet: enavata; scientificNameAuthorship: (Cresson, 1872); **Location:** country: United States; stateProvince: Mississippi; county: Bolivar; locality: Alcorn Research Farm, Mound Bayou; decimalLatitude: 33.871265; decimalLongitude: -90.699184; geodeticDatum: WGS1984; **Identification:** identifiedBy: T. Griswold; dateIdentified: 2017; **Event:** samplingProtocol: Vane Trap (Blue); eventDate: 2016-7-8; **Record Level:** type: PhysicalObject; language: en; rights: https://creativecommons.org/publicdomain/zero/1.0/; accessRights: http://vertnet.org/resources/norms.html; institutionCode: USDA-ARS; collectionCode: SIMRU; basisOfRecord: PreservedSpecimen
- **Type status:** Other material. **Occurrence:** catalogNumber: SIMRU9551; recordedBy: K. A. Parys; individualCount: 1; sex: female; lifeStage: adult; preparations: pin; occurrenceID: urn:USDA-ARS:SIMRU:SIMRU9551; **Taxon:** scientificName: Diadasia (Diadasia) enavata (Cresson, 1872); kingdom: Animalia; phylum: Arthropoda; class: Insecta; order: Hymenoptera; family: Apidae; genus: Diadasia; subgenus: Diadasia; specificEpithet: enavata; scientificNameAuthorship: (Cresson, 1872); **Location:** country: United States; stateProvince: Mississippi; county: Bolivar; locality: Alcorn Research Farm, Mound Bayou; decimalLatitude: 33.871265; decimalLongitude: -90.699184; geodeticDatum: WGS1984; **Identification:** identifiedBy: T. Griswold; dateIdentified: 2017; **Event:** samplingProtocol: Bee Bowl (Blue); eventDate: 2016-7-13; **Record Level:** type: PhysicalObject; language: en; rights: https://creativecommons.org/publicdomain/zero/1.0/; accessRights: http://vertnet.org/resources/norms.html; institutionCode: USDA-ARS; collectionCode: SIMRU; basisOfRecord: PreservedSpecimen
- **Type status:** Other material. **Occurrence:** catalogNumber: SIMRU9841; recordedBy: K. A. Parys; individualCount: 1; sex: female; lifeStage: adult; preparations: pin; occurrenceID: urn:USDA-ARS:SIMRU:SIMRU9841; **Taxon:** scientificName: Diadasia (Diadasia) enavata (Cresson, 1872); kingdom: Animalia; phylum: Arthropoda; class: Insecta; order: Hymenoptera; family: Apidae; genus: Diadasia; subgenus: Diadasia; specificEpithet: enavata; scientificNameAuthorship: (Cresson, 1872); **Location:** country: United States; stateProvince: Mississippi; county: Bolivar; locality: Alcorn Research Farm, Mound Bayou; decimalLatitude: 33.871265; decimalLongitude: -90.699184; geodeticDatum: WGS1984; **Identification:** identifiedBy: T. Griswold; dateIdentified: 2017; **Event:** samplingProtocol: Vane Trap (Blue); eventDate: 2016-8-10; **Record Level:** type: PhysicalObject; language: en; rights: https://creativecommons.org/publicdomain/zero/1.0/; accessRights: http://vertnet.org/resources/norms.html; institutionCode: USDA-ARS; collectionCode: SIMRU; basisOfRecord: PreservedSpecimen
- **Type status:** Other material. **Occurrence:** catalogNumber: SIMRU9930; recordedBy: K. A. Parys; individualCount: 1; sex: male; lifeStage: adult; preparations: pin; occurrenceID: urn:USDA-ARS:BBSL:SIMRU9930; **Taxon:** scientificName: Diadasia (Diadasia) enavata (Cresson, 1872); kingdom: Animalia; phylum: Arthropoda; class: Insecta; order: Hymenoptera; family: Apidae; genus: Diadasia; subgenus: Diadasia; specificEpithet: enavata; scientificNameAuthorship: (Cresson, 1872); **Location:** country: United States; stateProvince: Mississippi; county: Washington; locality: USDA Research Farm; decimalLatitude: 33.449579; decimalLongitude: -90.873142; geodeticDatum: WGS1984; **Identification:** identifiedBy: T. Griswold; dateIdentified: 2017; **Event:** samplingProtocol: Bee Bowl (Yellow); eventDate: 2016-8-10; **Record Level:** type: PhysicalObject; language: en; rights: https://creativecommons.org/publicdomain/zero/1.0/; accessRights: http://vertnet.org/resources/norms.html; institutionCode: USDA-ARS; collectionCode: BBSL; basisOfRecord: PreservedSpecimen
- **Type status:** Other material. **Occurrence:** catalogNumber: SIMRU9950; recordedBy: K. A. Parys; individualCount: 1; sex: male; lifeStage: adult; preparations: pin; occurrenceID: urn:USDA-ARS:SIMRU:SIMRU9950; **Taxon:** scientificName: Diadasia (Diadasia) enavata (Cresson, 1872); kingdom: Animalia; phylum: Arthropoda; class: Insecta; order: Hymenoptera; family: Apidae; genus: Diadasia; subgenus: Diadasia; specificEpithet: enavata; scientificNameAuthorship: (Cresson, 1872); **Location:** country: United States; stateProvince: Mississippi; county: Sunflower; locality: Heathman Plantation, Holly Ridge; decimalLatitude: 22.469196; decimalLongitude: -90.707709; geodeticDatum: WGS1984; **Identification:** identifiedBy: T. Griswold; dateIdentified: 2017; **Event:** samplingProtocol: Bee Bowl (Blue); eventDate: 2016-8-10; **Record Level:** type: PhysicalObject; language: en; rights: https://creativecommons.org/publicdomain/zero/1.0/; accessRights: http://vertnet.org/resources/norms.html; institutionCode: USDA-ARS; collectionCode: SIMRU; basisOfRecord: PreservedSpecimen
- **Type status:** Other material. **Occurrence:** catalogNumber: SIMRU9960; recordedBy: K. A. Parys; individualCount: 1; sex: male; lifeStage: adult; preparations: pin; occurrenceID: urn:USDA-ARS:SIMRU:SIMRU9960; **Taxon:** scientificName: Diadasia (Diadasia) enavata (Cresson, 1872); kingdom: Animalia; phylum: Arthropoda; class: Insecta; order: Hymenoptera; family: Apidae; genus: Diadasia; subgenus: Diadasia; specificEpithet: enavata; scientificNameAuthorship: (Cresson, 1872); **Location:** country: United States; stateProvince: Mississippi; county: Sunflower; locality: Heathman Plantation, Holly Ridge; decimalLatitude: 22.469196; decimalLongitude: -90.707709; geodeticDatum: WGS1984; **Identification:** identifiedBy: T. Griswold; dateIdentified: 2017; **Event:** samplingProtocol: Bee Bowl (White); eventDate: 2016-8-10; **Record Level:** type: PhysicalObject; language: en; rights: https://creativecommons.org/publicdomain/zero/1.0/; accessRights: http://vertnet.org/resources/norms.html; institutionCode: USDA-ARS; collectionCode: SIMRU; basisOfRecord: PreservedSpecimen
- **Type status:** Other material. **Occurrence:** catalogNumber: SIMRU10543; recordedBy: K. A. Parys; individualCount: 1; sex: female; lifeStage: adult; preparations: pin; occurrenceID: urn:USDA-ARS:BBSL:SIMRU10543; **Taxon:** scientificName: Diadasia (Diadasia) enavata (Cresson, 1872); kingdom: Animalia; phylum: Arthropoda; class: Insecta; order: Hymenoptera; family: Apidae; genus: Diadasia; subgenus: Diadasia; specificEpithet: enavata; scientificNameAuthorship: (Cresson, 1872); **Location:** country: United States; stateProvince: Mississippi; county: Sunflower; locality: Heathman Plantation, Holly Ridge; decimalLatitude: 22.469196; decimalLongitude: -90.707709; geodeticDatum: WGS1984; **Identification:** identifiedBy: T. Griswold; dateIdentified: 2017; **Event:** samplingProtocol: Bee Bowl (Blue); eventDate: 2016-8-10; **Record Level:** type: PhysicalObject; language: en; rights: https://creativecommons.org/publicdomain/zero/1.0/; accessRights: http://vertnet.org/resources/norms.html; institutionCode: USDA-ARS; collectionCode: BBSL; basisOfRecord: PreservedSpecimen
- **Type status:** Other material. **Occurrence:** catalogNumber: SIMRU10770; recordedBy: K. A. Parys; individualCount: 1; sex: male; lifeStage: adult; preparations: pin; occurrenceID: urn:USDA-ARS:SIMRU:SIMRU10770; **Taxon:** scientificName: Diadasia (Diadasia) enavata (Cresson, 1872); kingdom: Animalia; phylum: Arthropoda; class: Insecta; order: Hymenoptera; family: Apidae; genus: Diadasia; subgenus: Diadasia; specificEpithet: enavata; scientificNameAuthorship: (Cresson, 1872); **Location:** country: United States; stateProvince: Mississippi; county: Sunflower; locality: Heathman Plantation, Holly Ridge; decimalLatitude: 22.469196; decimalLongitude: -90.707709; geodeticDatum: WGS1984; **Identification:** identifiedBy: T. Griswold; dateIdentified: 2017; **Event:** samplingProtocol: Vane Trap (Blue); eventDate: 2016-8-24; **Record Level:** type: PhysicalObject; language: en; rights: https://creativecommons.org/publicdomain/zero/1.0/; accessRights: http://vertnet.org/resources/norms.html; institutionCode: USDA-ARS; collectionCode: SIMRU; basisOfRecord: PreservedSpecimen
- **Type status:** Other material. **Occurrence:** catalogNumber: SIMRU10772; recordedBy: K. A. Parys; individualCount: 1; sex: male; lifeStage: adult; preparations: pin; occurrenceID: urn:USDA-ARS:SIMRU:SIMRU10772; **Taxon:** scientificName: Diadasia (Diadasia) enavata (Cresson, 1872); kingdom: Animalia; phylum: Arthropoda; class: Insecta; order: Hymenoptera; family: Apidae; genus: Diadasia; subgenus: Diadasia; specificEpithet: enavata; scientificNameAuthorship: (Cresson, 1872); **Location:** country: United States; stateProvince: Mississippi; county: Sunflower; locality: Heathman Plantation, Holly Ridge; decimalLatitude: 22.469196; decimalLongitude: -90.707709; geodeticDatum: WGS1984; **Identification:** identifiedBy: T. Griswold; dateIdentified: 2017; **Event:** samplingProtocol: Vane Trap (Blue); eventDate: 2016-8-24; **Record Level:** type: PhysicalObject; language: en; rights: https://creativecommons.org/publicdomain/zero/1.0/; accessRights: http://vertnet.org/resources/norms.html; institutionCode: USDA-ARS; collectionCode: SIMRU; basisOfRecord: PreservedSpecimen
- **Type status:** Other material. **Occurrence:** catalogNumber: SIMRU10774; recordedBy: K. A. Parys; individualCount: 1; sex: male; lifeStage: adult; preparations: pin; occurrenceID: urn:USDA-ARS:SIMRU:SIMRU10774; **Taxon:** scientificName: Diadasia (Diadasia) enavata (Cresson, 1872); kingdom: Animalia; phylum: Arthropoda; class: Insecta; order: Hymenoptera; family: Apidae; genus: Diadasia; subgenus: Diadasia; specificEpithet: enavata; scientificNameAuthorship: (Cresson, 1872); **Location:** country: United States; stateProvince: Mississippi; county: Sunflower; locality: Heathman Plantation, Holly Ridge; decimalLatitude: 22.469196; decimalLongitude: -90.707709; geodeticDatum: WGS1984; **Identification:** identifiedBy: T. Griswold; dateIdentified: 2017; **Event:** samplingProtocol: Vane Trap (Blue); eventDate: 2016-8-24; **Record Level:** type: PhysicalObject; language: en; rights: https://creativecommons.org/publicdomain/zero/1.0/; accessRights: http://vertnet.org/resources/norms.html; institutionCode: USDA-ARS; collectionCode: SIMRU; basisOfRecord: PreservedSpecimen
- **Type status:** Other material. **Occurrence:** catalogNumber: SIMRU10778; recordedBy: K. A. Parys; individualCount: 1; sex: male; lifeStage: adult; preparations: pin; occurrenceID: urn:USDA-ARS:SIMRU:SIMRU10778; **Taxon:** scientificName: Diadasia (Diadasia) enavata (Cresson, 1872); kingdom: Animalia; phylum: Arthropoda; class: Insecta; order: Hymenoptera; family: Apidae; genus: Diadasia; subgenus: Diadasia; specificEpithet: enavata; scientificNameAuthorship: (Cresson, 1872); **Location:** country: United States; stateProvince: Mississippi; county: Sunflower; locality: Heathman Plantation, Holly Ridge; decimalLatitude: 22.469196; decimalLongitude: -90.707709; geodeticDatum: WGS1984; **Identification:** identifiedBy: T. Griswold; dateIdentified: 2017; **Event:** samplingProtocol: Vane Trap (Blue); eventDate: 2016-8-24; **Record Level:** type: PhysicalObject; language: en; rights: https://creativecommons.org/publicdomain/zero/1.0/; accessRights: http://vertnet.org/resources/norms.html; institutionCode: USDA-ARS; collectionCode: SIMRU; basisOfRecord: PreservedSpecimen
- **Type status:** Other material. **Occurrence:** catalogNumber: SIMRU11748; recordedBy: K. A. Parys; individualCount: 1; sex: female; lifeStage: adult; preparations: pin; occurrenceID: urn:USDA-ARS:SIMRU:SIMRU11748; **Taxon:** scientificName: Diadasia (Diadasia) enavata (Cresson, 1872); kingdom: Animalia; phylum: Arthropoda; class: Insecta; order: Hymenoptera; family: Apidae; genus: Diadasia; subgenus: Diadasia; specificEpithet: enavata; scientificNameAuthorship: (Cresson, 1872); **Location:** country: United States; stateProvince: Mississippi; county: Sunflower; locality: Heathman Plantation, Holly Ridge; decimalLatitude: 22.469196; decimalLongitude: -90.707709; geodeticDatum: WGS1984; **Identification:** identifiedBy: T. Griswold; dateIdentified: 2017; **Event:** samplingProtocol: Vane Trap (Blue); eventDate: 2016-8-4; **Record Level:** type: PhysicalObject; language: en; rights: https://creativecommons.org/publicdomain/zero/1.0/; accessRights: http://vertnet.org/resources/norms.html; institutionCode: USDA-ARS; collectionCode: SIMRU; basisOfRecord: PreservedSpecimen
- **Type status:** Other material. **Occurrence:** catalogNumber: SIMRU12459; recordedBy: K. A. Parys; individualCount: 1; sex: female; lifeStage: adult; preparations: pin; occurrenceID: urn:USDA-ARS:SIMRU:SIMRU12459; **Taxon:** scientificName: Diadasia (Diadasia) enavata (Cresson, 1872); kingdom: Animalia; phylum: Arthropoda; class: Insecta; order: Hymenoptera; family: Apidae; genus: Diadasia; subgenus: Diadasia; specificEpithet: enavata; scientificNameAuthorship: (Cresson, 1872); **Location:** country: United States; stateProvince: Mississippi; county: Sunflower; locality: Heathman Plantation, Holly Ridge; decimalLatitude: 22.469196; decimalLongitude: -90.707709; geodeticDatum: WGS1984; **Identification:** identifiedBy: T. Griswold; dateIdentified: 2017; **Event:** samplingProtocol: Bee Bowl (Blue); eventDate: 2016-8-3; **Record Level:** type: PhysicalObject; language: en; rights: https://creativecommons.org/publicdomain/zero/1.0/; accessRights: http://vertnet.org/resources/norms.html; institutionCode: USDA-ARS; collectionCode: SIMRU; basisOfRecord: PreservedSpecimen
- **Type status:** Other material. **Occurrence:** catalogNumber: SIMRU14150; recordedBy: K. A. Parys; individualCount: 1; sex: female; lifeStage: adult; preparations: pin; occurrenceID: urn:USDA-ARS:SIMRU:SIMRU14150; **Taxon:** scientificName: Diadasia (Diadasia) enavata (Cresson, 1872); kingdom: Animalia; phylum: Arthropoda; class: Insecta; order: Hymenoptera; family: Apidae; genus: Diadasia; subgenus: Diadasia; specificEpithet: enavata; scientificNameAuthorship: (Cresson, 1872); **Location:** country: United States; stateProvince: Mississippi; county: Sunflower; locality: Heathman Plantation, Holly Ridge; decimalLatitude: 22.469196; decimalLongitude: -90.707709; geodeticDatum: WGS1984; **Identification:** identifiedBy: T. Griswold; dateIdentified: 2017; **Event:** samplingProtocol: netting; eventDate: 2017-6-9; **Record Level:** type: PhysicalObject; language: en; rights: https://creativecommons.org/publicdomain/zero/1.0/; accessRights: http://vertnet.org/resources/norms.html; institutionCode: USDA-ARS; collectionCode: SIMRU; basisOfRecord: PreservedSpecimen
- **Type status:** Other material. **Occurrence:** catalogNumber: SIMRU18937; recordedBy: K. A. Parys; individualCount: 1; sex: female; lifeStage: adult; preparations: pin; occurrenceID: urn:USDA-ARS:SIMRU:SIMRU18937; **Taxon:** scientificName: Diadasia (Diadasia) enavata (Cresson, 1872); kingdom: Animalia; phylum: Arthropoda; class: Insecta; order: Hymenoptera; family: Apidae; genus: Diadasia; subgenus: Diadasia; specificEpithet: enavata; scientificNameAuthorship: (Cresson, 1872); **Location:** country: United States; stateProvince: Mississippi; county: Sunflower; locality: Heathman Plantation, Holly Ridge; decimalLatitude: 22.469196; decimalLongitude: -90.707709; geodeticDatum: WGS1984; **Identification:** identifiedBy: T. Griswold; dateIdentified: 2017; **Event:** samplingProtocol: Vane Trap (Blue); eventDate: 2017-7-25; **Record Level:** type: PhysicalObject; language: en; rights: https://creativecommons.org/publicdomain/zero/1.0/; accessRights: http://vertnet.org/resources/norms.html; institutionCode: USDA-ARS; collectionCode: SIMRU; basisOfRecord: PreservedSpecimen
- **Type status:** Other material. **Occurrence:** catalogNumber: SIMRU19801; recordedBy: K. A. Parys; individualCount: 1; sex: female; lifeStage: adult; preparations: pin; occurrenceID: urn:USDA-ARS:SIMRU:SIMRU19801; **Taxon:** scientificName: Diadasia (Diadasia) enavata (Cresson, 1872); kingdom: Animalia; phylum: Arthropoda; class: Insecta; order: Hymenoptera; family: Apidae; genus: Diadasia; subgenus: Diadasia; specificEpithet: enavata; scientificNameAuthorship: (Cresson, 1872); **Location:** country: United States; stateProvince: Mississippi; county: Sunflower; locality: Heathman Plantation, Holly Ridge; decimalLatitude: 22.469196; decimalLongitude: -90.707709; geodeticDatum: WGS1984; **Identification:** identifiedBy: T. Griswold; dateIdentified: 2017; **Event:** samplingProtocol: Bee Bowl (Blue); eventDate: 2017-6-21; **Record Level:** type: PhysicalObject; language: en; rights: https://creativecommons.org/publicdomain/zero/1.0/; accessRights: http://vertnet.org/resources/norms.html; institutionCode: USDA-ARS; collectionCode: SIMRU; basisOfRecord: PreservedSpecimen
- **Type status:** Other material. **Occurrence:** catalogNumber: SIMRU19802; recordedBy: K. A. Parys; individualCount: 1; sex: female; lifeStage: adult; preparations: pin; occurrenceID: urn:USDA-ARS:SIMRU:SIMRU19802; **Taxon:** scientificName: Diadasia (Diadasia) enavata (Cresson, 1872); kingdom: Animalia; phylum: Arthropoda; class: Insecta; order: Hymenoptera; family: Apidae; genus: Diadasia; subgenus: Diadasia; specificEpithet: enavata; scientificNameAuthorship: (Cresson, 1872); **Location:** country: United States; stateProvince: Mississippi; county: Sunflower; locality: Heathman Plantation, Holly Ridge; decimalLatitude: 22.469196; decimalLongitude: -90.707709; geodeticDatum: WGS1984; **Identification:** identifiedBy: T. Griswold; dateIdentified: 2017; **Event:** samplingProtocol: Bee Bowl (Blue); eventDate: 2017-6-21; **Record Level:** type: PhysicalObject; language: en; rights: https://creativecommons.org/publicdomain/zero/1.0/; accessRights: http://vertnet.org/resources/norms.html; institutionCode: USDA-ARS; collectionCode: SIMRU; basisOfRecord: PreservedSpecimen
- **Type status:** Other material. **Occurrence:** catalogNumber: SIMRU19807; recordedBy: K. A. Parys; individualCount: 1; sex: male; lifeStage: adult; preparations: pin; occurrenceID: urn:USDA-ARS:SIMRU:SIMRU19807; **Taxon:** scientificName: Diadasia (Diadasia) enavata (Cresson, 1872); kingdom: Animalia; phylum: Arthropoda; class: Insecta; order: Hymenoptera; family: Apidae; genus: Diadasia; subgenus: Diadasia; specificEpithet: enavata; scientificNameAuthorship: (Cresson, 1872); **Location:** country: United States; stateProvince: Mississippi; county: Sunflower; locality: Heathman Plantation, Holly Ridge; decimalLatitude: 22.469196; decimalLongitude: -90.707709; geodeticDatum: WGS1984; **Identification:** identifiedBy: T. Griswold; dateIdentified: 2017; **Event:** samplingProtocol: Bee Bowl (Blue); eventDate: 2017-6-21; **Record Level:** type: PhysicalObject; language: en; rights: https://creativecommons.org/publicdomain/zero/1.0/; accessRights: http://vertnet.org/resources/norms.html; institutionCode: USDA-ARS; collectionCode: SIMRU; basisOfRecord: PreservedSpecimen
- **Type status:** Other material. **Occurrence:** catalogNumber: SIMRU19832; recordedBy: K. A. Parys; individualCount: 1; sex: female; lifeStage: adult; preparations: pin; occurrenceID: urn:USDA-ARS:SIMRU:SIMRU19832; **Taxon:** scientificName: Diadasia (Diadasia) enavata (Cresson, 1872); kingdom: Animalia; phylum: Arthropoda; class: Insecta; order: Hymenoptera; family: Apidae; genus: Diadasia; subgenus: Diadasia; specificEpithet: enavata; scientificNameAuthorship: (Cresson, 1872); **Location:** country: United States; stateProvince: Mississippi; county: Sunflower; locality: Heathman Plantation, Holly Ridge; decimalLatitude: 22.469196; decimalLongitude: -90.707709; geodeticDatum: WGS1984; **Identification:** identifiedBy: T. Griswold; dateIdentified: 2017; **Event:** samplingProtocol: Bee Bowl (Blue); eventDate: 2017-6-21; **Record Level:** type: PhysicalObject; language: en; rights: https://creativecommons.org/publicdomain/zero/1.0/; accessRights: http://vertnet.org/resources/norms.html; institutionCode: USDA-ARS; collectionCode: SIMRU; basisOfRecord: PreservedSpecimen
- **Type status:** Other material. **Occurrence:** catalogNumber: SIMRU19833; recordedBy: K. A. Parys; individualCount: 1; sex: female; lifeStage: adult; preparations: pin; occurrenceID: urn:USDA-ARS:SIMRU:SIMRU19833; **Taxon:** scientificName: Diadasia (Diadasia) enavata (Cresson, 1872); kingdom: Animalia; phylum: Arthropoda; class: Insecta; order: Hymenoptera; family: Apidae; genus: Diadasia; subgenus: Diadasia; specificEpithet: enavata; scientificNameAuthorship: (Cresson, 1872); **Location:** country: United States; stateProvince: Mississippi; county: Sunflower; locality: Heathman Plantation, Holly Ridge; decimalLatitude: 22.469196; decimalLongitude: -90.707709; geodeticDatum: WGS1984; **Identification:** identifiedBy: T. Griswold; dateIdentified: 2017; **Event:** samplingProtocol: Bee Bowl (Blue); eventDate: 2017-6-21; **Record Level:** type: PhysicalObject; language: en; rights: https://creativecommons.org/publicdomain/zero/1.0/; accessRights: http://vertnet.org/resources/norms.html; institutionCode: USDA-ARS; collectionCode: SIMRU; basisOfRecord: PreservedSpecimen
- **Type status:** Other material. **Occurrence:** catalogNumber: SIMRU19996; recordedBy: K. A. Parys; individualCount: 1; sex: female; lifeStage: adult; preparations: pin; occurrenceID: urn:USDA-ARS:BBSL:SIMRU19996; **Taxon:** scientificName: Diadasia (Diadasia) enavata (Cresson, 1872); kingdom: Animalia; phylum: Arthropoda; class: Insecta; order: Hymenoptera; family: Apidae; genus: Diadasia; subgenus: Diadasia; specificEpithet: enavata; scientificNameAuthorship: (Cresson, 1872); **Location:** country: United States; stateProvince: Mississippi; county: Sunflower; locality: Heathman Plantation, Holly Ridge; decimalLatitude: 22.469196; decimalLongitude: -90.707709; geodeticDatum: WGS1984; **Identification:** identifiedBy: T. Griswold; dateIdentified: 2017; **Event:** samplingProtocol: Bee Bowl (Yellow); eventDate: 2017-7-25; **Record Level:** type: PhysicalObject; language: en; rights: https://creativecommons.org/publicdomain/zero/1.0/; accessRights: http://vertnet.org/resources/norms.html; institutionCode: USDA-ARS; collectionCode: BBSL; basisOfRecord: PreservedSpecimen
- **Type status:** Other material. **Occurrence:** catalogNumber: SIMRU20017; recordedBy: K. A. Parys; individualCount: 1; sex: male; lifeStage: adult; preparations: pin; occurrenceID: urn:USDA-ARS:SIMRU:SIMRU20017; **Taxon:** scientificName: Diadasia (Diadasia) enavata (Cresson, 1872); kingdom: Animalia; phylum: Arthropoda; class: Insecta; order: Hymenoptera; family: Apidae; genus: Diadasia; subgenus: Diadasia; specificEpithet: enavata; scientificNameAuthorship: (Cresson, 1872); **Location:** country: United States; stateProvince: Mississippi; county: Sunflower; locality: Heathman Plantation, Holly Ridge; decimalLatitude: 22.469196; decimalLongitude: -90.707709; geodeticDatum: WGS1984; **Identification:** identifiedBy: T. Griswold; dateIdentified: 2017; **Event:** samplingProtocol: Bee Bowl (Yellow); eventDate: 2017-7-25; **Record Level:** type: PhysicalObject; language: en; rights: https://creativecommons.org/publicdomain/zero/1.0/; accessRights: http://vertnet.org/resources/norms.html; institutionCode: USDA-ARS; collectionCode: SIMRU; basisOfRecord: PreservedSpecimen
- **Type status:** Other material. **Occurrence:** catalogNumber: SIMRU20033; recordedBy: K. A. Parys; individualCount: 1; sex: female; lifeStage: adult; preparations: pin; occurrenceID: urn:USDA-ARS:SIMRU:SIMRU20033; **Taxon:** scientificName: Diadasia (Diadasia) enavata (Cresson, 1872); kingdom: Animalia; phylum: Arthropoda; class: Insecta; order: Hymenoptera; family: Apidae; genus: Diadasia; subgenus: Diadasia; specificEpithet: enavata; scientificNameAuthorship: (Cresson, 1872); **Location:** country: United States; stateProvince: Mississippi; county: Sunflower; locality: Heathman Plantation, Holly Ridge; decimalLatitude: 22.469196; decimalLongitude: -90.707709; geodeticDatum: WGS1984; **Identification:** identifiedBy: T. Griswold; dateIdentified: 2017; **Event:** samplingProtocol: Bee Bowl (Blue); eventDate: 2017-7-25; **Record Level:** type: PhysicalObject; language: en; rights: https://creativecommons.org/publicdomain/zero/1.0/; accessRights: http://vertnet.org/resources/norms.html; institutionCode: USDA-ARS; collectionCode: SIMRU; basisOfRecord: PreservedSpecimen
- **Type status:** Other material. **Occurrence:** catalogNumber: SIMRU20499; recordedBy: K. A. Parys; individualCount: 1; sex: male; lifeStage: adult; preparations: pin; occurrenceID: urn:USDA-ARS:SIMRU:SIMRU20499; **Taxon:** scientificName: Diadasia (Diadasia) enavata (Cresson, 1872); kingdom: Animalia; phylum: Arthropoda; class: Insecta; order: Hymenoptera; family: Apidae; genus: Diadasia; subgenus: Diadasia; specificEpithet: enavata; scientificNameAuthorship: (Cresson, 1872); **Location:** country: United States; stateProvince: Mississippi; county: Sunflower; locality: Heathman Plantation, Holly Ridge; decimalLatitude: 22.469196; decimalLongitude: -90.707709; geodeticDatum: WGS1984; **Identification:** identifiedBy: T. Griswold; dateIdentified: 2017; **Event:** samplingProtocol: Vane Trap (Blue); eventDate: 2017-8-1; **Record Level:** type: PhysicalObject; language: en; rights: https://creativecommons.org/publicdomain/zero/1.0/; accessRights: http://vertnet.org/resources/norms.html; institutionCode: USDA-ARS; collectionCode: SIMRU; basisOfRecord: PreservedSpecimen
- **Type status:** Other material. **Occurrence:** catalogNumber: SIMRU20826; recordedBy: K. A. Parys; individualCount: 1; sex: male; lifeStage: adult; preparations: pin; occurrenceID: urn:USDA-ARS:BBSL:SIMRU20826; **Taxon:** scientificName: Diadasia (Diadasia) enavata (Cresson, 1872); kingdom: Animalia; phylum: Arthropoda; class: Insecta; order: Hymenoptera; family: Apidae; genus: Diadasia; subgenus: Diadasia; specificEpithet: enavata; scientificNameAuthorship: (Cresson, 1872); **Location:** country: United States; stateProvince: Mississippi; county: Sunflower; locality: Heathman Plantation, Holly Ridge; decimalLatitude: 22.469196; decimalLongitude: -90.707709; geodeticDatum: WGS1984; **Identification:** identifiedBy: T. Griswold; dateIdentified: 2017; **Event:** samplingProtocol: netting; eventDate: 2017-7-31; **Record Level:** type: PhysicalObject; language: en; rights: https://creativecommons.org/publicdomain/zero/1.0/; accessRights: http://vertnet.org/resources/norms.html; institutionCode: USDA-ARS; collectionCode: BBSL; basisOfRecord: PreservedSpecimen
- **Type status:** Other material. **Occurrence:** catalogNumber: SIMRU20827; recordedBy: K. A. Parys; individualCount: 1; sex: male; lifeStage: adult; preparations: pin; occurrenceID: urn:USDA-ARS:SIMRU:SIMRU20827; **Taxon:** scientificName: Diadasia (Diadasia) enavata (Cresson, 1872); kingdom: Animalia; phylum: Arthropoda; class: Insecta; order: Hymenoptera; family: Apidae; genus: Diadasia; subgenus: Diadasia; specificEpithet: enavata; scientificNameAuthorship: (Cresson, 1872); **Location:** country: United States; stateProvince: Mississippi; county: Sunflower; locality: Heathman Plantation, Holly Ridge; decimalLatitude: 22.469196; decimalLongitude: -90.707709; geodeticDatum: WGS1984; **Identification:** identifiedBy: T. Griswold; dateIdentified: 2017; **Event:** samplingProtocol: netting; eventDate: 2017-7-31; **Record Level:** type: PhysicalObject; language: en; rights: https://creativecommons.org/publicdomain/zero/1.0/; accessRights: http://vertnet.org/resources/norms.html; institutionCode: USDA-ARS; collectionCode: SIMRU; basisOfRecord: PreservedSpecimen
- **Type status:** Other material. **Occurrence:** catalogNumber: SIMRU20828; recordedBy: K. A. Parys; individualCount: 1; sex: male; lifeStage: adult; preparations: pin; occurrenceID: urn:USDA-ARS:SIMRU:SIMRU20828; **Taxon:** scientificName: Diadasia (Diadasia) enavata (Cresson, 1872); kingdom: Animalia; phylum: Arthropoda; class: Insecta; order: Hymenoptera; family: Apidae; genus: Diadasia; subgenus: Diadasia; specificEpithet: enavata; scientificNameAuthorship: (Cresson, 1872); **Location:** country: United States; stateProvince: Mississippi; county: Sunflower; locality: Heathman Plantation, Holly Ridge; decimalLatitude: 22.469196; decimalLongitude: -90.707709; geodeticDatum: WGS1984; **Identification:** identifiedBy: T. Griswold; dateIdentified: 2017; **Event:** samplingProtocol: netting; eventDate: 2017-7-31; **Record Level:** type: PhysicalObject; language: en; rights: https://creativecommons.org/publicdomain/zero/1.0/; accessRights: http://vertnet.org/resources/norms.html; institutionCode: USDA-ARS; collectionCode: SIMRU; basisOfRecord: PreservedSpecimen
- **Type status:** Other material. **Occurrence:** catalogNumber: SIMRU20885; recordedBy: K. A. Parys; individualCount: 1; sex: female; lifeStage: adult; preparations: pin; occurrenceID: urn:USDA-ARS:BBSL:SIMRU20885; **Taxon:** scientificName: Diadasia (Diadasia) enavata (Cresson, 1872); kingdom: Animalia; phylum: Arthropoda; class: Insecta; order: Hymenoptera; family: Apidae; genus: Diadasia; subgenus: Diadasia; specificEpithet: enavata; scientificNameAuthorship: (Cresson, 1872); **Location:** country: United States; stateProvince: Mississippi; county: Sunflower; locality: Heathman Plantation, Holly Ridge; decimalLatitude: 22.469196; decimalLongitude: -90.707709; geodeticDatum: WGS1984; **Identification:** identifiedBy: T. Griswold; dateIdentified: 2017; **Event:** samplingProtocol: Vane Trap (Blue); eventDate: 2017-8-8; **Record Level:** type: PhysicalObject; language: en; rights: https://creativecommons.org/publicdomain/zero/1.0/; accessRights: http://vertnet.org/resources/norms.html; institutionCode: USDA-ARS; collectionCode: BBSL; basisOfRecord: PreservedSpecimen
- **Type status:** Other material. **Occurrence:** catalogNumber: SIMRU21031; recordedBy: K. A. Parys; individualCount: 1; sex: male; lifeStage: adult; preparations: pin; occurrenceID: urn:USDA-ARS:SIMRU:SIMRU21031; **Taxon:** scientificName: Diadasia (Diadasia) enavata (Cresson, 1872); kingdom: Animalia; phylum: Arthropoda; class: Insecta; order: Hymenoptera; family: Apidae; genus: Diadasia; subgenus: Diadasia; specificEpithet: enavata; scientificNameAuthorship: (Cresson, 1872); **Location:** country: United States; stateProvince: Mississippi; county: Sunflower; locality: Heathman Plantation, Holly Ridge; decimalLatitude: 22.469196; decimalLongitude: -90.707709; geodeticDatum: WGS1984; **Identification:** identifiedBy: T. Griswold; dateIdentified: 2017; **Event:** samplingProtocol: Vane Trap (Blue); eventDate: 2017-8-9; **Record Level:** type: PhysicalObject; language: en; rights: https://creativecommons.org/publicdomain/zero/1.0/; accessRights: http://vertnet.org/resources/norms.html; institutionCode: USDA-ARS; collectionCode: SIMRU; basisOfRecord: PreservedSpecimen
- **Type status:** Other material. **Occurrence:** catalogNumber: SIMRU21038; recordedBy: K. A. Parys; individualCount: 1; sex: female; lifeStage: adult; preparations: pin; occurrenceID: urn:USDA-ARS:SIMRU:SIMRU21038; **Taxon:** scientificName: Diadasia (Diadasia) enavata (Cresson, 1872); kingdom: Animalia; phylum: Arthropoda; class: Insecta; order: Hymenoptera; family: Apidae; genus: Diadasia; subgenus: Diadasia; specificEpithet: enavata; scientificNameAuthorship: (Cresson, 1872); **Location:** country: United States; stateProvince: Mississippi; county: Sunflower; locality: Heathman Plantation, Holly Ridge; decimalLatitude: 22.469196; decimalLongitude: -90.707709; geodeticDatum: WGS1984; **Identification:** identifiedBy: T. Griswold; dateIdentified: 2017; **Event:** samplingProtocol: Vane Trap (Blue); eventDate: 2017-8-9; **Record Level:** type: PhysicalObject; language: en; rights: https://creativecommons.org/publicdomain/zero/1.0/; accessRights: http://vertnet.org/resources/norms.html; institutionCode: USDA-ARS; collectionCode: SIMRU; basisOfRecord: PreservedSpecimen
- **Type status:** Other material. **Occurrence:** catalogNumber: SIMRU21039; recordedBy: K. A. Parys; individualCount: 1; sex: male; lifeStage: adult; preparations: pin; occurrenceID: urn:USDA-ARS:SIMRU:SIMRU21039; **Taxon:** scientificName: Diadasia (Diadasia) enavata (Cresson, 1872); kingdom: Animalia; phylum: Arthropoda; class: Insecta; order: Hymenoptera; family: Apidae; genus: Diadasia; subgenus: Diadasia; specificEpithet: enavata; scientificNameAuthorship: (Cresson, 1872); **Location:** country: United States; stateProvince: Mississippi; county: Sunflower; locality: Heathman Plantation, Holly Ridge; decimalLatitude: 22.469196; decimalLongitude: -90.707709; geodeticDatum: WGS1984; **Identification:** identifiedBy: T. Griswold; dateIdentified: 2017; **Event:** samplingProtocol: Vane Trap (Blue); eventDate: 2017-8-9; **Record Level:** type: PhysicalObject; language: en; rights: https://creativecommons.org/publicdomain/zero/1.0/; accessRights: http://vertnet.org/resources/norms.html; institutionCode: USDA-ARS; collectionCode: SIMRU; basisOfRecord: PreservedSpecimen
- **Type status:** Other material. **Occurrence:** catalogNumber: SIMRU21066; recordedBy: K. A. Parys; individualCount: 1; sex: male; lifeStage: adult; preparations: pin; occurrenceID: urn:USDA-ARS:SIMRU:SIMRU21066; **Taxon:** scientificName: Diadasia (Diadasia) enavata (Cresson, 1872); kingdom: Animalia; phylum: Arthropoda; class: Insecta; order: Hymenoptera; family: Apidae; genus: Diadasia; subgenus: Diadasia; specificEpithet: enavata; scientificNameAuthorship: (Cresson, 1872); **Location:** country: United States; stateProvince: Mississippi; county: Sunflower; locality: Heathman Plantation, Holly Ridge; decimalLatitude: 22.469196; decimalLongitude: -90.707709; geodeticDatum: WGS1984; **Identification:** identifiedBy: T. Griswold; dateIdentified: 2017; **Event:** samplingProtocol: Vane Trap (Blue); eventDate: 2017-8-9; **Record Level:** type: PhysicalObject; language: en; rights: https://creativecommons.org/publicdomain/zero/1.0/; accessRights: http://vertnet.org/resources/norms.html; institutionCode: USDA-ARS; collectionCode: SIMRU; basisOfRecord: PreservedSpecimen
- **Type status:** Other material. **Occurrence:** catalogNumber: SIMRU21926; recordedBy: K. A. Parys; individualCount: 1; sex: female; lifeStage: adult; preparations: pin; occurrenceID: urn:USDA-ARS:SIMRU:SIMRU21926; **Taxon:** scientificName: Diadasia (Diadasia) enavata (Cresson, 1872); kingdom: Animalia; phylum: Arthropoda; class: Insecta; order: Hymenoptera; family: Apidae; genus: Diadasia; subgenus: Diadasia; specificEpithet: enavata; scientificNameAuthorship: (Cresson, 1872); **Location:** country: United States; stateProvince: Mississippi; county: Sunflower; locality: Heathman Plantation, Holly Ridge; decimalLatitude: 22.469196; decimalLongitude: -90.707709; geodeticDatum: WGS1984; **Identification:** identifiedBy: K. A. Parys; dateIdentified: 2017; **Event:** samplingProtocol: Bee Bowl (Yellow); eventDate: 2017-8-1; **Record Level:** type: PhysicalObject; language: en; rights: https://creativecommons.org/publicdomain/zero/1.0/; accessRights: http://vertnet.org/resources/norms.html; institutionCode: USDA-ARS; collectionCode: SIMRU; basisOfRecord: PreservedSpecimen
- **Type status:** Other material. **Occurrence:** catalogNumber: SIMRU21927; recordedBy: K. A. Parys; individualCount: 1; sex: female; lifeStage: adult; preparations: pin; occurrenceID: urn:USDA-ARS:SIMRU:SIMRU21927; **Taxon:** scientificName: Diadasia (Diadasia) enavata (Cresson, 1872); kingdom: Animalia; phylum: Arthropoda; class: Insecta; order: Hymenoptera; family: Apidae; genus: Diadasia; subgenus: Diadasia; specificEpithet: enavata; scientificNameAuthorship: (Cresson, 1872); **Location:** country: United States; stateProvince: Mississippi; county: Sunflower; locality: Heathman Plantation, Holly Ridge; decimalLatitude: 22.469196; decimalLongitude: -90.707709; geodeticDatum: WGS1984; **Identification:** identifiedBy: K. A. Parys; dateIdentified: 2017; **Event:** samplingProtocol: Bee Bowl (Yellow); eventDate: 2017-8-1; **Record Level:** type: PhysicalObject; language: en; rights: https://creativecommons.org/publicdomain/zero/1.0/; accessRights: http://vertnet.org/resources/norms.html; institutionCode: USDA-ARS; collectionCode: SIMRU; basisOfRecord: PreservedSpecimen
- **Type status:** Other material. **Occurrence:** catalogNumber: SIMRU21929; recordedBy: K. A. Parys; individualCount: 1; sex: female; lifeStage: adult; preparations: pin; occurrenceID: urn:USDA-ARS:SIMRU:SIMRU21929; **Taxon:** scientificName: Diadasia (Diadasia) enavata (Cresson, 1872); kingdom: Animalia; phylum: Arthropoda; class: Insecta; order: Hymenoptera; family: Apidae; genus: Diadasia; subgenus: Diadasia; specificEpithet: enavata; scientificNameAuthorship: (Cresson, 1872); **Location:** country: United States; stateProvince: Mississippi; county: Sunflower; locality: Heathman Plantation, Holly Ridge; decimalLatitude: 22.469196; decimalLongitude: -90.707709; geodeticDatum: WGS1984; **Identification:** identifiedBy: K. A. Parys; dateIdentified: 2017; **Event:** samplingProtocol: Bee Bowl (Yellow); eventDate: 2017-8-1; **Record Level:** type: PhysicalObject; language: en; rights: https://creativecommons.org/publicdomain/zero/1.0/; accessRights: http://vertnet.org/resources/norms.html; institutionCode: USDA-ARS; collectionCode: SIMRU; basisOfRecord: PreservedSpecimen
- **Type status:** Other material. **Occurrence:** catalogNumber: SIMRU21941; recordedBy: K. A. Parys; individualCount: 1; sex: female; lifeStage: adult; preparations: pin; occurrenceID: urn:USDA-ARS:SIMRU:SIMRU21941; **Taxon:** scientificName: Diadasia (Diadasia) enavata (Cresson, 1872); kingdom: Animalia; phylum: Arthropoda; class: Insecta; order: Hymenoptera; family: Apidae; genus: Diadasia; subgenus: Diadasia; specificEpithet: enavata; scientificNameAuthorship: (Cresson, 1872); **Location:** country: United States; stateProvince: Mississippi; county: Sunflower; locality: Heathman Plantation, Holly Ridge; decimalLatitude: 22.469196; decimalLongitude: -90.707709; geodeticDatum: WGS1984; **Identification:** identifiedBy: K. A. Parys; dateIdentified: 2017; **Event:** samplingProtocol: Bee Bowl (Yellow); eventDate: 2017-8-1; **Record Level:** type: PhysicalObject; language: en; rights: https://creativecommons.org/publicdomain/zero/1.0/; accessRights: http://vertnet.org/resources/norms.html; institutionCode: USDA-ARS; collectionCode: SIMRU; basisOfRecord: PreservedSpecimen
- **Type status:** Other material. **Occurrence:** catalogNumber: SIMRU21942; recordedBy: K. A. Parys; individualCount: 1; sex: female; lifeStage: adult; preparations: pin; occurrenceID: urn:USDA-ARS:SIMRU:SIMRU21942; **Taxon:** scientificName: Diadasia (Diadasia) enavata (Cresson, 1872); kingdom: Animalia; phylum: Arthropoda; class: Insecta; order: Hymenoptera; family: Apidae; genus: Diadasia; subgenus: Diadasia; specificEpithet: enavata; scientificNameAuthorship: (Cresson, 1872); **Location:** country: United States; stateProvince: Mississippi; county: Sunflower; locality: Heathman Plantation, Holly Ridge; decimalLatitude: 22.469196; decimalLongitude: -90.707709; geodeticDatum: WGS1984; **Identification:** identifiedBy: K. A. Parys; dateIdentified: 2017; **Event:** samplingProtocol: Bee Bowl (Yellow); eventDate: 2017-8-1; **Record Level:** type: PhysicalObject; language: en; rights: https://creativecommons.org/publicdomain/zero/1.0/; accessRights: http://vertnet.org/resources/norms.html; institutionCode: USDA-ARS; collectionCode: SIMRU; basisOfRecord: PreservedSpecimen
- **Type status:** Other material. **Occurrence:** catalogNumber: SIMRU21943; recordedBy: K. A. Parys; individualCount: 1; sex: female; lifeStage: adult; preparations: pin; occurrenceID: urn:USDA-ARS:SIMRU:SIMRU21943; **Taxon:** scientificName: Diadasia (Diadasia) enavata (Cresson, 1872); kingdom: Animalia; phylum: Arthropoda; class: Insecta; order: Hymenoptera; family: Apidae; genus: Diadasia; subgenus: Diadasia; specificEpithet: enavata; scientificNameAuthorship: (Cresson, 1872); **Location:** country: United States; stateProvince: Mississippi; county: Sunflower; locality: Heathman Plantation, Holly Ridge; decimalLatitude: 22.469196; decimalLongitude: -90.707709; geodeticDatum: WGS1984; **Identification:** identifiedBy: K. A. Parys; dateIdentified: 2017; **Event:** samplingProtocol: Bee Bowl (Yellow); eventDate: 2017-8-1; **Record Level:** type: PhysicalObject; language: en; rights: https://creativecommons.org/publicdomain/zero/1.0/; accessRights: http://vertnet.org/resources/norms.html; institutionCode: USDA-ARS; collectionCode: SIMRU; basisOfRecord: PreservedSpecimen
- **Type status:** Other material. **Occurrence:** catalogNumber: SIMRU21947; recordedBy: K. A. Parys; individualCount: 1; sex: female; lifeStage: adult; preparations: pin; occurrenceID: urn:USDA-ARS:SIMRU:SIMRU21947; **Taxon:** scientificName: Diadasia (Diadasia) enavata (Cresson, 1872); kingdom: Animalia; phylum: Arthropoda; class: Insecta; order: Hymenoptera; family: Apidae; genus: Diadasia; subgenus: Diadasia; specificEpithet: enavata; scientificNameAuthorship: (Cresson, 1872); **Location:** country: United States; stateProvince: Mississippi; county: Sunflower; locality: Heathman Plantation, Holly Ridge; decimalLatitude: 22.469196; decimalLongitude: -90.707709; geodeticDatum: WGS1984; **Identification:** identifiedBy: K. A. Parys; dateIdentified: 2017; **Event:** samplingProtocol: Bee Bowl (Yellow); eventDate: 2017-8-1; **Record Level:** type: PhysicalObject; language: en; rights: https://creativecommons.org/publicdomain/zero/1.0/; accessRights: http://vertnet.org/resources/norms.html; institutionCode: USDA-ARS; collectionCode: SIMRU; basisOfRecord: PreservedSpecimen
- **Type status:** Other material. **Occurrence:** catalogNumber: SIMRU21951; recordedBy: K. A. Parys; individualCount: 1; sex: female; lifeStage: adult; preparations: pin; occurrenceID: urn:USDA-ARS:SIMRU:SIMRU21951; **Taxon:** scientificName: Diadasia (Diadasia) enavata (Cresson, 1872); kingdom: Animalia; phylum: Arthropoda; class: Insecta; order: Hymenoptera; family: Apidae; genus: Diadasia; subgenus: Diadasia; specificEpithet: enavata; scientificNameAuthorship: (Cresson, 1872); **Location:** country: United States; stateProvince: Mississippi; county: Sunflower; locality: Heathman Plantation, Holly Ridge; decimalLatitude: 22.469196; decimalLongitude: -90.707709; geodeticDatum: WGS1984; **Identification:** identifiedBy: K. A. Parys; dateIdentified: 2017; **Event:** samplingProtocol: Bee Bowl (Yellow); eventDate: 2017-8-1; **Record Level:** type: PhysicalObject; language: en; rights: https://creativecommons.org/publicdomain/zero/1.0/; accessRights: http://vertnet.org/resources/norms.html; institutionCode: USDA-ARS; collectionCode: SIMRU; basisOfRecord: PreservedSpecimen
- **Type status:** Other material. **Occurrence:** catalogNumber: SIMRU21959; recordedBy: K. A. Parys; individualCount: 1; sex: female; lifeStage: adult; preparations: pin; occurrenceID: urn:USDA-ARS:SIMRU:SIMRU21959; **Taxon:** scientificName: Diadasia (Diadasia) enavata (Cresson, 1872); kingdom: Animalia; phylum: Arthropoda; class: Insecta; order: Hymenoptera; family: Apidae; genus: Diadasia; subgenus: Diadasia; specificEpithet: enavata; scientificNameAuthorship: (Cresson, 1872); **Location:** country: United States; stateProvince: Mississippi; county: Sunflower; locality: Heathman Plantation, Holly Ridge; decimalLatitude: 22.469196; decimalLongitude: -90.707709; geodeticDatum: WGS1984; **Identification:** identifiedBy: K. A. Parys; dateIdentified: 2017; **Event:** samplingProtocol: Bee Bowl (Yellow); eventDate: 2017-8-1; **Record Level:** type: PhysicalObject; language: en; rights: https://creativecommons.org/publicdomain/zero/1.0/; accessRights: http://vertnet.org/resources/norms.html; institutionCode: USDA-ARS; collectionCode: SIMRU; basisOfRecord: PreservedSpecimen
- **Type status:** Other material. **Occurrence:** catalogNumber: SIMRU21960; recordedBy: K. A. Parys; individualCount: 1; sex: female; lifeStage: adult; preparations: pin; occurrenceID: urn:USDA-ARS:SIMRU:SIMRU21960; **Taxon:** scientificName: Diadasia (Diadasia) enavata (Cresson, 1872); kingdom: Animalia; phylum: Arthropoda; class: Insecta; order: Hymenoptera; family: Apidae; genus: Diadasia; subgenus: Diadasia; specificEpithet: enavata; scientificNameAuthorship: (Cresson, 1872); **Location:** country: United States; stateProvince: Mississippi; county: Sunflower; locality: Heathman Plantation, Holly Ridge; decimalLatitude: 22.469196; decimalLongitude: -90.707709; geodeticDatum: WGS1984; **Identification:** identifiedBy: K. A. Parys; dateIdentified: 2017; **Event:** samplingProtocol: Bee Bowl (Yellow); eventDate: 2017-8-1; **Record Level:** type: PhysicalObject; language: en; rights: https://creativecommons.org/publicdomain/zero/1.0/; accessRights: http://vertnet.org/resources/norms.html; institutionCode: USDA-ARS; collectionCode: SIMRU; basisOfRecord: PreservedSpecimen
- **Type status:** Other material. **Occurrence:** catalogNumber: SIMRU21962; recordedBy: K. A. Parys; individualCount: 1; sex: female; lifeStage: adult; preparations: pin; occurrenceID: urn:USDA-ARS:SIMRU:SIMRU21962; **Taxon:** scientificName: Diadasia (Diadasia) enavata (Cresson, 1872); kingdom: Animalia; phylum: Arthropoda; class: Insecta; order: Hymenoptera; family: Apidae; genus: Diadasia; subgenus: Diadasia; specificEpithet: enavata; scientificNameAuthorship: (Cresson, 1872); **Location:** country: United States; stateProvince: Mississippi; county: Sunflower; locality: Heathman Plantation, Holly Ridge; decimalLatitude: 22.469196; decimalLongitude: -90.707709; geodeticDatum: WGS1984; **Identification:** identifiedBy: K. A. Parys; dateIdentified: 2017; **Event:** samplingProtocol: Bee Bowl (Yellow); eventDate: 2017-8-1; **Record Level:** type: PhysicalObject; language: en; rights: https://creativecommons.org/publicdomain/zero/1.0/; accessRights: http://vertnet.org/resources/norms.html; institutionCode: USDA-ARS; collectionCode: SIMRU; basisOfRecord: PreservedSpecimen
- **Type status:** Other material. **Occurrence:** catalogNumber: SIMRU21963; recordedBy: K. A. Parys; individualCount: 1; sex: male; lifeStage: adult; preparations: pin; occurrenceID: urn:USDA-ARS:SIMRU:SIMRU21963; **Taxon:** scientificName: Diadasia (Diadasia) enavata (Cresson, 1872); kingdom: Animalia; phylum: Arthropoda; class: Insecta; order: Hymenoptera; family: Apidae; genus: Diadasia; subgenus: Diadasia; specificEpithet: enavata; scientificNameAuthorship: (Cresson, 1872); **Location:** country: United States; stateProvince: Mississippi; county: Sunflower; locality: Heathman Plantation, Holly Ridge; decimalLatitude: 33.462079; decimalLongitude: -90.707222; geodeticDatum: WGS1984; **Identification:** identifiedBy: K. A. Parys; dateIdentified: 2017; **Event:** samplingProtocol: Bee Bowl (Yellow); eventDate: 2017-8-1; **Record Level:** type: PhysicalObject; language: en; rights: https://creativecommons.org/publicdomain/zero/1.0/; accessRights: http://vertnet.org/resources/norms.html; institutionCode: USDA-ARS; collectionCode: SIMRU; basisOfRecord: PreservedSpecimen
- **Type status:** Other material. **Occurrence:** catalogNumber: SIMRU21964; recordedBy: K. A. Parys; individualCount: 1; sex: female; lifeStage: adult; preparations: pin; occurrenceID: urn:USDA-ARS:SIMRU:SIMRU21964; **Taxon:** scientificName: Diadasia (Diadasia) enavata (Cresson, 1872); kingdom: Animalia; phylum: Arthropoda; class: Insecta; order: Hymenoptera; family: Apidae; genus: Diadasia; subgenus: Diadasia; specificEpithet: enavata; scientificNameAuthorship: (Cresson, 1872); **Location:** country: United States; stateProvince: Mississippi; county: Sunflower; locality: Heathman Plantation, Holly Ridge; decimalLatitude: 33.462079; decimalLongitude: -90.707222; geodeticDatum: WGS1984; **Identification:** identifiedBy: K. A. Parys; dateIdentified: 2017; **Event:** samplingProtocol: Bee Bowl (Yellow); eventDate: 2017-8-1; **Record Level:** type: PhysicalObject; language: en; rights: https://creativecommons.org/publicdomain/zero/1.0/; accessRights: http://vertnet.org/resources/norms.html; institutionCode: USDA-ARS; collectionCode: SIMRU; basisOfRecord: PreservedSpecimen
- **Type status:** Other material. **Occurrence:** catalogNumber: SIMRU21965; recordedBy: K. A. Parys; individualCount: 1; sex: female; lifeStage: adult; preparations: pin; occurrenceID: urn:USDA-ARS:SIMRU:SIMRU21965; **Taxon:** scientificName: Diadasia (Diadasia) enavata (Cresson, 1872); kingdom: Animalia; phylum: Arthropoda; class: Insecta; order: Hymenoptera; family: Apidae; genus: Diadasia; subgenus: Diadasia; specificEpithet: enavata; scientificNameAuthorship: (Cresson, 1872); **Location:** country: United States; stateProvince: Mississippi; county: Sunflower; locality: Heathman Plantation, Holly Ridge; decimalLatitude: 33.462079; decimalLongitude: -90.707222; geodeticDatum: WGS1984; **Identification:** identifiedBy: K. A. Parys; dateIdentified: 2017; **Event:** samplingProtocol: Bee Bowl (Yellow); eventDate: 2017-8-1; **Record Level:** type: PhysicalObject; language: en; rights: https://creativecommons.org/publicdomain/zero/1.0/; accessRights: http://vertnet.org/resources/norms.html; institutionCode: USDA-ARS; collectionCode: SIMRU; basisOfRecord: PreservedSpecimen
- **Type status:** Other material. **Occurrence:** catalogNumber: SIMRU21967; recordedBy: K. A. Parys; individualCount: 1; sex: female; lifeStage: adult; preparations: pin; occurrenceID: urn:USDA-ARS:SIMRU:SIMRU21967; **Taxon:** scientificName: Diadasia (Diadasia) enavata (Cresson, 1872); kingdom: Animalia; phylum: Arthropoda; class: Insecta; order: Hymenoptera; family: Apidae; genus: Diadasia; subgenus: Diadasia; specificEpithet: enavata; scientificNameAuthorship: (Cresson, 1872); **Location:** country: United States; stateProvince: Mississippi; county: Sunflower; locality: Heathman Plantation, Holly Ridge; decimalLatitude: 33.462079; decimalLongitude: -90.707222; geodeticDatum: WGS1984; **Identification:** identifiedBy: K. A. Parys; dateIdentified: 2017; **Event:** samplingProtocol: Bee Bowl (Yellow); eventDate: 2017-8-1; **Record Level:** type: PhysicalObject; language: en; rights: https://creativecommons.org/publicdomain/zero/1.0/; accessRights: http://vertnet.org/resources/norms.html; institutionCode: USDA-ARS; collectionCode: SIMRU; basisOfRecord: PreservedSpecimen
- **Type status:** Other material. **Occurrence:** catalogNumber: SIMRU21976; recordedBy: K. A. Parys; individualCount: 1; sex: male; lifeStage: adult; preparations: pin; occurrenceID: urn:USDA-ARS:SIMRU:SIMRU21976; **Taxon:** scientificName: Diadasia (Diadasia) enavata (Cresson, 1872); kingdom: Animalia; phylum: Arthropoda; class: Insecta; order: Hymenoptera; family: Apidae; genus: Diadasia; subgenus: Diadasia; specificEpithet: enavata; scientificNameAuthorship: (Cresson, 1872); **Location:** country: United States; stateProvince: Mississippi; county: Sunflower; locality: Heathman Plantation, Holly Ridge; decimalLatitude: 33.462079; decimalLongitude: -90.707222; geodeticDatum: WGS1984; **Identification:** identifiedBy: K. A. Parys; dateIdentified: 2017; **Event:** samplingProtocol: Bee Bowl (Yellow); eventDate: 2017-8-1; **Record Level:** type: PhysicalObject; language: en; rights: https://creativecommons.org/publicdomain/zero/1.0/; accessRights: http://vertnet.org/resources/norms.html; institutionCode: USDA-ARS; collectionCode: SIMRU; basisOfRecord: PreservedSpecimen
- **Type status:** Other material. **Occurrence:** catalogNumber: SIMRU21977; recordedBy: K. A. Parys; individualCount: 1; sex: female; lifeStage: adult; preparations: pin; occurrenceID: urn:USDA-ARS:SIMRU:SIMRU21977; **Taxon:** scientificName: Diadasia (Diadasia) enavata (Cresson, 1872); kingdom: Animalia; phylum: Arthropoda; class: Insecta; order: Hymenoptera; family: Apidae; genus: Diadasia; subgenus: Diadasia; specificEpithet: enavata; scientificNameAuthorship: (Cresson, 1872); **Location:** country: United States; stateProvince: Mississippi; county: Sunflower; locality: Heathman Plantation, Holly Ridge; decimalLatitude: 33.462079; decimalLongitude: -90.707222; geodeticDatum: WGS1984; **Identification:** identifiedBy: K. A. Parys; dateIdentified: 2017; **Event:** samplingProtocol: Bee Bowl (Yellow); eventDate: 2017-8-1; **Record Level:** type: PhysicalObject; language: en; rights: https://creativecommons.org/publicdomain/zero/1.0/; accessRights: http://vertnet.org/resources/norms.html; institutionCode: USDA-ARS; collectionCode: SIMRU; basisOfRecord: PreservedSpecimen
- **Type status:** Other material. **Occurrence:** catalogNumber: SIMRU22005; recordedBy: K. A. Parys; individualCount: 1; sex: female; lifeStage: adult; preparations: pin; occurrenceID: urn:USDA-ARS:SIMRU:SIMRU22005; **Taxon:** scientificName: Diadasia (Diadasia) enavata (Cresson, 1872); kingdom: Animalia; phylum: Arthropoda; class: Insecta; order: Hymenoptera; family: Apidae; genus: Diadasia; subgenus: Diadasia; specificEpithet: enavata; scientificNameAuthorship: (Cresson, 1872); **Location:** country: United States; stateProvince: Mississippi; county: Sunflower; locality: Heathman Plantation, Holly Ridge; decimalLatitude: 33.462079; decimalLongitude: -90.707222; geodeticDatum: WGS1984; **Identification:** identifiedBy: K. A. Parys; dateIdentified: 2017; **Event:** samplingProtocol: Bee Bowl (Yellow); eventDate: 2017-8-1; **Record Level:** type: PhysicalObject; language: en; rights: https://creativecommons.org/publicdomain/zero/1.0/; accessRights: http://vertnet.org/resources/norms.html; institutionCode: USDA-ARS; collectionCode: SIMRU; basisOfRecord: PreservedSpecimen
- **Type status:** Other material. **Occurrence:** catalogNumber: SIMRU22008; recordedBy: K. A. Parys; individualCount: 1; sex: female; lifeStage: adult; preparations: pin; occurrenceID: urn:USDA-ARS:SIMRU:SIMRU22008; **Taxon:** scientificName: Diadasia (Diadasia) enavata (Cresson, 1872); kingdom: Animalia; phylum: Arthropoda; class: Insecta; order: Hymenoptera; family: Apidae; genus: Diadasia; subgenus: Diadasia; specificEpithet: enavata; scientificNameAuthorship: (Cresson, 1872); **Location:** country: United States; stateProvince: Mississippi; county: Sunflower; locality: Heathman Plantation, Holly Ridge; decimalLatitude: 33.462079; decimalLongitude: -90.707222; geodeticDatum: WGS1984; **Identification:** identifiedBy: K. A. Parys; dateIdentified: 2017; **Event:** samplingProtocol: Bee Bowl (Yellow); eventDate: 2017-8-1; **Record Level:** type: PhysicalObject; language: en; rights: https://creativecommons.org/publicdomain/zero/1.0/; accessRights: http://vertnet.org/resources/norms.html; institutionCode: USDA-ARS; collectionCode: SIMRU; basisOfRecord: PreservedSpecimen
- **Type status:** Other material. **Occurrence:** catalogNumber: SIMRU22011; recordedBy: K. A. Parys; individualCount: 1; sex: female; lifeStage: adult; preparations: pin; occurrenceID: urn:USDA-ARS:SIMRU:SIMRU22011; **Taxon:** scientificName: Diadasia (Diadasia) enavata (Cresson, 1872); kingdom: Animalia; phylum: Arthropoda; class: Insecta; order: Hymenoptera; family: Apidae; genus: Diadasia; subgenus: Diadasia; specificEpithet: enavata; scientificNameAuthorship: (Cresson, 1872); **Location:** country: United States; stateProvince: Mississippi; county: Sunflower; locality: Heathman Plantation, Holly Ridge; decimalLatitude: 33.462079; decimalLongitude: -90.707222; geodeticDatum: WGS1984; **Identification:** identifiedBy: K. A. Parys; dateIdentified: 2017; **Event:** samplingProtocol: Bee Bowl (Yellow); eventDate: 2017-8-1; **Record Level:** type: PhysicalObject; language: en; rights: https://creativecommons.org/publicdomain/zero/1.0/; accessRights: http://vertnet.org/resources/norms.html; institutionCode: USDA-ARS; collectionCode: SIMRU; basisOfRecord: PreservedSpecimen
- **Type status:** Other material. **Occurrence:** catalogNumber: SIMRU22016; recordedBy: K. A. Parys; individualCount: 1; sex: female; lifeStage: adult; preparations: pin; occurrenceID: urn:USDA-ARS:SIMRU:SIMRU22016; **Taxon:** scientificName: Diadasia (Diadasia) enavata (Cresson, 1872); kingdom: Animalia; phylum: Arthropoda; class: Insecta; order: Hymenoptera; family: Apidae; genus: Diadasia; subgenus: Diadasia; specificEpithet: enavata; scientificNameAuthorship: (Cresson, 1872); **Location:** country: United States; stateProvince: Mississippi; county: Sunflower; locality: Heathman Plantation, Holly Ridge; decimalLatitude: 33.462079; decimalLongitude: -90.707222; geodeticDatum: WGS1984; **Identification:** identifiedBy: K. A. Parys; dateIdentified: 2017; **Event:** samplingProtocol: Bee Bowl (Yellow); eventDate: 2017-8-1; **Record Level:** type: PhysicalObject; language: en; rights: https://creativecommons.org/publicdomain/zero/1.0/; accessRights: http://vertnet.org/resources/norms.html; institutionCode: USDA-ARS; collectionCode: SIMRU; basisOfRecord: PreservedSpecimen
- **Type status:** Other material. **Occurrence:** catalogNumber: SIMRU22017; recordedBy: K. A. Parys; individualCount: 1; sex: female; lifeStage: adult; preparations: pin; occurrenceID: urn:USDA-ARS:SIMRU:SIMRU22017; **Taxon:** scientificName: Diadasia (Diadasia) enavata (Cresson, 1872); kingdom: Animalia; phylum: Arthropoda; class: Insecta; order: Hymenoptera; family: Apidae; genus: Diadasia; subgenus: Diadasia; specificEpithet: enavata; scientificNameAuthorship: (Cresson, 1872); **Location:** country: United States; stateProvince: Mississippi; county: Sunflower; locality: Heathman Plantation, Holly Ridge; decimalLatitude: 33.462079; decimalLongitude: -90.707222; geodeticDatum: WGS1984; **Identification:** identifiedBy: K. A. Parys; dateIdentified: 2017; **Event:** samplingProtocol: Bee Bowl (Yellow); eventDate: 2017-8-1; **Record Level:** type: PhysicalObject; language: en; rights: https://creativecommons.org/publicdomain/zero/1.0/; accessRights: http://vertnet.org/resources/norms.html; institutionCode: USDA-ARS; collectionCode: SIMRU; basisOfRecord: PreservedSpecimen
- **Type status:** Other material. **Occurrence:** catalogNumber: SIMRU22018; recordedBy: K. A. Parys; individualCount: 1; sex: female; lifeStage: adult; preparations: pin; occurrenceID: urn:USDA-ARS:SIMRU:SIMRU22018; **Taxon:** scientificName: Diadasia (Diadasia) enavata (Cresson, 1872); kingdom: Animalia; phylum: Arthropoda; class: Insecta; order: Hymenoptera; family: Apidae; genus: Diadasia; subgenus: Diadasia; specificEpithet: enavata; scientificNameAuthorship: (Cresson, 1872); **Location:** country: United States; stateProvince: Mississippi; county: Sunflower; locality: Heathman Plantation, Holly Ridge; decimalLatitude: 33.462079; decimalLongitude: -90.707222; geodeticDatum: WGS1984; **Identification:** identifiedBy: K. A. Parys; dateIdentified: 2017; **Event:** samplingProtocol: Bee Bowl (Yellow); eventDate: 2017-8-1; **Record Level:** type: PhysicalObject; language: en; rights: https://creativecommons.org/publicdomain/zero/1.0/; accessRights: http://vertnet.org/resources/norms.html; institutionCode: USDA-ARS; collectionCode: SIMRU; basisOfRecord: PreservedSpecimen
- **Type status:** Other material. **Occurrence:** catalogNumber: SIMRU22019; recordedBy: K. A. Parys; individualCount: 1; sex: female; lifeStage: adult; preparations: pin; occurrenceID: urn:USDA-ARS:SIMRU:SIMRU22019; **Taxon:** scientificName: Diadasia (Diadasia) enavata (Cresson, 1872); kingdom: Animalia; phylum: Arthropoda; class: Insecta; order: Hymenoptera; family: Apidae; genus: Diadasia; subgenus: Diadasia; specificEpithet: enavata; scientificNameAuthorship: (Cresson, 1872); **Location:** country: United States; stateProvince: Mississippi; county: Sunflower; locality: Heathman Plantation, Holly Ridge; decimalLatitude: 33.462079; decimalLongitude: -90.707222; geodeticDatum: WGS1984; **Identification:** identifiedBy: K. A. Parys; dateIdentified: 2017; **Event:** samplingProtocol: Bee Bowl (Yellow); eventDate: 2017-8-1; **Record Level:** type: PhysicalObject; language: en; rights: https://creativecommons.org/publicdomain/zero/1.0/; accessRights: http://vertnet.org/resources/norms.html; institutionCode: USDA-ARS; collectionCode: SIMRU; basisOfRecord: PreservedSpecimen
- **Type status:** Other material. **Occurrence:** catalogNumber: SIMRU22020; recordedBy: K. A. Parys; individualCount: 1; sex: female; lifeStage: adult; preparations: pin; occurrenceID: urn:USDA-ARS:SIMRU:SIMRU22020; **Taxon:** scientificName: Diadasia (Diadasia) enavata (Cresson, 1872); kingdom: Animalia; phylum: Arthropoda; class: Insecta; order: Hymenoptera; family: Apidae; genus: Diadasia; subgenus: Diadasia; specificEpithet: enavata; scientificNameAuthorship: (Cresson, 1872); **Location:** country: United States; stateProvince: Mississippi; county: Sunflower; locality: Heathman Plantation, Holly Ridge; decimalLatitude: 33.462079; decimalLongitude: -90.707222; geodeticDatum: WGS1984; **Identification:** identifiedBy: K. A. Parys; dateIdentified: 2017; **Event:** samplingProtocol: Bee Bowl (Yellow); eventDate: 2017-8-1; **Record Level:** type: PhysicalObject; language: en; rights: https://creativecommons.org/publicdomain/zero/1.0/; accessRights: http://vertnet.org/resources/norms.html; institutionCode: USDA-ARS; collectionCode: SIMRU; basisOfRecord: PreservedSpecimen
- **Type status:** Other material. **Occurrence:** catalogNumber: SIMRU22024; recordedBy: K. A. Parys; individualCount: 1; sex: female; lifeStage: adult; preparations: pin; occurrenceID: urn:USDA-ARS:SIMRU:SIMRU22024; **Taxon:** scientificName: Diadasia (Diadasia) enavata (Cresson, 1872); kingdom: Animalia; phylum: Arthropoda; class: Insecta; order: Hymenoptera; family: Apidae; genus: Diadasia; subgenus: Diadasia; specificEpithet: enavata; scientificNameAuthorship: (Cresson, 1872); **Location:** country: United States; stateProvince: Mississippi; county: Sunflower; locality: Heathman Plantation, Holly Ridge; decimalLatitude: 33.462079; decimalLongitude: -90.707222; geodeticDatum: WGS1984; **Identification:** identifiedBy: K. A. Parys; dateIdentified: 2017; **Event:** samplingProtocol: Bee Bowl (Yellow); eventDate: 2017-8-1; **Record Level:** type: PhysicalObject; language: en; rights: https://creativecommons.org/publicdomain/zero/1.0/; accessRights: http://vertnet.org/resources/norms.html; institutionCode: USDA-ARS; collectionCode: SIMRU; basisOfRecord: PreservedSpecimen
- **Type status:** Other material. **Occurrence:** catalogNumber: SIMRU22037; recordedBy: K. A. Parys; individualCount: 1; sex: female; lifeStage: adult; preparations: pin; occurrenceID: urn:USDA-ARS:SIMRU:SIMRU22037; **Taxon:** scientificName: Diadasia (Diadasia) enavata (Cresson, 1872); kingdom: Animalia; phylum: Arthropoda; class: Insecta; order: Hymenoptera; family: Apidae; genus: Diadasia; subgenus: Diadasia; specificEpithet: enavata; scientificNameAuthorship: (Cresson, 1872); **Location:** country: United States; stateProvince: Mississippi; county: Sunflower; locality: Heathman Plantation, Holly Ridge; decimalLatitude: 33.462079; decimalLongitude: -90.707222; geodeticDatum: WGS1984; **Identification:** identifiedBy: K. A. Parys; dateIdentified: 2017; **Event:** samplingProtocol: Bee Bowl (Yellow); eventDate: 2017-8-1; **Record Level:** type: PhysicalObject; language: en; rights: https://creativecommons.org/publicdomain/zero/1.0/; accessRights: http://vertnet.org/resources/norms.html; institutionCode: USDA-ARS; collectionCode: SIMRU; basisOfRecord: PreservedSpecimen
- **Type status:** Other material. **Occurrence:** catalogNumber: SIMRU22039; recordedBy: K. A. Parys; individualCount: 1; sex: female; lifeStage: adult; preparations: pin; occurrenceID: urn:USDA-ARS:SIMRU:SIMRU22039; **Taxon:** scientificName: Diadasia (Diadasia) enavata (Cresson, 1872); kingdom: Animalia; phylum: Arthropoda; class: Insecta; order: Hymenoptera; family: Apidae; genus: Diadasia; subgenus: Diadasia; specificEpithet: enavata; scientificNameAuthorship: (Cresson, 1872); **Location:** country: United States; stateProvince: Mississippi; county: Sunflower; locality: Heathman Plantation, Holly Ridge; decimalLatitude: 33.462079; decimalLongitude: -90.707222; geodeticDatum: WGS1984; **Identification:** identifiedBy: K. A. Parys; dateIdentified: 2017; **Event:** samplingProtocol: Bee Bowl (Yellow); eventDate: 2017-8-1; **Record Level:** type: PhysicalObject; language: en; rights: https://creativecommons.org/publicdomain/zero/1.0/; accessRights: http://vertnet.org/resources/norms.html; institutionCode: USDA-ARS; collectionCode: SIMRU; basisOfRecord: PreservedSpecimen
- **Type status:** Other material. **Occurrence:** catalogNumber: SIMRU22046; recordedBy: K. A. Parys; individualCount: 1; sex: female; lifeStage: adult; preparations: pin; occurrenceID: urn:USDA-ARS:SIMRU:SIMRU22046; **Taxon:** scientificName: Diadasia (Diadasia) enavata (Cresson, 1872); kingdom: Animalia; phylum: Arthropoda; class: Insecta; order: Hymenoptera; family: Apidae; genus: Diadasia; subgenus: Diadasia; specificEpithet: enavata; scientificNameAuthorship: (Cresson, 1872); **Location:** country: United States; stateProvince: Mississippi; county: Sunflower; locality: Heathman Plantation, Holly Ridge; decimalLatitude: 33.462079; decimalLongitude: -90.707222; geodeticDatum: WGS1984; **Identification:** identifiedBy: K. A. Parys; dateIdentified: 2017; **Event:** samplingProtocol: Bee Bowl (Blue); eventDate: 2017-8-1; **Record Level:** type: PhysicalObject; language: en; rights: https://creativecommons.org/publicdomain/zero/1.0/; accessRights: http://vertnet.org/resources/norms.html; institutionCode: USDA-ARS; collectionCode: SIMRU; basisOfRecord: PreservedSpecimen
- **Type status:** Other material. **Occurrence:** catalogNumber: SIMRU22049; recordedBy: K. A. Parys; individualCount: 1; sex: female; lifeStage: adult; preparations: pin; occurrenceID: urn:USDA-ARS:SIMRU:SIMRU22049; **Taxon:** scientificName: Diadasia (Diadasia) enavata (Cresson, 1872); kingdom: Animalia; phylum: Arthropoda; class: Insecta; order: Hymenoptera; family: Apidae; genus: Diadasia; subgenus: Diadasia; specificEpithet: enavata; scientificNameAuthorship: (Cresson, 1872); **Location:** country: United States; stateProvince: Mississippi; county: Sunflower; locality: Heathman Plantation, Holly Ridge; decimalLatitude: 33.462079; decimalLongitude: -90.707222; geodeticDatum: WGS1984; **Identification:** identifiedBy: K. A. Parys; dateIdentified: 2017; **Event:** samplingProtocol: Bee Bowl (Blue); eventDate: 2017-8-1; **Record Level:** type: PhysicalObject; language: en; rights: https://creativecommons.org/publicdomain/zero/1.0/; accessRights: http://vertnet.org/resources/norms.html; institutionCode: USDA-ARS; collectionCode: SIMRU; basisOfRecord: PreservedSpecimen
- **Type status:** Other material. **Occurrence:** catalogNumber: SIMRU22057; recordedBy: K. A. Parys; individualCount: 1; sex: female; lifeStage: adult; preparations: pin; occurrenceID: urn:USDA-ARS:SIMRU:SIMRU22057; **Taxon:** scientificName: Diadasia (Diadasia) enavata (Cresson, 1872); kingdom: Animalia; phylum: Arthropoda; class: Insecta; order: Hymenoptera; family: Apidae; genus: Diadasia; subgenus: Diadasia; specificEpithet: enavata; scientificNameAuthorship: (Cresson, 1872); **Location:** country: United States; stateProvince: Mississippi; county: Sunflower; locality: Heathman Plantation, Holly Ridge; decimalLatitude: 33.462079; decimalLongitude: -90.707222; geodeticDatum: WGS1984; **Identification:** identifiedBy: K. A. Parys; dateIdentified: 2017; **Event:** samplingProtocol: Bee Bowl (Blue); eventDate: 2017-8-1; **Record Level:** type: PhysicalObject; language: en; rights: https://creativecommons.org/publicdomain/zero/1.0/; accessRights: http://vertnet.org/resources/norms.html; institutionCode: USDA-ARS; collectionCode: SIMRU; basisOfRecord: PreservedSpecimen
- **Type status:** Other material. **Occurrence:** catalogNumber: SIMRU22070; recordedBy: K. A. Parys; individualCount: 1; sex: female; lifeStage: adult; preparations: pin; occurrenceID: urn:USDA-ARS:SIMRU:SIMRU22070; **Taxon:** scientificName: Diadasia (Diadasia) enavata (Cresson, 1872); kingdom: Animalia; phylum: Arthropoda; class: Insecta; order: Hymenoptera; family: Apidae; genus: Diadasia; subgenus: Diadasia; specificEpithet: enavata; scientificNameAuthorship: (Cresson, 1872); **Location:** country: United States; stateProvince: Mississippi; county: Sunflower; locality: Heathman Plantation, Holly Ridge; decimalLatitude: 33.462079; decimalLongitude: -90.707222; geodeticDatum: WGS1984; **Identification:** identifiedBy: K. A. Parys; dateIdentified: 2017; **Event:** samplingProtocol: Bee Bowl (Blue); eventDate: 2017-8-1; **Record Level:** type: PhysicalObject; language: en; rights: https://creativecommons.org/publicdomain/zero/1.0/; accessRights: http://vertnet.org/resources/norms.html; institutionCode: USDA-ARS; collectionCode: SIMRU; basisOfRecord: PreservedSpecimen
- **Type status:** Other material. **Occurrence:** catalogNumber: SIMRU22073; recordedBy: K. A. Parys; individualCount: 1; sex: female; lifeStage: adult; preparations: pin; occurrenceID: urn:USDA-ARS:SIMRU:SIMRU22073; **Taxon:** scientificName: Diadasia (Diadasia) enavata (Cresson, 1872); kingdom: Animalia; phylum: Arthropoda; class: Insecta; order: Hymenoptera; family: Apidae; genus: Diadasia; subgenus: Diadasia; specificEpithet: enavata; scientificNameAuthorship: (Cresson, 1872); **Location:** country: United States; stateProvince: Mississippi; county: Sunflower; locality: Heathman Plantation, Holly Ridge; decimalLatitude: 33.462079; decimalLongitude: -90.707222; geodeticDatum: WGS1984; **Identification:** identifiedBy: K. A. Parys; dateIdentified: 2017; **Event:** samplingProtocol: Bee Bowl (Blue); eventDate: 2017-8-1; **Record Level:** type: PhysicalObject; language: en; rights: https://creativecommons.org/publicdomain/zero/1.0/; accessRights: http://vertnet.org/resources/norms.html; institutionCode: USDA-ARS; collectionCode: SIMRU; basisOfRecord: PreservedSpecimen
- **Type status:** Other material. **Occurrence:** catalogNumber: SIMRU22088; recordedBy: K. A. Parys; individualCount: 1; sex: female; lifeStage: adult; preparations: pin; occurrenceID: urn:USDA-ARS:SIMRU:SIMRU22088; **Taxon:** scientificName: Diadasia (Diadasia) enavata (Cresson, 1872); kingdom: Animalia; phylum: Arthropoda; class: Insecta; order: Hymenoptera; family: Apidae; genus: Diadasia; subgenus: Diadasia; specificEpithet: enavata; scientificNameAuthorship: (Cresson, 1872); **Location:** country: United States; stateProvince: Mississippi; county: Sunflower; locality: Heathman Plantation, Holly Ridge; decimalLatitude: 33.462079; decimalLongitude: -90.707222; geodeticDatum: WGS1984; **Identification:** identifiedBy: K. A. Parys; dateIdentified: 2017; **Event:** samplingProtocol: Bee Bowl (Blue); eventDate: 2017-8-1; **Record Level:** type: PhysicalObject; language: en; rights: https://creativecommons.org/publicdomain/zero/1.0/; accessRights: http://vertnet.org/resources/norms.html; institutionCode: USDA-ARS; collectionCode: SIMRU; basisOfRecord: PreservedSpecimen
- **Type status:** Other material. **Occurrence:** catalogNumber: SIMRU22102; recordedBy: K. A. Parys; individualCount: 1; sex: female; lifeStage: adult; preparations: pin; occurrenceID: urn:USDA-ARS:SIMRU:SIMRU22102; **Taxon:** scientificName: Diadasia (Diadasia) enavata (Cresson, 1872); kingdom: Animalia; phylum: Arthropoda; class: Insecta; order: Hymenoptera; family: Apidae; genus: Diadasia; subgenus: Diadasia; specificEpithet: enavata; scientificNameAuthorship: (Cresson, 1872); **Location:** country: United States; stateProvince: Mississippi; county: Sharkey; locality: Rolling Fork; decimalLatitude: 32.91696; decimalLongitude: -90.92081; geodeticDatum: WGS1984; **Identification:** identifiedBy: K. A. Parys; dateIdentified: 2017; **Event:** samplingProtocol: Bee Bowl (Blue); eventDate: 2017-8-1; **Record Level:** type: PhysicalObject; language: en; rights: https://creativecommons.org/publicdomain/zero/1.0/; accessRights: http://vertnet.org/resources/norms.html; institutionCode: USDA-ARS; collectionCode: SIMRU; basisOfRecord: PreservedSpecimen
- **Type status:** Other material. **Occurrence:** catalogNumber: SIMRU22114; recordedBy: K. A. Parys; individualCount: 1; sex: female; lifeStage: adult; preparations: pin; occurrenceID: urn:USDA-ARS:SIMRU:SIMRU22114; **Taxon:** scientificName: Diadasia (Diadasia) enavata (Cresson, 1872); kingdom: Animalia; phylum: Arthropoda; class: Insecta; order: Hymenoptera; family: Apidae; genus: Diadasia; subgenus: Diadasia; specificEpithet: enavata; scientificNameAuthorship: (Cresson, 1872); **Location:** country: United States; stateProvince: Mississippi; county: Sunflower; locality: Heathman Plantation, Holly Ridge; decimalLatitude: 33.462079; decimalLongitude: -90.707222; geodeticDatum: WGS1984; **Identification:** identifiedBy: K. A. Parys; dateIdentified: 2017; **Event:** samplingProtocol: Bee Bowl (Blue); eventDate: 2017-8-1; **Record Level:** type: PhysicalObject; language: en; rights: https://creativecommons.org/publicdomain/zero/1.0/; accessRights: http://vertnet.org/resources/norms.html; institutionCode: USDA-ARS; collectionCode: SIMRU; basisOfRecord: PreservedSpecimen
- **Type status:** Other material. **Occurrence:** catalogNumber: SIMRU22119; recordedBy: K. A. Parys; individualCount: 1; sex: female; lifeStage: adult; preparations: pin; occurrenceID: urn:USDA-ARS:SIMRU:SIMRU22119; **Taxon:** scientificName: Diadasia (Diadasia) enavata (Cresson, 1872); kingdom: Animalia; phylum: Arthropoda; class: Insecta; order: Hymenoptera; family: Apidae; genus: Diadasia; subgenus: Diadasia; specificEpithet: enavata; scientificNameAuthorship: (Cresson, 1872); **Location:** country: United States; stateProvince: Mississippi; county: Sunflower; locality: Heathman Plantation, Holly Ridge; decimalLatitude: 33.462079; decimalLongitude: -90.707222; geodeticDatum: WGS1984; **Identification:** identifiedBy: K. A. Parys; dateIdentified: 2017; **Event:** samplingProtocol: Bee Bowl (Blue); eventDate: 2017-8-1; **Record Level:** type: PhysicalObject; language: en; rights: https://creativecommons.org/publicdomain/zero/1.0/; accessRights: http://vertnet.org/resources/norms.html; institutionCode: USDA-ARS; collectionCode: SIMRU; basisOfRecord: PreservedSpecimen
- **Type status:** Other material. **Occurrence:** catalogNumber: SIMRU22163; recordedBy: K. A. Parys; individualCount: 1; sex: female; lifeStage: adult; preparations: pin; occurrenceID: urn:USDA-ARS:SIMRU:SIMRU22163; **Taxon:** scientificName: Diadasia (Diadasia) enavata (Cresson, 1872); kingdom: Animalia; phylum: Arthropoda; class: Insecta; order: Hymenoptera; family: Apidae; genus: Diadasia; subgenus: Diadasia; specificEpithet: enavata; scientificNameAuthorship: (Cresson, 1872); **Location:** country: United States; stateProvince: Mississippi; county: Sunflower; locality: Heathman Plantation, Holly Ridge; decimalLatitude: 33.462079; decimalLongitude: -90.707222; geodeticDatum: WGS1984; **Identification:** identifiedBy: K. A. Parys; dateIdentified: 2017; **Event:** samplingProtocol: Bee Bowl (Blue); eventDate: 2017-8-1; **Record Level:** type: PhysicalObject; language: en; rights: https://creativecommons.org/publicdomain/zero/1.0/; accessRights: http://vertnet.org/resources/norms.html; institutionCode: USDA-ARS; collectionCode: SIMRU; basisOfRecord: PreservedSpecimen
- **Type status:** Other material. **Occurrence:** catalogNumber: SIMRU22168; recordedBy: K. A. Parys; individualCount: 1; sex: female; lifeStage: adult; preparations: pin; occurrenceID: urn:USDA-ARS:SIMRU:SIMRU22168; **Taxon:** scientificName: Diadasia (Diadasia) enavata (Cresson, 1872); kingdom: Animalia; phylum: Arthropoda; class: Insecta; order: Hymenoptera; family: Apidae; genus: Diadasia; subgenus: Diadasia; specificEpithet: enavata; scientificNameAuthorship: (Cresson, 1872); **Location:** country: United States; stateProvince: Mississippi; county: Sunflower; locality: Heathman Plantation, Holly Ridge; decimalLatitude: 33.462079; decimalLongitude: -90.707222; geodeticDatum: WGS1984; **Identification:** identifiedBy: K. A. Parys; dateIdentified: 2017; **Event:** samplingProtocol: Bee Bowl (Blue); eventDate: 2017-8-1; **Record Level:** type: PhysicalObject; language: en; rights: https://creativecommons.org/publicdomain/zero/1.0/; accessRights: http://vertnet.org/resources/norms.html; institutionCode: USDA-ARS; collectionCode: SIMRU; basisOfRecord: PreservedSpecimen

##### Notes

The genus *Diadasia* only occurs in the New World and has been traditionally collected in western and south-western parts of the United States, though it also occurs in western Canada (Hurd et al. 1980, Sheffield et al. 2017). This species has the largest distribution of any species of *Diadasia*, ranging from Washington and California on the west coast, east through Texas and Missouri (Hurd et al. 1980). Descriptions and illustrations of the ground nests have been published (Bohart 1952, Linsley and MacSwain 1957). The documented range of *D. enavata* was recently extended into Arkansas (Stephenson et al. 2018) and now into Mississippi.

**Figure 7.**
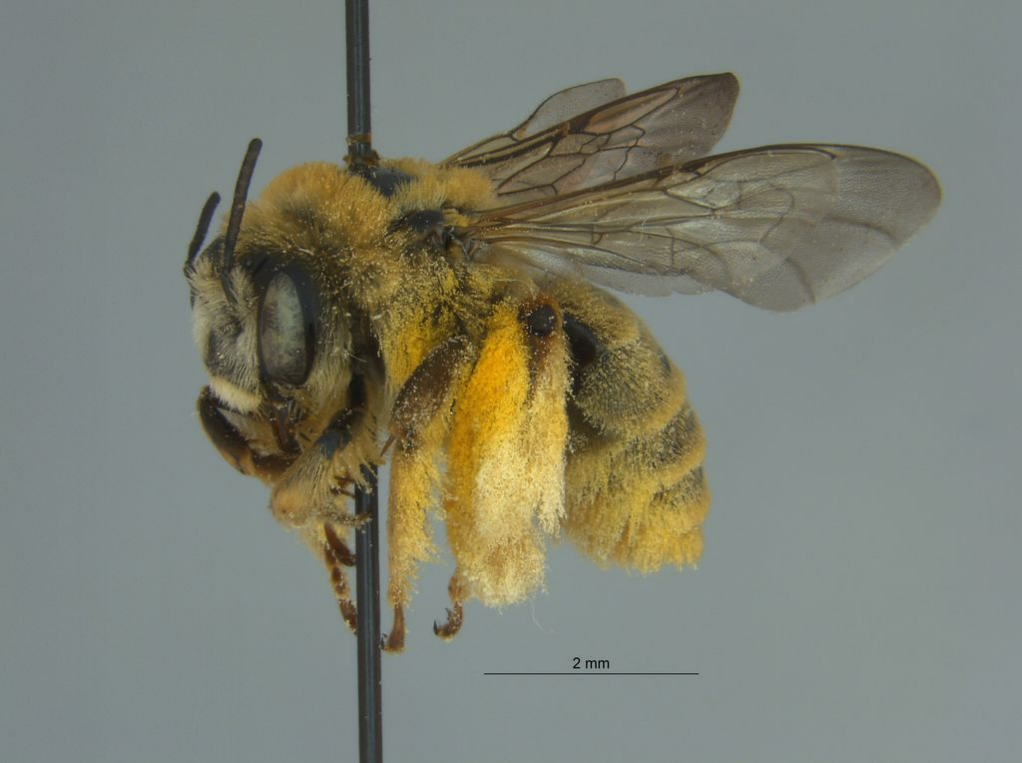
Female *Diadasia enavata*, lateral view.

Over 100 specimens of *D. enavata* have been collected from Bolivar, Sunflower, Sharkey and Washington Counties in the Delta region of Mississippi (Fig. 7). The majority of the specimens were picked up in bee bowls in or around sunflower fields, though specimens were also picked up in corn, soybeans and sorghum fields.

#### Eucerini

#### Peponapis (Xenopeponapis) crassidentata

(Cockerell, 1949)

##### Materials

- **Type status:** Other material. **Occurrence:** catalogNumber: SIMRU10399; recordedBy: K. A. Parys; individualCount: 1; sex: male; lifeStage: adult; preparations: pin; occurrenceID: urn:USDA-ARS:SIMRU:SIMRU10399; **Taxon:** scientificName: Peponapis (Xenopeponapis) crassidentata (Cockerell, 1949); kingdom: Animalia; phylum: Arthropoda; class: Insecta; order: Hymenoptera; family: Apidae; genus: Peponapis; subgenus: Xenopeponapis; specificEpithet: crassidentata; scientificNameAuthorship: (Cockerell, 1949); **Location:** country: United States; stateProvince: Mississippi; county: Sharkey; locality: Cary; decimalLatitude: 32.785538; decimalLongitude: -90.964959; geodeticDatum: WGS1984; **Identification:** identifiedBy: T. Griswold; dateIdentified: 2017; **Event:** samplingProtocol: Bee Bowl (Blue); eventDate: 2016-8-10; **Record Level:** type: PhysicalObject; language: en; rights: https://creativecommons.org/publicdomain/zero/1.0/; accessRights: http://vertnet.org/resources/norms.html; institutionCode: USDA-ARS; collectionCode: SIMRU; basisOfRecord: PreservedSpecimen

##### Notes

This species is only known from the state of Texas within the United States, though it ranges south through México and in Central America to Costa Rica (Hurd and Linsley 1964, Ayala and Griswold 2012). Unlike the closely related *Peponapis pruinosa* (Say), which is a ground nester, *P. crassidentata* nests in bare vertical banks of sandy soil (Delgado-Carrillo et al. 2017). One single male specimen was collected in a blue vane trap located on the edge of a soybean field in Sharkey County Mississippi (Fig. 8).

## Discussion

Prior to our sampling, there were no known lists of species that occur in the Mississippi Delta and the reported number of species in the state of Mississippi as a whole was 184. With the additional seven species included here, the number of species reported in published literature for the state is increased to 191.

All of the species recorded here from Mississippi represent large increases in their known range, with the exception of *A. passiflorae*, as Mississippi is between other states with known populations of this species. *Anthemurgus* is considered "rare" in Michener et al. (1994) and was not included at all in Mitchell (1960), but was found to be locally abundant on *P. lutea* (a weedy vine) at our collection location. While neither was particularly abundant, *Andrena obscuripennis* and *Dieunomia bolliana* were both also collected from weedy roadside plants while sweep-netting for populations of agricultural pest insects.

*Diadasia enavata* was also very abundant locally and widespread across our collection locations within sunflower fields. These fields are primarily planted for fall (autumn) dove hunting in the region and are largely unmanaged as they are not harvested. The specimens included here are from several counties in the Mississippi Delta and were all collected from agricultural farms. *Triepeolus subnitens* was also collected from these planted sunflower fields. The single specimen of *P. crassidentata* was also collected from an agricultural field, but as a singleton, it may be a tourist or suggest a low population density. While this specimen was collected from a trap in a soybean field, members of *Peponapis* are known to be *Cucurbita* specialists and, ergo, unlikely to be foraging in the soybean field.

These seven species were unexpected in collections made in the Mississippi Delta, especially given their prior known ranges. Their presence, especially those that are abundant, indicates that established populations occur in the region. Further collections within the Mississippi Alluvial Valley and, in particular, the Mississippi Delta, are expected to yield additional records for the state and clarify distributional records of other species collected within the state of Mississippi. This study may also have implications for undersampled areas in other places, with distributions of species unexpectedly extending into these areas.

## Supplementary Material

XML Treatment for Andrenidae

XML Treatment for Andrena (Melandrena) obscuripennis

XML Treatment for Anthemurgus passiflorae

XML Treatment for Halictidae

XML Treatment for Dieunomia (Dieunomia) bolliana

XML Treatment for Apidae

XML Treatment for Brachynomadini

XML Treatment for Brachynomada (Melanomada) nimia

XML Treatment for Epeolini

XML Treatment for Triepeolus subnitens

XML Treatment for Apinae

XML Treatment for Emphorini

XML Treatment for Diadasia (Diadasia) enavata

XML Treatment for Eucerini

XML Treatment for Peponapis (Xenopeponapis) crassidentata

## Figures and Tables

**Figure 1a.**
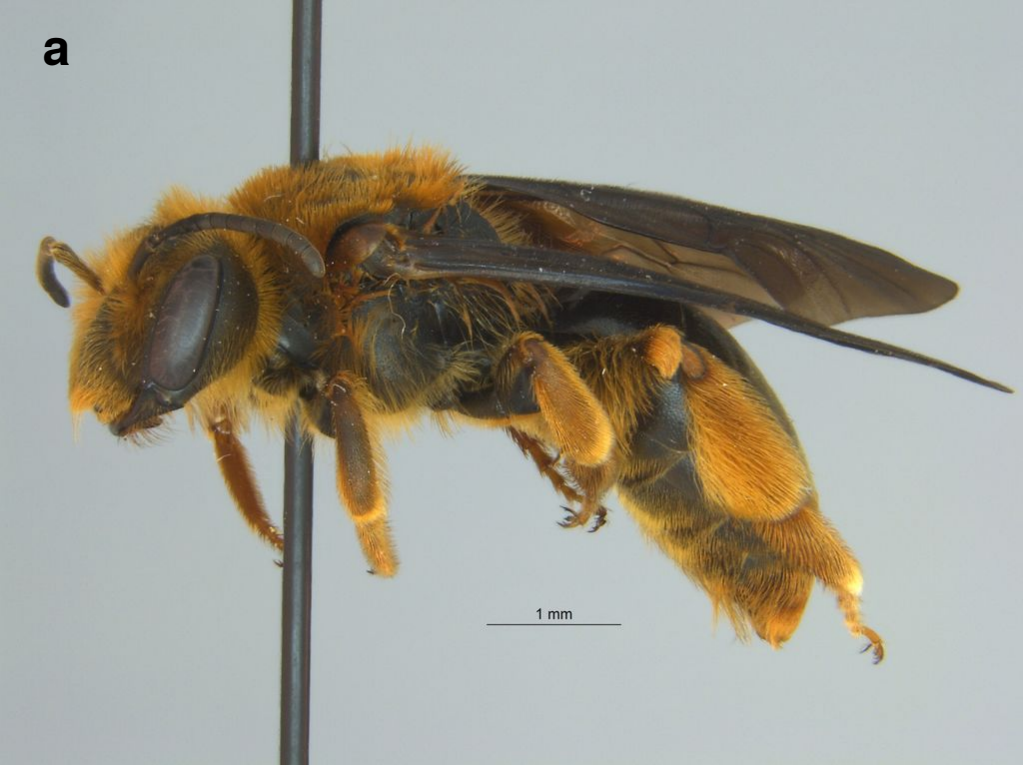
Lateral view.

**Figure 1b.**
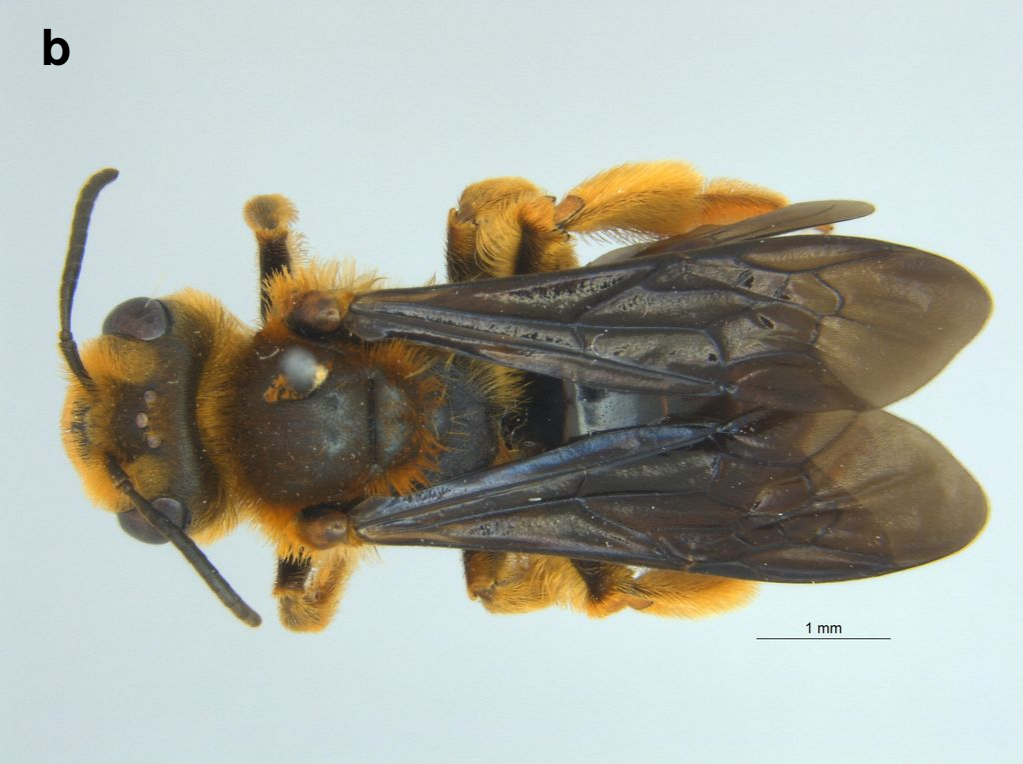
Dorsal view.

**Figure 3a.**
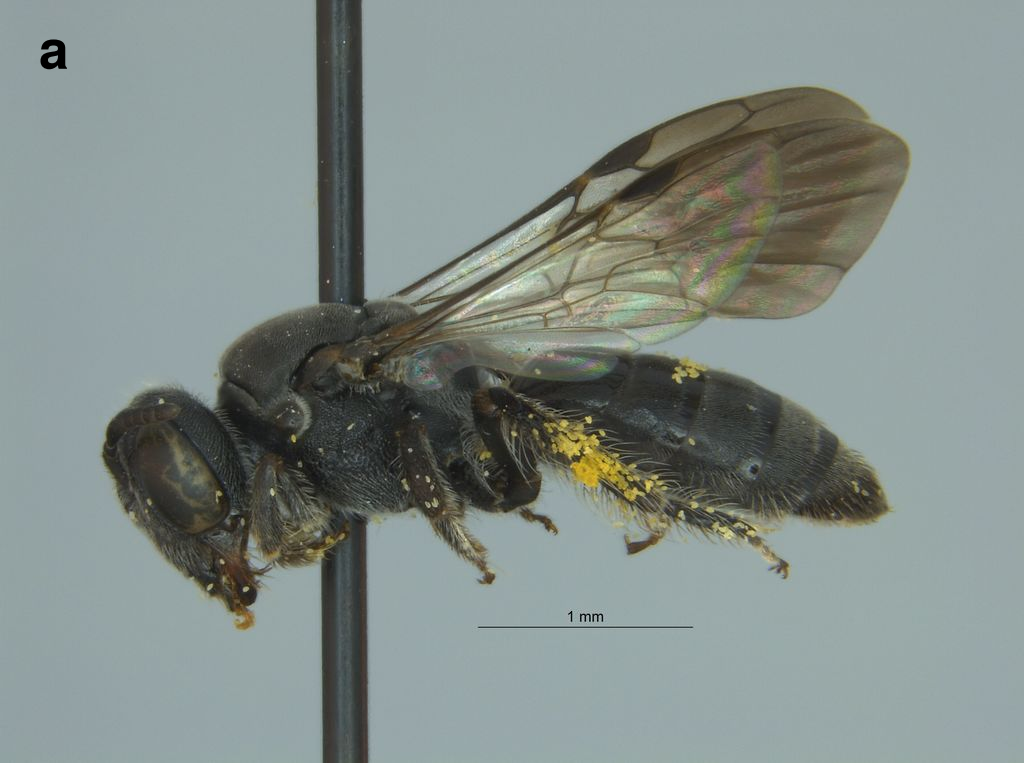
Female, lateral view.

**Figure 3b.**
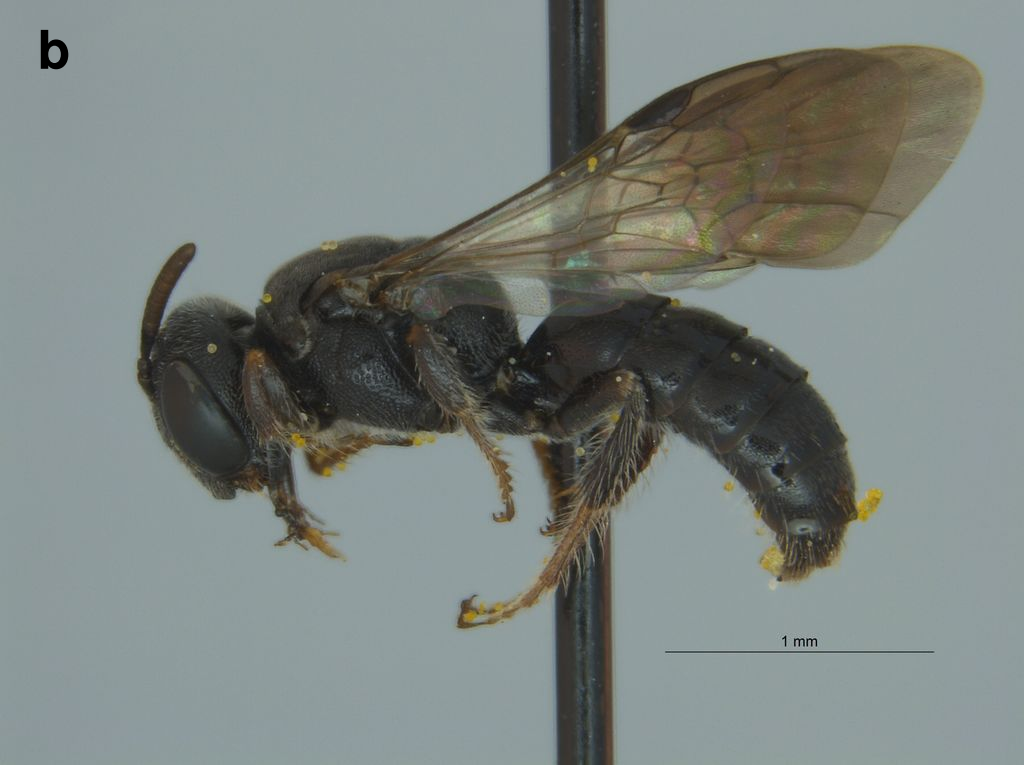
Male, lateral view.

**Figure 3c.**
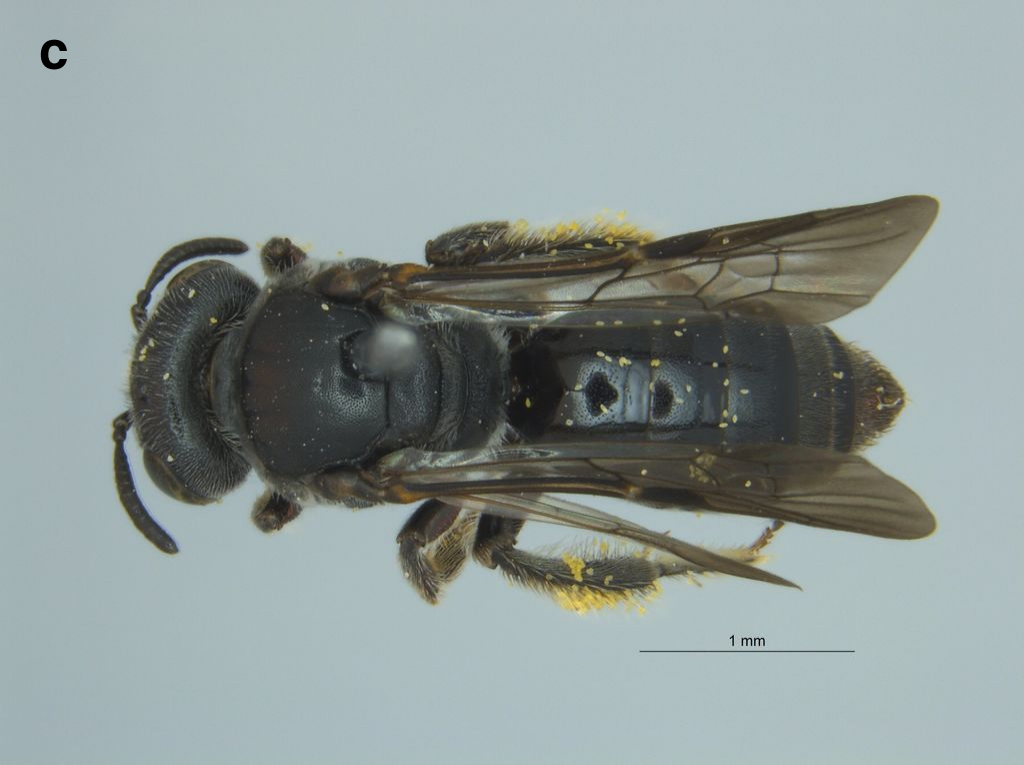
Female, dorsal view.

**Figure 3d.**
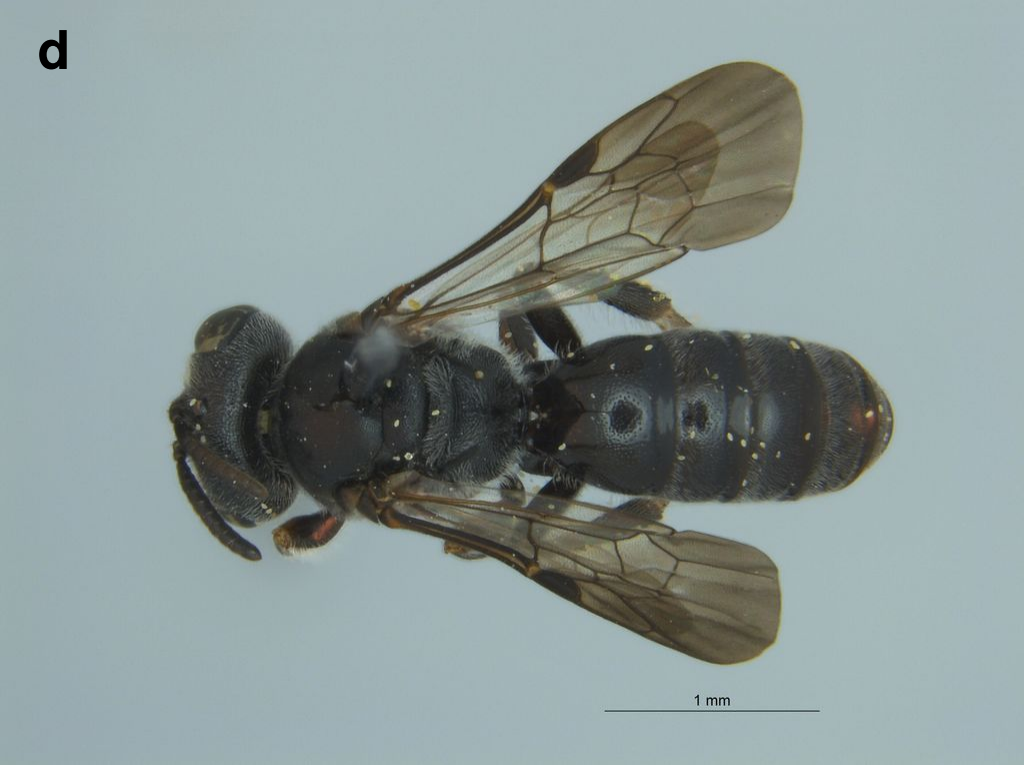
Male, dorsal view.

**Figure 4a.**
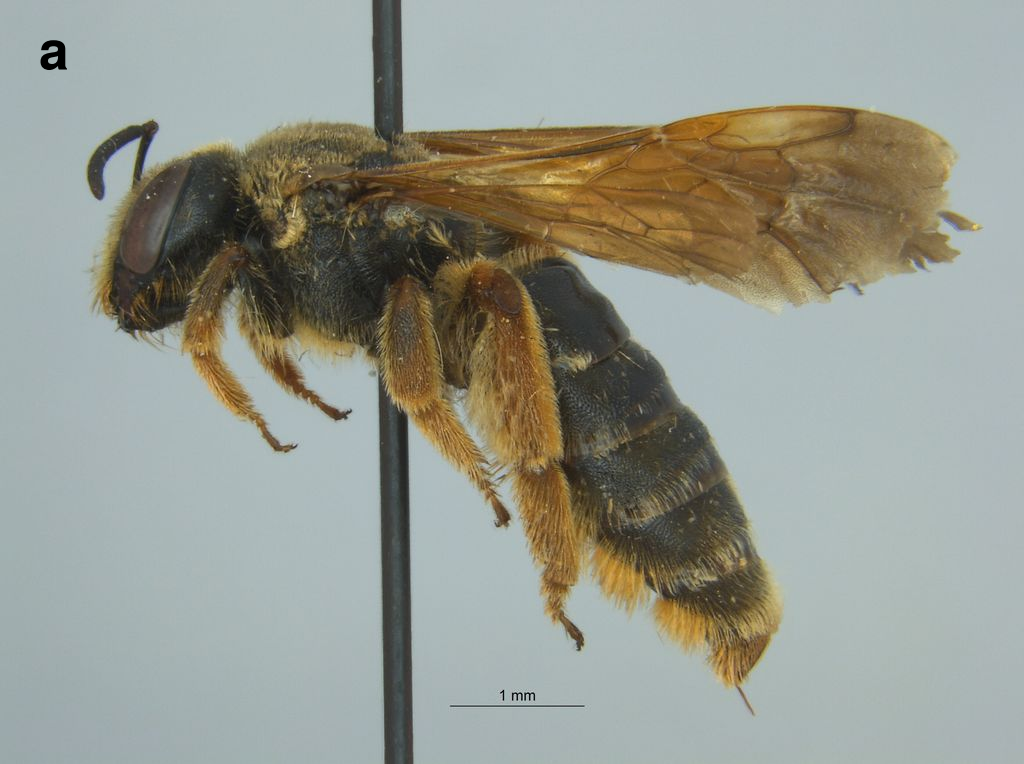
Female, lateral view.

**Figure 4b.**
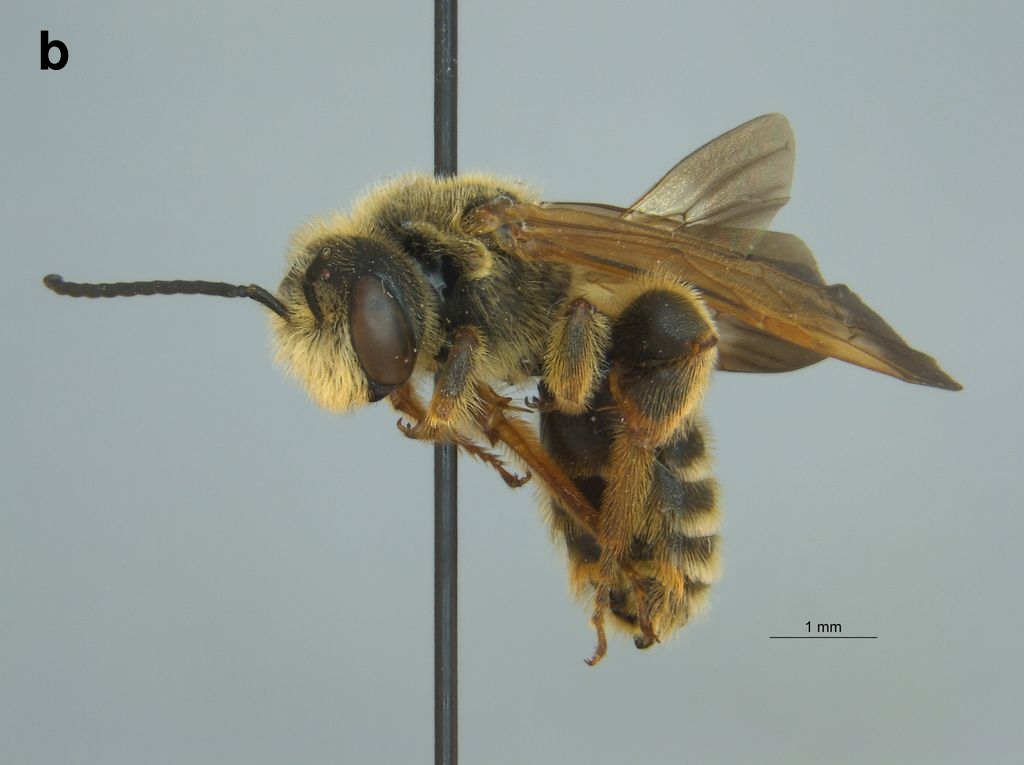
Male, lateral view.

**Figure 4c.**
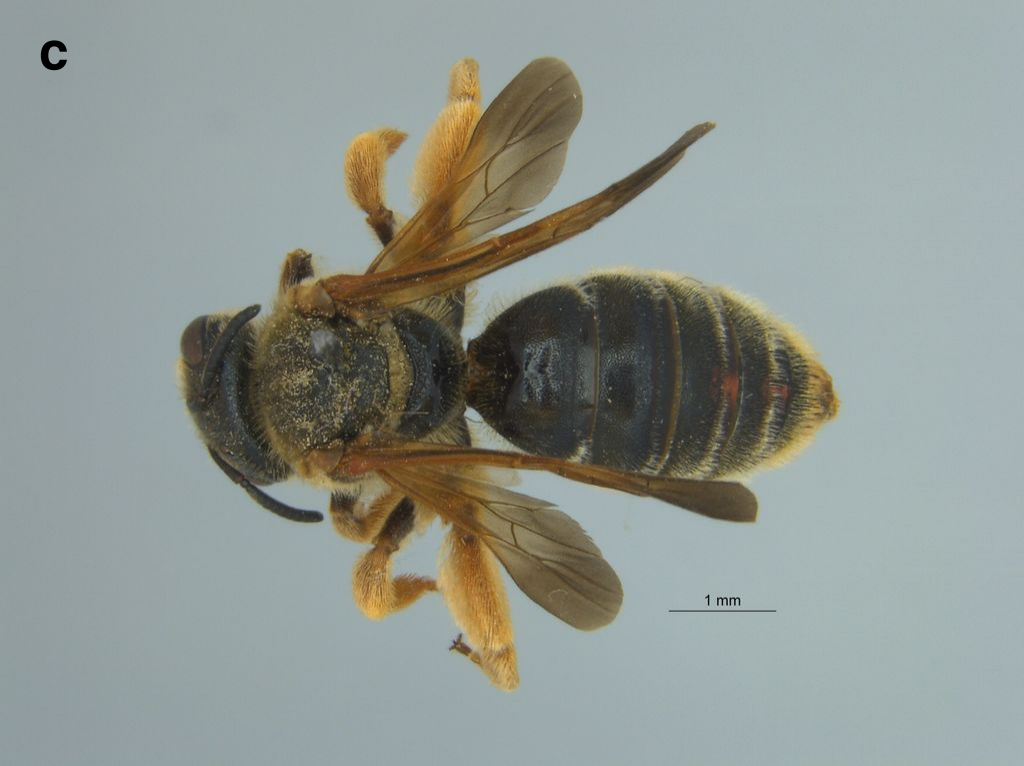
Female, dorsal view.

**Figure 5a.**
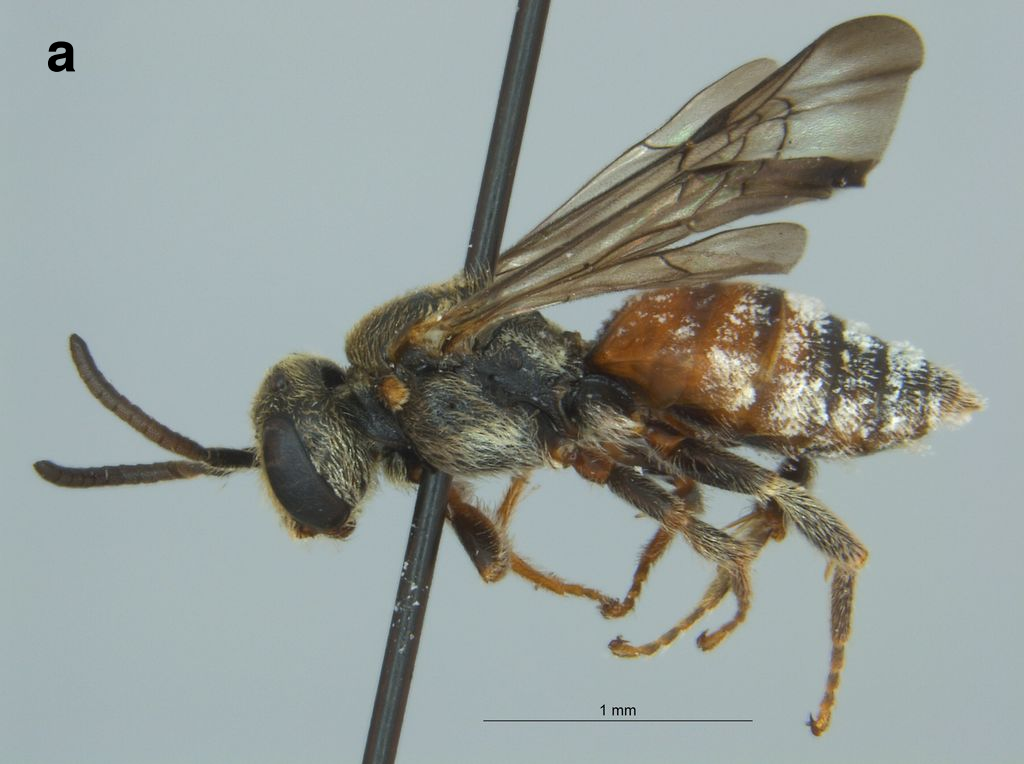
Lateral view.

**Figure 5b.**
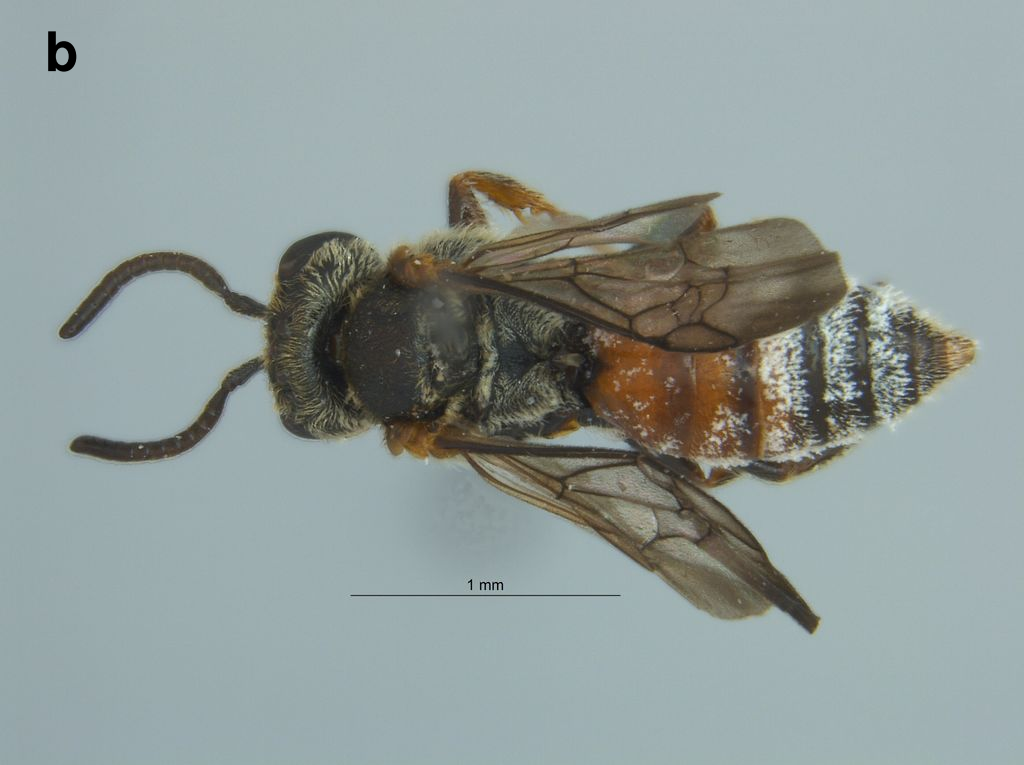
Dorsal view.

**Figure 6a.**
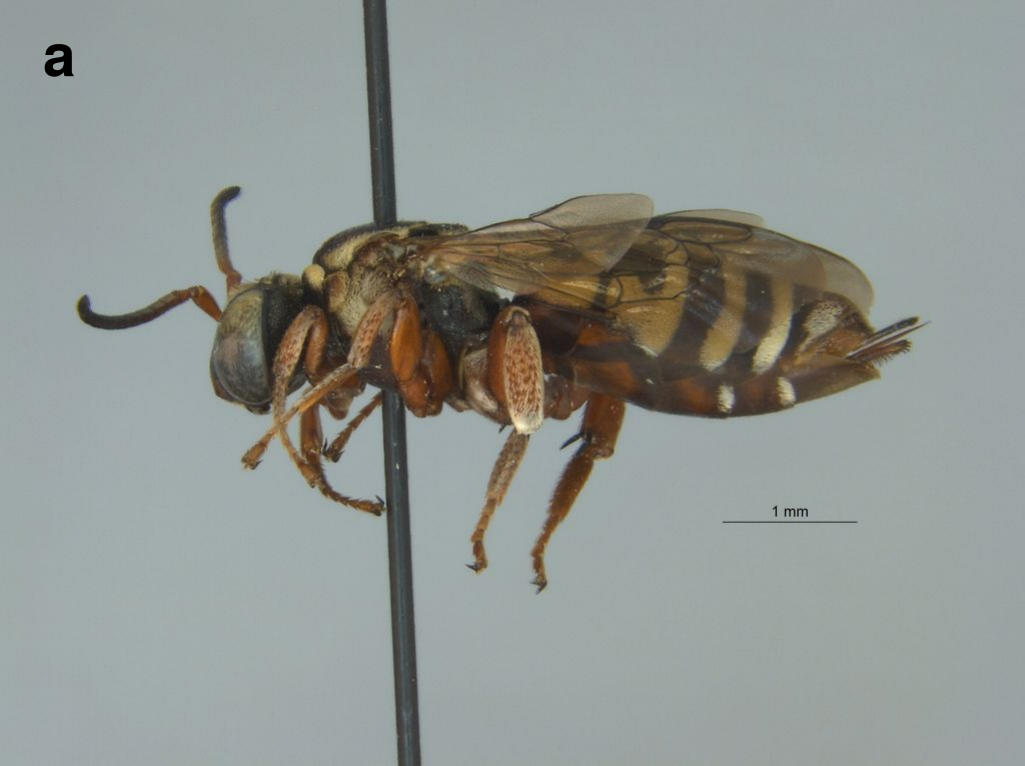
Lateral view.

**Figure 6b.**
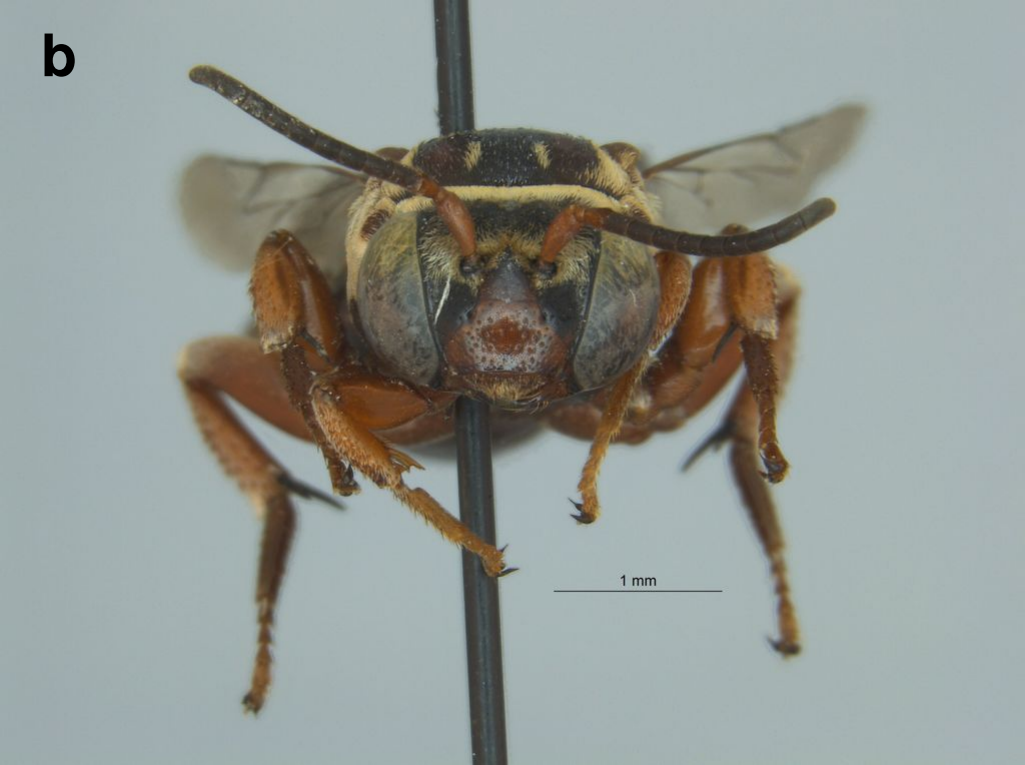
View of head.

**Figure 6c.**
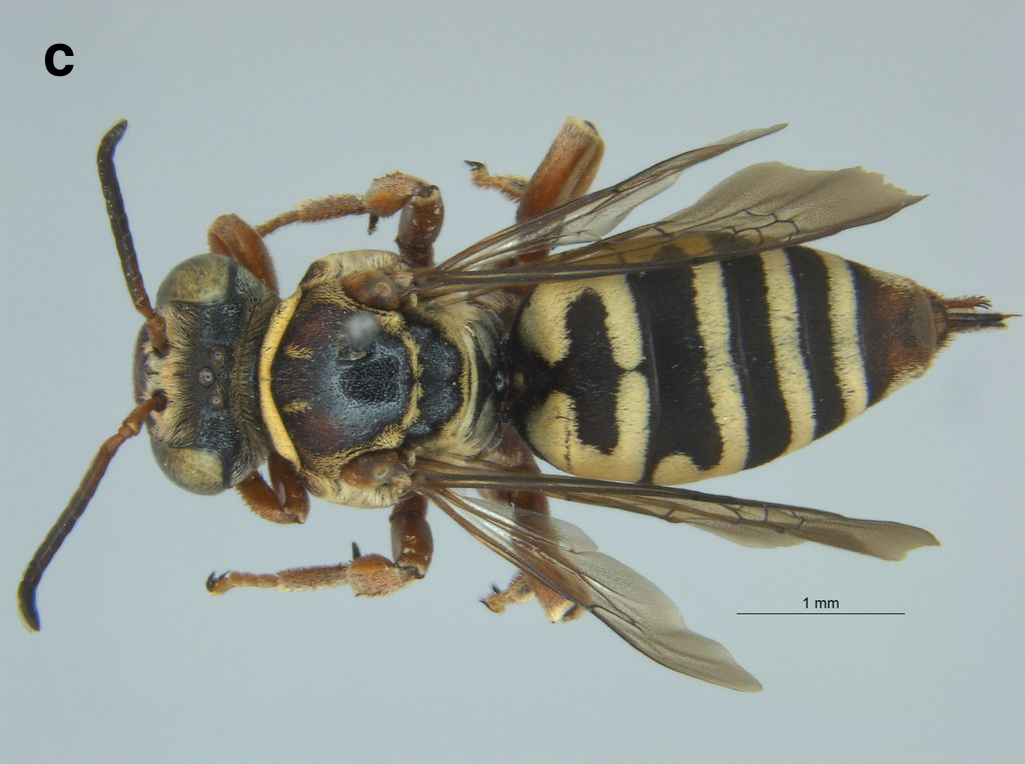
Dorsal view.

**Figure 8a.**
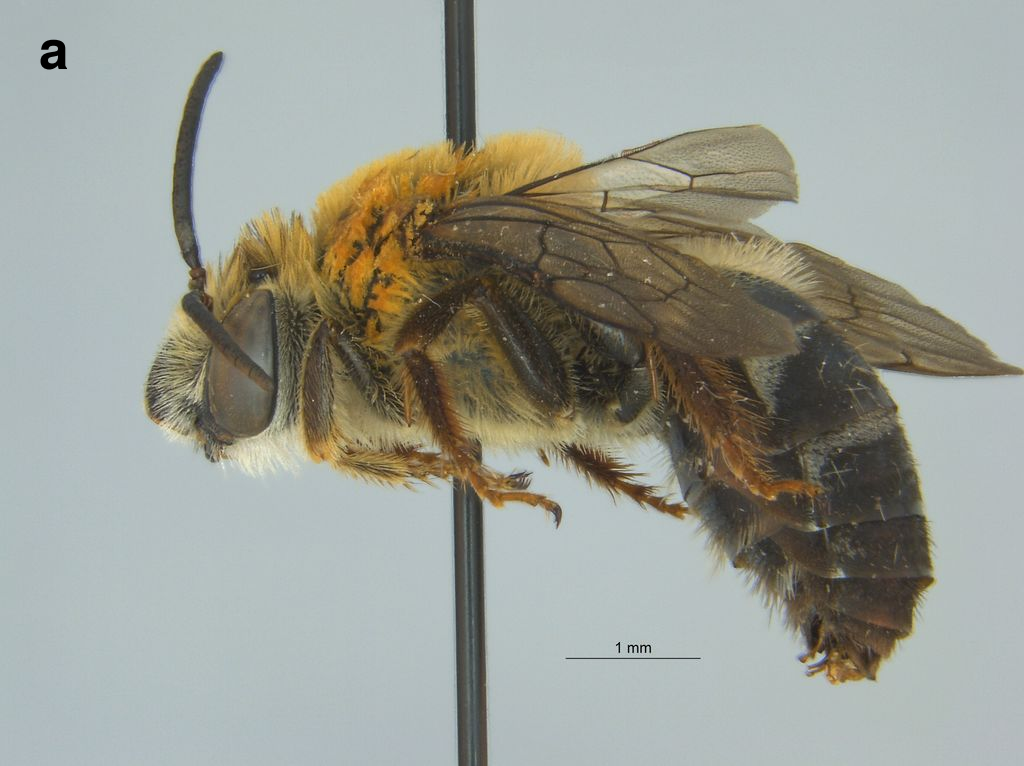
Lateral view.

**Figure 8b.**
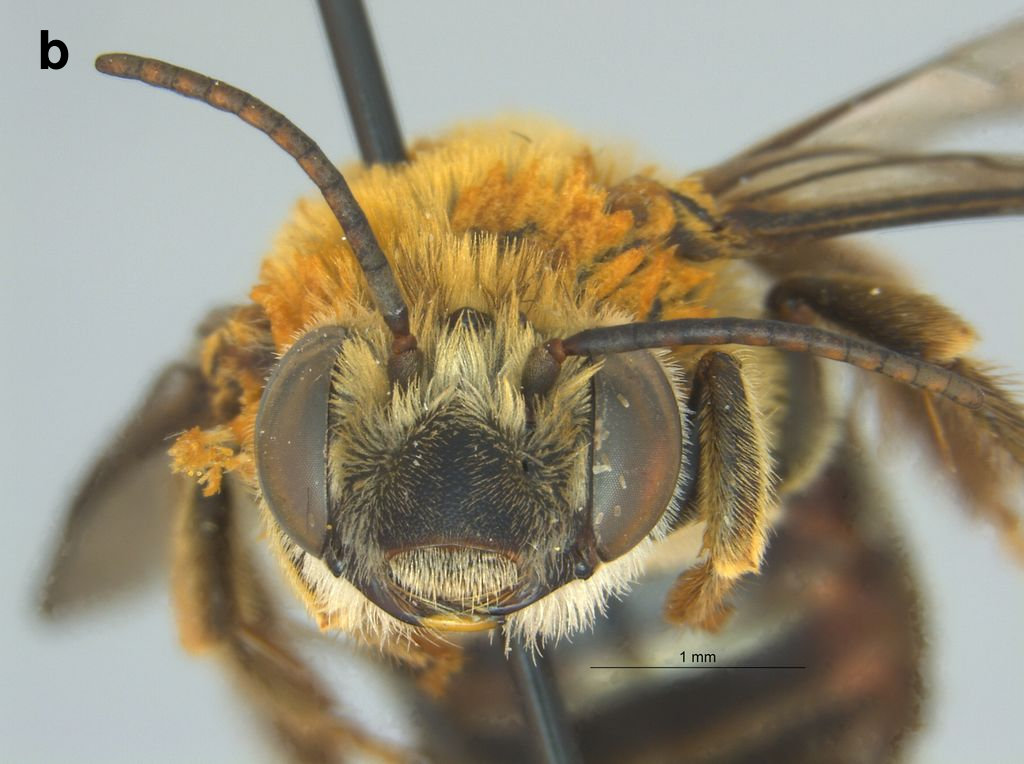
View of head.

**Figure 8c.**
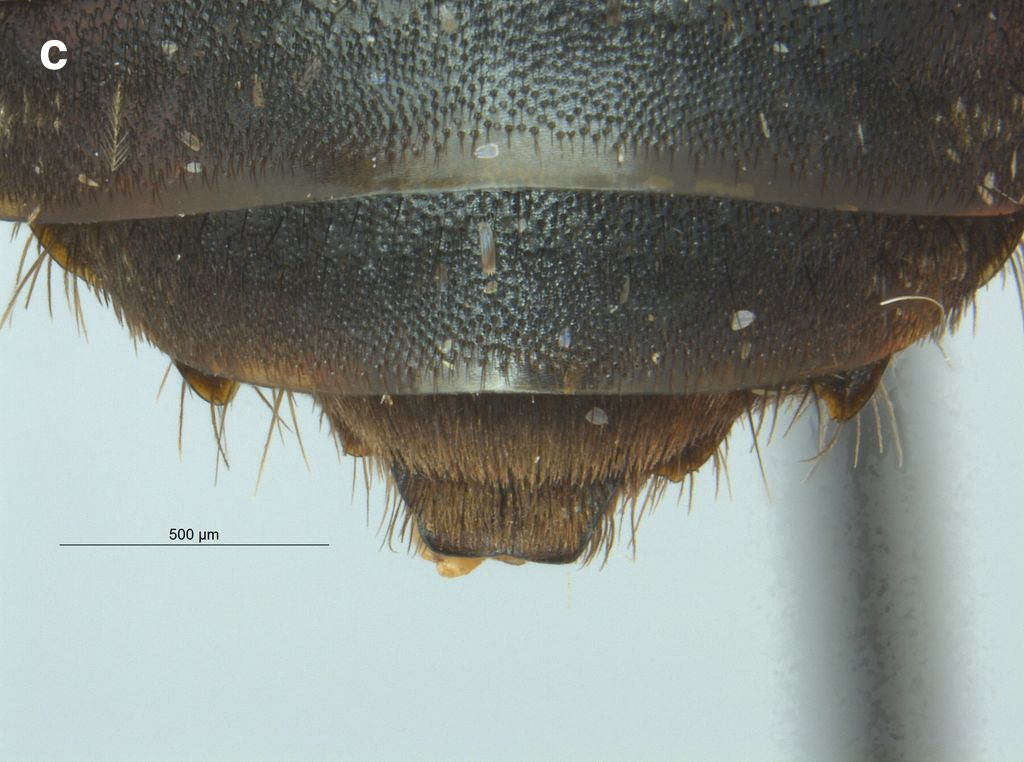
Dorsal view of abdomen showing tooth on T6.
